# Web-Based Single Session Intervention for Perceived Control Over Anxiety During COVID-19: Randomized Controlled Trial

**DOI:** 10.2196/33473

**Published:** 2022-04-12

**Authors:** Michael Mullarkey, Mallory Dobias, Jenna Sung, Isaac Ahuvia, Jason Shumake, Christopher Beevers, Jessica Schleider

**Affiliations:** 1 Department of Psychology Stony Brook University Stony Brook, NY United States

**Keywords:** anxiety, COVID-19, single-session intervention, SSI, perceived control, intervention, mental health, control, online intervention, telemedicine, telehealth, scalable

## Abstract

**Background:**

Anxiety is rising across the United States during the COVID-19 pandemic, and social distancing mandates preclude in-person mental health care. Greater perceived control over anxiety has predicted decreased anxiety pathology, including adaptive responses to uncontrollable stressors. Evidence suggests that no-therapist, single-session interventions can strengthen perceived control over emotions like anxiety; similar programs, if designed for the COVID-19 context, could hold substantial public health value.

**Objective:**

Our registered report evaluated a no-therapist, single-session, online intervention targeting perceived control over anxiety in the COVID-19 context against a placebo intervention encouraging handwashing. We tested whether the intervention could (1) decrease generalized anxiety and increase perceived control over anxiety and (2) achieve this without decreasing social-distancing intentions.

**Methods:**

We tested these questions using a between-subjects design in a weighted-probability sample of US adults recruited via a closed online platform (ie, Prolific). All outcomes were indexed via online self-report questionnaires.

**Results:**

Of 522 randomized individuals, 500 (95.8%) completed the baseline survey and intervention. Intent-to-treat analyses using all randomized participants (N=522) found no support for therapeutic or iatrogenic effects; effects on generalized anxiety were *d*=–0.06 (95% CI –0.27 to 0.15; *P*=.48), effects on perceived control were *d*=0.04 (95% CI –0.08 to 0.16; *P*=.48), and effects on social-distancing intentions were *d*=–0.02 (95% CI –0.23 to 0.19; *P*=.83).

**Conclusions:**

Strengths of this study included a large, nationally representative sample and adherence to open science practices. Implications for scalable interventions, including the challenge of targeting perceived control over anxiety, are discussed.

**Trial Registration:**

ClinicalTrials.gov NCT04459455; https://clinicaltrials.gov/show/NCT04459455

## Introduction

### Background

Social isolation, looming threats of infection, and declining confidence in our abilities to cope have been spurred by the COVID-19 pandemic [1]. For many, this cocktail of stressors has led to increased anxiety. Based on large surveys of health care workers [2], their family members [3], and the general population [4] in China during the COVID-19 pandemic’s escalation, 24%-33% of people met criteria for an anxiety disorder. These rates are roughly double the point prevalence rate of anxiety disorders from a previous representative sample [5].

Likewise, levels of anxiety symptoms appear to be rising among US citizens. The COVID-19 pandemic has had wide and enduring negative effects on the mental health of individuals across the life span [6]. In a nationally representative survey conducted March 11-15, 2020 (n=1216), 32% of American adults reported worry due to COVID-19 had negatively impacted their mental health, and this rate climbed to 45% when the same question was asked in another nationally representative sample conducted March 25-30, 2020 (n=1226) [7].

Certainly, not all of these shifts reflect increases in pathological anxiety (versus situation-appropriate worry), but reasons remain for serious clinical concern. Increased time spent worrying about COVID-19 relates to more severe anxiety pathology—both in health care workers [8] and the general population in China [9]. Increased anxiety symptoms could also have negative public health effects during a pandemic. For example, 28% of people with anxiety disorders seek treatment in emergency rooms each year [10], frequently due to somatic symptoms with no medical cause (eg, panic symptoms like unspecified chest pain) [11]. As anxiety rates increase, so too could these often-unnecessary hospital visits, further exacerbating patient burden in already overwhelmed emergency medicine departments. Identifying an intervention to facilitate independent coping with anxiety—ideally, one that is brief and easily scalable—could help mitigate negative effects of increasing anxiety nationwide.

Perceived control, or one’s subjectively felt ability to control one’s environment and inner experiences, prospectively predicts lower distress during and following numerous uncontrollable stressors, from experiencing sexual assault [12] to recovering from breast cancer surgery [13] and a heart attack [14]. If one perceives control over their ability to reduce anxious responses (eg, racing thoughts, pounding heart), theory suggests that one is likely to experience less distress, regardless of actual control [15,16]. Empirical evidence consistently supports this idea. Individuals reporting lower perceived control of their internal experiences exhibit higher levels of anxiety (ranging from nonclinical to clinical levels), regardless of objective levels of control [17,18]. Adults in community and nonclinical samples reporting lower perceived internal control have shown higher prevalence rates of anxiety disorders and more severe anxiety symptomatology versus those reporting higher levels of perceived internal control [19-21]. With respect to prospective associations [22], lower levels of perceived internal control have predicted higher future anxiety symptom severity in adults (including both social and generalized anxiety severity). Likewise, a meta-analysis exploring low perceived control as a transdiagnostic risk factor for anxiety disorders [23] found, across studies and diagnoses, perceived control was negatively linked with both trait and pathological anxiety severity. Experimental evidence suggests that one can reliably decrease anxiety related to low perceived internal control by increasing one’s capacity to alter their own thoughts, emotions, and experiences—for instance, by teaching specific skills or strategies to manage inner experiences [24-27].

During the present pandemic, one’s perceived control over the circumstances may be (and remain) understandably low: No individual can slow its course single-handedly. In fact, perceived control of one’s environment is largely unrelated to anxiety following circumstantial stressors (eg, undergoing basic military training) [28]. However perceived control over one’s own anxiety may remain high—and can be augmented—even amid uncontrollable circumstances like a psychiatric hospitalization [29]. In contrast with perceived control over one’s environment, perceived control over one’s own anxiety is negatively associated with generalized anxiety symptoms, even after controlling for the Big Five personality traits [22], and prospectively predicts fewer daily internalizing symptoms over the course of a month [30]. Psychosocial treatments such as intensive psychiatric hospitalization [29] and acceptance and commitment therapy [31] appear to increase perceived control of anxiety, and these increases are associated with declines in anxiety symptoms during treatment. Although we cannot be certain perceived control will demonstrate the same links to anxiety symptoms during a population-level uncontrollable event (eg, a global pandemic), it is a promising preliminary target for intervention. Importantly, preliminary evidence suggests that a self-administered, online, and single session intervention (SSI) can increase perceived control over emotions like anxiety from pre- to postintervention [32], in turn predicting decreases in anxiety severity 9 months later [33].

SSIs consistently demonstrate similar effect sizes to multisession therapies on mental health outcomes [34-36]. Meta-analytic evidence also indicates self-administered, online SSIs yield similar effect on mental health outcomes as therapist-directed SSIs [34,37]. Brief interventions that teach simple, repeatable skills, such as goal setting, may exert larger effects on psychopathology compared with “information-only” and norm-referencing interventions [37]. In fact, early research identifies a combination of (1) normalizing experiences via neuroscientific explanations of mental health difficulties (with care to not communicate these difficulties are thereby inherent and unchangeable [38]), (2) providing testimonials from others to reinforce this norm and introduce examples of repeatable skill use, and (3) empowering participants as helpers by asking them to practice the repeatable skill during the session and share their advice for how to implement the repeatable skill with others [39], as potentially helpful components of self-administered SSIs. This format allows for “minimal” interventions that retain efficacy [40-42]. Indeed, interventions as short as 5 minutes can improve mental health–related outcomes [37], consistent with findings that interventions of similar length can improve academic performance [43] and increase later egalitarian actions [44]. This intervention format can also more effectively scale up to meet the mental health needs of large numbers of people than traditional face-to-face therapy or longer online treatments [45,46].

Online, self-administered SSIs are also more easily, rapidly testable in representative samples than interventions requiring clinician contact (either in-person or remotely). Clinical trials of mental health treatments are generally underpowered [47] and nonrepresentative of the general population [48]. In larger, more representative clinical trials of clinician-dependent treatments, the recruitment process often requires several years [49]. This timeline is wholly incompatible with testing interventions to mitigate harms of immediate crises, including the COVID-19 pandemic. Rather, such tests require interventions that may be evaluated rapidly, iterated if necessary, and disseminated appropriately while the pandemic is still ongoing. Online, self-administered SSIs fulfill these criteria, as there is evidence weighted-probability samples can be collected in 2 to 3 days for survey research [50], and SSIs found to be efficacious could be disseminated immediately, and broadly, online [51]. Even if participants are half as willing to complete an SSI program embedded within a survey, compared with a survey on its own, baseline data collection could still be completed in less than 1 week.

We therefore evaluated whether an online, self-administered SSI designed to strengthen perceived control over anxiety in the context of the COVID-19 pandemic (Contain COVID Anxiety) increased perceived control over anxiety immediate post-SSI and decreases general anxiety 2 weeks later more than a placebo, handwashing-plan SSI (Remain COVID Free) in a weighted-probability sample of the United States (n=500, See Multimedia Appendix 1 for the full text of both SSIs). See Table 1 for all confirmatory hypotheses and guidelines for interpretations of results.

Our primary hypothesis concerned whether the Contain COVID Anxiety SSI decreased generalized anxiety symptoms 2 weeks later more than the placebo SSI.

**Table 1:** Design table.

Question and hypotheses			Sampling plan (eg, power analysis)^a^		Analysis plan^a^		Interpretation given to different outcomes
**Does the Contain COVID Anxiety SSI^b^ decrease generalized anxiety symptoms from baseline to 2 weeks later more than the placebo SSI Remain COVID Free?**							
	H1: Generalized anxiety decreases more when participants are randomized to the Contain COVID Anxiety SSI than when participants are randomized to the Remain COVID Free placebo SSI.	H1: n=400 for 95% power		H1: Test for assumptions and apply transformations as necessary. Take the 2-week follow-up generalized anxiety mean and enter it as the dependent variable in a linear model with the baseline generalized anxiety mean and treatment condition as predictors.		Support for H1: If the *P* value for condition is <.0167 in the H1 linear model and the 95% CI for the difference in generalized anxiety is negative and does not include 0 when participants are randomized to the Contain COVID Anxiety SSI, we will reject H0 and interpret the study as supporting Contain COVID Anxiety decreasing generalized anxiety more than the placebo SSI Remain COVID Free. Lack of support for H1: If the *P* value for the equivalence test described in the “Support for H0” section is >.0167, we will interpret the study as producing evidence that the Contain COVID Anxiety SSI is neither superior nor equivalent or inferior to the Remain COVID Free SSI at decreasing generalized anxiety.	
	H0: Generalized anxiety either does not increase more when participants are randomized to the Contain COVID Anxiety SSI than when participants are randomized to the Remain COVID Free placebo SSI, or generalized anxiety increases more when participants are randomized to the Remain COVID Free placebo SSI than when participants are randomized to the Contain COVID Anxiety placebo SSI.	H0: n=150 for 95% power		H0: Test for assumptions and apply transformations as necessary. Take the 2 weeks later generalized anxiety mean and predict it with the baseline generalized anxiety mean. Enter the mean and SD of the standardized residuals from that model for when the condition is Remain COVID Free and the mean and SD of the standardized residuals from that model when the condition is Contain COVID Anxiety into a between-groups equivalence test with equivalence bounds of *d*=–0.66 to *d*=0.33.		Support for H0: If the *P* value for condition is <.0167 in the H1 linear model and the 95% CI for the difference in generalized anxiety is positive and does not include 0 when participants are randomized to the Remain COVID Free SSI or the *P* value or intervention order is >.0167 in the H1 linear model, we will run the between-groups equivalence test described in the analytic plan for H0. If the equivalence test has a *P* value <.0167, we will interpret the results as indicating the Contain COVID Anxiety SSI was equivalent or inferior to the Remain COVID Free SSI at improving generalized anxiety. Lack of support for H0: If the *P* value for the equivalence test described in the “Support for H0” section is >.0167, we will interpret the study as producing evidence that the Contain COVID Anxiety SSI is neither superior nor equivalent or inferior to the Remain COVID Free SSI at decreasing generalized anxiety.	
**Does the Contain COVID Anxiety SSI increase perceived control over anxiety from baseline to immediately post-SSI more than the placebo SSI Remain COVID Free?**							
	H1: Perceived control over anxiety increases more when participants are randomized to the Contain COVID Anxiety SSI than when participants are randomized to the Remain COVID Free placebo SSI.	H1: n=350 for 95% power		H1: Test for assumptions and apply transformations as necessary. Take the post-SSI perceived control over anxiety mean and enter it as the dependent variable in a linear model with baseline perceived control over anxiety mean and treatment condition as predictors.		Support for H1: If the *P* value for condition is <.0167 in the H1 linear model and the 95% CI for the difference in perceived control over anxiety is positive and does not include 0 when participants are randomized to the Contain COVID Anxiety SSI, we will reject H0 and interpret the study as supporting Contain COVID Anxiety increasing perceived control over anxiety more than the placebo SSI Remain COVID Free. Lack of support for H1: If the *P* value for the equivalence test described in the “Support for H0” section is >.0167, we will interpret the study as producing evidence that the Contain COVID Anxiety SSI is neither superior nor equivalent or inferior to the Remain COVID Free SSI at improving perceived control over anxiety.	
	H0: Perceived control over anxiety either does not increase more when participants are randomized to the Contain COVID Anxiety SSI than when participants are randomized to the Remain COVID Free placebo SSI or perceived control over anxiety increases more when participants are randomized to the Remain COVID Free placebo SSI than when participants are randomized to the Contain COVID Anxiety placebo SSI.	H0: n=150 for 95% power		H0: Test for assumptions and apply transformations as necessary. Take the post-SSI perceived control over anxiety mean and predict it with the baseline perceived control over anxiety mean. Enter the mean and SD of the standardized residuals from that model for when the condition is Remain COVID Free and the mean and SD of the standardized residuals from that model when the intervention order is Contain COVID Anxiety into a between groups equivalence test with equivalence bounds of *d*=–0.63 to *d*=0.21.		Support for H0: If the *P* value for condition is <.0167 in the H1 linear model and the 95% CI for the difference in perceived control over anxiety is negative and does not include 0 when participants are randomized to the Remain COVID Free SSI or the *P* value or intervention order is >.0167 in the H1 linear model, we will run the between-groups equivalence test described in the analytic plan for H0. If the equivalence test has a *P* value <.0167, we will interpret the results as indicating the Contain COVID Anxiety SSI was equivalent or inferior to the Remain COVID Free SSI at improving perceived control over anxiety. Lack of support for H0: If the *P* value for the equivalence test described in the “Support for H0” section is >.0167, we will interpret the study as producing evidence that the Contain COVID Anxiety SSI is neither superior nor equivalent or inferior to the Remain COVID Free SSI at improving perceived control over anxiety.	
**Does completing the Contain COVID Anxiety SSI have an association with social distancing intentions statistically equivalent to 0?**							
	H1: Social distancing intentions do not increase or decrease pre-Contain COVID Anxiety SSIs to immediate post-Contain COVID Anxiety SSI.	H1: n=154 for 95% power		H1: Test for assumptions and apply transformations as necessary. Enter the mean and SD of the social distancing intention composites at baseline and post-Contain COVID Anxiety SSI (only among people who were randomized to the Contain COVID Anxiety) into a paired equivalence test with equivalence bounds of *d*=–0.33 to *d*=0.33.		Support for H1: The *P* value for the paired equivalence test described in the analysis plan column is <.0167. We will interpret this result as support for the hypothesis that social distancing intentions are not increased or decreased pre to post both Contain COVID Anxiety (even if the paired t test for H0 in the analysis plan column also has a *P* value <.0167).	
	H0: Social distancing intentions either increase or decrease pre- to immediate post-Contain COVID Anxiety SSI	H0: n=156 for 95% power		H0: Test for assumptions and apply transformations as necessary. Enter the mean and SD of the social distancing intention composites at baseline and post-Contain COVID Anxiety SSI (only among people who were randomized to the Contain COVID Anxiety) into a paired *t* test.		Support for H0: The *P* value for the paired equivalence test in the analysis plan column is >.0167, and the *P* value for the paired *t* test in the analysis plan column is <.0167. We will interpret this result as supporting the hypothesis that social distancing intentions either increased or decreased as the result of completing the Contain COVID Anxiety SSI. We will examine the direction of the effect by looking at the direction of the effect size (positive effect size = increase in social distancing intentions; negative effect size = decrease in social distancing intentions).	

^a^Refer to the R code on the open science framework page for the power analysis and analysis plan [52].

^b^SSI: single-session intervention.

### Hypothesis 1

We expected a larger decrease in generalized anxiety symptoms from immediately pre-SSI to 2 weeks later when participants were randomized to the Contain COVID Anxiety SSI instead of the placebo SSI. This pattern of results would indicate a larger decrease in generalized anxiety symptoms occurs pre-SSI to 2 weeks later in the Contain COVID Anxiety SSI compared with the placebo SSI.

We also tested whether the active SSI Contain COVID Anxiety can be deployed at scale without reducing intentions to engage in social distancing. Social distancing remains the most potent known nonpharmacological intervention to reduce the spread of the SARS-Cov-2 virus [53]. Directly testing whether completing Contain COVID Anxiety has the negative side effect of reduced social distancing intentions, which could in turn predict reduced social distancing behaviors [54], is crucial to determining whether the intervention can be responsibly tested and disseminated at scale. Thus, we developed this intervention with an eye toward not undermining social distancing intentions. However, social distancing intentions were not a direct target of the intervention, so we also did not expect to see an increase in these intentions as a result of completing the intervention.

### Hypothesis 2

We hypothesized that engaging in the Contain COVID Anxiety SSI would have an association with pre- to post-SSI change in social distancing intentions that is statistically equivalent to 0. This pattern of results would indicate completing the intervention is not meaningfully associated with intentions to socially distance.

We were also interested in whether the SSI designed to increase perceived control over anxiety did, in fact, increase perceived control over anxiety immediate post-SSI more than the placebo SSI.

### Hypothesis 3

We expected a larger increase immediate pre- to immediate post-SSI in perceived control over anxiety to occur for participants randomized to the Contain COVID Anxiety SSI, relative to those randomized to the placebo SSI. This pattern of results would indicate a larger increase in perceived control over anxiety occurs pre- to post-Contain COVID Anxiety SSI compared with the placebo SSI.

### Present Study

This study tested the efficacy of decreasing generalized anxiety symptoms (Hypothesis 1) and increasing perceived control of anxiety with the Contain COVID Anxiety SSI in a nationally representative sample (Hypotheses 1 and 3) and the safety of testing and making the SSI available at scale (Hypothesis 2). This is a crucial step toward providing open-access, evidence-based resources to help the US population more effectively cope with their anxiety during the COVID-19 pandemic.

## Methods

### Ethics Information

The study was approved by the Institutional Review Board (IRB) of Stony Brook University under protocol #2020-00204. As required by US law, a description of this study is available at Clinicaltrials.gov. We have also included a CONSORT checklist in Multimedia Appendix 2.

### Participants and Design

We recruited a sample of 500 participants representing a weighted-probability sample of the United States through Prolific, an online platform connecting researchers and participants. Prolific is designed specifically for use in the scientific research context, unlike older online crowdsourcing platforms (eg, Mechanical Turk) that were designed for broader use (eg, by marketing and advertising companies to outsource labor) [55,56]. To address particular needs of the academic research community, Prolific provides estimates of the available population for a given study, enables confidential collection of human subjects data, allows for prescreening and exclusion of participants based on individual study criteria, prevents duplicate responses by limiting users to one Prolific account that is verified by built-in data quality checks using cookies and IP address, and directly facilitates longitudinal data collection [56]. Upon signing up via Prolific to volunteer for scientific research studies (ie, click a button on the Prolific website that reads, “Participate: Take part in engaging research, earn cash, and help improve human knowledge!”), Prolific users provide sociodemographic and personal background information; they then receive invitations via email to take part in studies for which they qualify, whenever such studies are made available by research teams around the world. To date, Prolific has been used in hundreds of psychological scientific studies, including many focused on adults experiencing mental health problems [57,58]. Prolific allows for informed consent to be provided digitally. In this study, individuals volunteered to participate by providing digital informed consent within an online Qualtrics survey developed by the study team, after being presented with an IRB-approved study information and consent form. This consent form included all relevant information about the benefits, risks, and compensation related to study participation. University affiliation (Stony Brook University) was visible to participants on consent forms and on the first page of both SSI programs (Contain COVID Anxiety SSI and the placebo Remain COVID Free SSI). Once a user completed the study, the project was no longer viewable on their Prolific account.

This study’s weighted-probability sample was stratified on age, sex, and ethnicity. To help further maximize the generalizability of our findings, there were no prescreening inclusion nor exclusion criteria other than having a Prolific ID, being at least 18 years old (able to provide consent), and residing in the United States. However, study participation required access to, and comfort using, a device connected to the Internet. We also recruited 8 pilot participants from the Prolific platform before recruiting this weighted probability sample to ensure data were being collected properly, and these pilot participants’ data were not used in confirmatory analyses of this study. All pilot data are available on the open science framework page [52].

We conducted intent-to-treat analyses including all participants who were randomized to a study condition (n=522, see [52]). We sought to prevent missing data by requesting responses to each question (with a reminder at the end of each page if participants had not answered a question) and imputed missing data using the expectation-maximization and bootstrapping algorithm implemented with Amelia II in R [59].

We used a between-subjects design; participants were randomized to receive either the active Contain COVID Anxiety SSI (50% allocation) or the placebo Remain COVID Free SSI (50% allocation). The sequence determining randomization of condition was automatically generated using the randomizer within Qualtrics Survey Software (no blocking was used for this randomization), making the randomization process double-blind. To triple-blind our analysis process, the last author (JS) downloaded the data from Qualtrics and recoded the variable indicating to which SSI the participants were randomized before sending the data to the first author (MM) who performed the primary analyses. Therefore, the primary analyses were conducted without the knowledge of which condition is which.

Power analyses were conducted using a “smallest effect size of interest” approach, where we aimed to be powered to detect the smallest effect size corresponding with a subjectively meaningful difference in participants’ experiences [60]. For hypothesis 1, using simulations conducted in R, we determined we would need a sample size of 400 for 95% power to detect the smallest effect size of interest for this hypothesis (*d*=0.33, as determined by a conservative estimate from a previous SSI for general anxiety). For hypothesis 2, using the TOSTER package in R, we determined we would have >95% power to detect whether the beginning to end effect of the Contain COVID Anxiety SSI on social distancing intentions falls within the equivalence bounds of *d*=–0.33 to *d*=0.33 with an n of 250. We chose these equivalence bounds based on not wanting any negative side effects on social distancing intentions to be greater than the smallest effect size of interest for our primary outcome (general anxiety). For hypothesis 3, using simulations conducted in R, we determined we would have >95% power to detect the smallest effect size of interest, *d*=0.21, the smallest change in perceived control due to an intervention to predict later decreases in anxiety observed in previous SSI work at n=500 [33]. We retained greater than 95% power by both recruiting enough participants to account for 20% attrition at the 2-week follow-up and using multiple imputation techniques to carry out an intent-to-treat approach. Further, we also conducted sensitivity tests for each hypothesis to examine the range of estimates of the effects observed if all missing data are assumed to be in either the 75th or 25th percentile of change for each key variable—thereby quantifying what our estimates would look like if our data were not missing at random due to unobserved confounders. See the publicly available code for the power analysis [52].

### Procedure

The entire procedure was conducted online via the Qualtrics Survey Platform, which participants were linked to directly from Prolific. After providing informed consent, participants spent approximately 8 minutes filling out pre-intervention questionnaires including demographics, depression symptoms, generalized anxiety symptoms, self-hatred, access to mental health treatment, and COVID-19–related stressors.

Immediately following answering these questions and immediately prior to the SSI intervention, participants completed the Anxiety Control Questionnaire-Emotion Control (ACQ-EC) scale, the Hand Washing Intentions scale, and several questions about social distancing asked in national surveys to measure their beliefs about the intentions of others to engage in social distancing behaviors like avoiding public spaces and private gatherings.

The participants were then randomized to and spent approximately 8 minutes completing one of the SSIs described in the following sections (which one depended on the number generated by the random sequence from the Qualtrics randomizer described in the previous paragraphs), immediately followed by approximately 2 minutes completing the ACQ-EC scale, the Hand Washing Intentions scale, several questions about social distancing from a standardized Centers for Disease Control and Prevention (CDC) item bank, and the comprehension questions. They were then sent to a Prolific link for compensation (with an incentive of US $2.17 for the 20-minute survey, or US $6.50 per hour) and reminded of the follow-up survey 2 weeks later. Participants also received a reminder through the Prolific platform 2 weeks later to participate in the 2-minute follow-up survey containing a measure of generalized anxiety symptoms, perceived control over anxiety, and an anchor-based question (see [60]) to help determine the smallest subjectively noticeable difference in generalized anxiety symptoms over 2 weeks. They were then debriefed, provided with mental health resources, and sent to a Prolific link for compensation (US $0.22 for the 2-minute survey, or US $6.50 per hour). We focus here on describing scales directly related to confirmatory hypotheses and quality checks. See Multimedia Appendix 1 for a list of questionnaires included.

### Measures

#### Anxiety Symptoms

The Generalized Anxiety Disorder-7 (GAD-7; [61]) measures clinical anxiety symptom severity, based on diagnostic criteria for generalized anxiety disorder. The GAD-7 includes 7 items asking respondents how often, during the last 2 weeks, they were bothered by each of 7 anxiety symptoms. Response options are “not at all,” “several days,” “more than half the days,” and “nearly every day,” scored as 0, 1, 2, and 3, respectively; thus, total sum-scores may range from 0 to 21, and average scores range from 0 to 3. The GAD-7 has shown adequate reliability and strong convergent validity with other anxiety scales [61]. The GAD-7 is frequently used in large-scale treatment and dissemination studies as a generic measure of change in anxiety symptoms [62].

#### Perceived Control Over Anxiety

The ACQ-EC [19] measures how much perceived control participants have over their anxiety, the primary outcome of the study. It is one of the 3 validated subscales of the Anxiety Control questionnaire and contains 4 items (eg, “I am able to control my level of anxiety.”) rated on a 0 (“Strongly Disagree”) to 5 (“Strongly Agree”) scale. The potential mean scores of the scale (the score of interest for testing hypothesis 1 at all 3 times points) therefore range from 0 to 5. The scale has a well-validated factor structure in a nonclinically selected sample, is strongly associated with anxiety and depression symptoms, and has demonstrated good internal consistency in previous investigations [19].

#### Social Distancing Intentions

The following Social Distancing Intentions questions, the secondary outcome of the study, are part of a standardized item bank provided by the CDC [63]: All start with “Starting today, for how long do you believe others would be willing to engage in the following behaviors?” and then “Avoid going out to a restaurant, bar, or club,” “Avoid going to a family gathering like a birthday party or wedding or funeral,” or “Avoid going to a social gathering with friends, peers, or coworkers (not including relatives)” on a scale from 1 (“Less than a month”) to 4 (“4 months or more”). As validated measures for social distancing intentions do not yet exist, we propose to use these questions given these items are drawn from a standardized item bank provided by the CDC to better facilitate cumulative science (as other researchers will also utilize these items). At the suggestion of a reviewer, we changed the wording of these questions to ask about participants’ beliefs about others’ willingness to engage in these behaviors to reduce potential social desirability bias in responding. The potential mean scores of the scale (the score of interest for testing hypothesis 2 at both time points) therefore range from 1 to 4.

#### Comprehension Questions

We used comprehension questions as an initial quality check to ensure participants comprehended the core messages of both SSIs. These questions go beyond traditional attention check items, which can be answered incorrectly even by attentive participants [64]. Following each intervention, we asked 2 multiple choice questions with 4 potential response options—1 correct answer, 1 incorrect answer that contains material from the intervention not relevant to answering the current question, and 2 incorrect responses referencing material not contained in the intervention. The exact questions can be found in Multimedia Appendix 1. We initially required at least 75% of participants to answer both comprehension check questions correctly following the Contain COVID Anxiety SSI to pass the quality check, though see the “Comprehension Check Questions” section for further discussion as these questions did not appear to index intervention fidelity in this context.

### Single-Session Interventions

#### Contain COVID Anxiety SSI

This active SSI was developed following current recommendations for evidence-based SSI design to target mental health–related outcomes [39]. Participants first received normalizing scientific information (including neuroscience findings) that help explain why increased anxiety during the COVID-19 pandemic is a typical response. They then read testimonials from 3 other people in the United States who have applied a 3-step action plan for coping more effectively with their anxiety. These 3 steps were (1) reminding themselves increased anxiety is a typical response during a pandemic before writing down some anxiety-provoking events they can’t control and some anxiety-provoking events they can control, (2) picking one of the anxiety-provoking events they can control, and (3) deciding on 1 small step to cope more effectively with the anxiety-provoking event they can control chosen in step 2. Participants were then empowered as helpers by us asking for their permission to share their action plan with others to help them more effectively cope with pandemic-related anxiety. The entire intervention took approximately 8 minutes and was completed entirely within the Qualtrics survey platform.

#### Remain COVID Free SSI

This placebo SSI was developed to mirror the structure of the Contain COVID Anxiety SSI, discuss COVID-19–related content, and do so without as many of the potential active ingredients of effective SSIs. Participants received scientific information about how soap kills the SARS-CoV-2 virus but no neuroscience information related to behaviors or behavior change. Participants were told didactically that there is only one way to wash their hands effectively, by following this 3-step plan: (1) deciding on10 times a day to wash their hands, (2) putting reminders in their calendar or setting alarms on their phone to remind them to wash their hands, and (3) singing happy birthday to their favorite celebrity twice while washing their hands. They then read 3 testimonials from other people who had implemented this plan, but they did not make a plan themselves. They therefore also did not have the opportunity to share their plan to prosocially help others. The entire intervention took approximately 8 minutes and was completed entirely within the Qualtrics survey platform.

### Analysis Plan

#### Testing Participant Dropout

We first tested for dropout from the study due to intervention assignment. For example, people could differentially drop out when receiving the active Contain COVID Anxiety. Thus, we tested for differential dropout using a *z* test of differential proportions, in which we compared the proportion of people who dropped out before completing the study (0 = No, 1 = Yes) as a function of treatment condition (0 = Remain COVID Free, 1 = Contain COVID Anxiety). If the *P* value for this test was <.05, we planned to interpret that dropout as dependent on condition assignment and preregistered that we would not be able to interpret the effects of intervention assignment on outcome (ie, we would not be able to test Hypotheses 1 and 3). If the *P* value was >.05 for this test, we preregistered that we would assume dropout was not dependent on condition assignment.

#### Data Aggregation for Hypothesis Testing

We then created 2 separate scores for the GAD-7 to reflect baseline and 2 weeks post-SSI scores by taking the mean of the 7 items at each time point (score range at each time point: 0-3). We then created 2 separate scores for the ACQ-EC to reflect baseline and immediate post-SSI by taking the mean of the 4 items at each time point (score range at each time point: 0-5). We also calculated the mean of the 3 Social Distancing Intentions questions (score range at each time point: 1-4) at baseline and immediate post-SSI to calculate composite social distancing intentions scores. Following the creation of these composites, we imputed any missing data using the expectation-maximization and bootstrapping algorithm implemented with Amelia II in R [59]. These imputed data sets allowed for more conservative intent-to-treat analyses than listwise deletion or last-observation carried forward [65]. We imputed as many data sets as there were percentages of missing data for an outcome—rounding up to the next highest percentage (eg, If 2.4% of data were missing on an outcome, we created 3 imputed data sets). This process allowed us to retain high power even in the presence of missing data [66].

Consistent with best practices, we included all predictors from the statistical model (baseline value of imputed outcome, either perceived control over anxiety or social distancing intentions, and intervention order) and all baseline variables expected to be associated with the outcome variable (for generalized anxiety and perceived control over anxiety: Inventory of Depression and Anxiety Symptoms Dysphoria mean score, having received mental health treatment in the past 12 months or not, and self-hate scale mean score; for social distancing intentions: age, gender [male, female, nonbinary], education level, and income level). Imputed data were analyzed using the *tidyverse* package in R [67]. Cohen *d* effect sizes and 95% CIs for analyses were calculated using *t* values for the treatment effect obtained from the analyses with the MOTE package in R [68]. We also conducted sensitivity analyses for all 3 hypotheses, in which all missing data for confirmatory outcomes were assumed to be in the 25th or 75th percentile of change in those outcomes observed in the sample. These analyses allowed us to examine the potential range of estimates for our hypotheses if we assumed the data were not missing at random but were instead impacted by unobserved confounders. See Multimedia Appendix 3 for the full imputation code and analytic strategy.

#### Testing Hypothesis 1

We tested whether the Contain COVID Anxiety SSI decreased scores on the GAD-7 immediately pre-SSI to 2 weeks later more than the Remain COVID Free SSI using a linear regression approach. We entered baseline GAD-7 score and condition as predictors of the follow-up GAD-7 mean score. We expected to see a larger decrease in GAD-7 score when the participants were randomized to Contain COVID Anxiety SSI compared with when they were randomized to the placebo Remain COVID Free. This pattern of differences would indicate a decrease in generalized anxiety disorder symptoms to a greater extent due to the Contain COVID Anxiety SSI compared with the Remain COVID Free SSI. We preregistered that a *P* value <.0167 (to Bonferroni correct for multiple comparisons) for condition in a linear model with a larger decrease in GAD-7 occurring when randomized to the Contain COVID Anxiety SSI would allow us to reject the null hypothesis that the difference between conditions was 0. We planned to confirm the pattern of differences by examining the sign of the condition coefficient and descriptive pattern of means based on condition. See Table 1 for all alternative interpretations of results.

#### Testing Hypothesis 2

We tested whether completing both interventions had an effect on social distancing intentions statistically equivalent to 0 using a paired-equivalence test. We entered baseline and postintervention social distancing intentions mean scores and SDs into a paired-equivalence test with equivalence bounds of *d*=–0.33 to *d*=0.33. We preregistered that a *P* value <.05 would allow us to reject the null hypothesis that the effect of completing both interventions was statistically different from 0. If the *P* value for this paired-equivalence test was nonsignificant, we preregistered that we would run a paired *t* test with baseline and postintervention social distancing intentions scores to determine if the association of the Contain COVID Anxiety intervention on social distancing intentions was significantly different from 0. We preregistered that if the *P* value was <.0167 (to Bonferroni correct for multiple comparisons), we would reject the null hypothesis that there was no difference pre to post within the active SSI intervention. See Table 1 for all alternative interpretations of results.

#### Testing Hypothesis 3

We tested whether the Contain COVID Anxiety SSI increased scores on the ACQ-EC more than the Remain COVID Free SSI using a linear regression approach. We entered baseline ACQ-EC scores and condition as predictors of immediate post-SSI ACQ-EC score. We expected to see a larger increase in ACQ-EC score when the participants were randomized to the Contain COVID Anxiety SSI compared with when they were randomized to the placebo Remain COVID Free SSI. This pattern of differences would indicate an increase in perceived control over anxiety to a greater extent due to the Contain COVID Anxiety SSI compared with the Remain COVID Free SSI. We preregistered that a *P* value <.0167 (to Bonferroni correct for multiple comparisons) for condition in a linear model with a larger increase in ACQ-EC occurring when randomized to the Contain COVID Anxiety SSI would allow us to reject the null hypothesis that the difference between conditions was 0. We planned to confirm the pattern of differences by examining the sign of the condition coefficient and descriptive pattern of means based on condition. See Table 1 for all alternative interpretations of results.

## Results

### Participant Demographics

Of the 529 participants who began the survey, 522 participants were randomized to achieve the weighted-probability sample of 500 (7 participants exited the survey prior to randomization, and 22 participants exited the survey prior to completion of the baseline survey; ie, 94.5% and 95.8% completion rates among individuals who started the baseline survey and among those who were randomized, respectively). All demographics for all randomized participants are reported by treatment condition in Table 2. Participants in both groups were experiencing, on average, mild anxiety (GAD-7 sum scores of 5.25-5.39), which were similar to the GAD-7 values assumed in our a priori power analysis (5.73). The sample appeared to be representative of the United States in terms of gender, age, and race/ethnicity. All responses were collected between September 13, 2020, and September 29, 2020.

**Table 2:** Demographics by treatment condition.

Demographics		Treatment received		
			Active Contain COVID Anxiety (n=261)	Placebo Remain COVID Free (n=261)
Age (years), mean (SD)		46.02 (15.65)		46.19 (15.71)
**Race/ethnicity, n (%)**				
	American Indian and/or Alaska Native		0 (0)	1 (0.4)
	Asian		19 (7.3)	16 (6.1)
	African American		31 (12.0)	39 (15.0)
	Hispanic or Latino/a		16 (6.1)	11 (4.2)
	Native Hawaiian or Pacific Islander		1 (0.4)	0 (0)
	White, non-Hispanic		187 (72.0)	190 (73.0)
	More than one race		4 (1.5)	3 (1.1)
	Other		3 (1.1)	1 (0.4)
**Gender, n (%)**				
	Agender		2 (0.8)	2 (0.8)
	Genderqueer or gender fluid		3 (1.1)	0 (0)
	Man		127 (49.0)	129 (49.0)
	Trans man		3 (1.1)	1 (0.4)
	Woman		125 (48.0)	129 (49.0)
	Other		1 (0.4)	0 (0)
**Sexual orientation, n (%)**				
	Asexual		6 (2.3)	5 (1.9)
	Bisexual		20 (7.7)	18 (6.9)
	Gay		3 (1.1)	7 (2.7)
	Heterosexual		218 (84.0)	220 (85.0)
	Lesbian		2 (0.8)	2 (0.8)
	Pansexual		3 (1.1)	6 (2.3)
	Queer		5 (1.9)	2 (0.8)
	Questioning or unsure		2 (0.8)	0 (0)
	Other		2 (0.8)	0 (0)
	Unknown		0 (0)	1 (0.4)
**Education, n (%)**				
	Less than high school degree		1 (0.4)	2 (0.8)
	High school degree		27 (10.0)	29 (11.0)
	Some college, no degree		74 (28.0)	67 (26.0)
	Associate degree		26 (10.0)	31 (12.0)
	Bachelor’s degree		77 (30.0)	99 (38.0)
	Master’s degree		46 (18.0)	24 (9.2)
	Professional degree		4 (1.5)	4 (1.5)
	Doctorate		6 (2.3)2	5 (1.9)
**Annual income (US $), n (%)**				
	Less than 10,000		17 (6.5)	14 (5.4)
	10,000-19,999		24 (9.2)	22 (8.4)
	20,000-29,999		29 (11.0)	28 (11.0)
	30,000-39,999		23 (8.8)	24 (9.2)
	40,000-49,999		21 (8.0)	33 (13.0)
	50,000-59,999		26 (10.0)	25 (9.6)
	60,000-69,999		15 (5.7)	24 (9.2)
	70,000-79,999		28 (11.0)	20 (7.7)
	80,000-89,999		15 (5.7)	10 (3.8)
	90,000-99,999		15 (5.7)	12 (4.6)
	100,000-149,999		27 (10.0)	30 (11.0)
	150,000 or more		21 (8.0)	19 (7.3)
**Relationship status, n (%)**				
	No current relationship		101 (39.0)	118 (45.0)
	Relationship, not living together		25 (9.6)	20 (7.7)
	Relationship, living together		24 (9.2)	22 (8.4)
	Engaged		3 (1.1)	3 (1.1)
	Married		108 (41.0)	98 (38.0)
Has children, n (%)		117 (45.0)		127 (49.0)
Health insurance covers mental health, n (%)		195 (75.0)		196 (75.0)
Received psychotherapy in the past year, n (%)		56 (21.0)		54 (21.0)
Received medication for mental health in the past year, n (%)		56 (21.0)		54 (21.0)
Perceived need for mental health treatment in the past year, n (%)		87 (33.0)		89 (34.0)
Baseline IDAS^a^-Dysphoria (1-5), mean (SD)		2.00 (0.90)		2.09 (0.92)
Baseline GAD-7^b^ (0-3), mean (SD)		0.75 (0.74)		0.77 (0.75)
Baseline self-hate (1-7), mean (SD)		2.07 (1.65)		2.20 (1.59)
Baseline perceived control over anxiety (0-5), mean (SD)		2.88 (1.34)		2.85 (1.29)
Baseline Hand Washing Intentions (1-7), mean (SD)		5.08 (1.59)		5.01 (1.61)
Baseline social distancing intentions of others (1-4), mean (SD)		2.27 (1.12)		2.32 (1.12)

^a^IDAS: Inventory of Depression and Anxiety Symptoms.

^b^GAD-7: Generalized Anxiety Disorder-7.

### Testing Participant Dropout

There was no evidence participants were significantly more likely to drop out of either condition at the 2-week follow-up (25/261, 9.6% dropped out from the Remain COVID Free SSI, and 18/261, 6.9% dropped out from the Contain COVID Anxiety SSI; *P*=.34). However, there was some evidence participants dropped out during the baseline survey significantly more often if they were randomized to the Contain COVID Anxiety SSI (20/261, 7.7% dropped out) versus the Remain COVID Free SSI (1/261, 0.4%; *P*<.001). Therefore, we interpreted the results for hypotheses 2 and 3 (which involve immediate postintervention outcomes) under conditions in which dropout is not presumed to be random (ie, a sensitivity test in which those who dropped out are assumed to change far more or less than average; see the preregistered sensitivity test in the publicly available code [52]). We also conducted this sensitivity test for Hypothesis 1, as unmeasured confounding can occur even if dropout does not significantly differ between conditions. All participants who were randomized were included in the intent-to-treat analyses (n=522).

### Testing Comprehension Questions

During our piloting of the Prolific platform (as outlined in our preregistered message), we noticed a substantial portion of participants were not answering the comprehension check questions correctly despite providing face-valid qualitative and quantitative data. We updated our comprehension check questions to attempt to align them more with completing the intervention with fidelity. However, among all participants who were randomized to the Contain COVID Anxiety SSI and answered a comprehension check question, 52.3% (126/241) answered both comprehension questions correctly. To examine whether this phenomenon was a function of the questions or lack of fidelity to the intervention, we developed a systematic qualitative coding system focused on fidelity for each qualitative response in the Contain COVID Anxiety SSI group. To be coded as having a high-fidelity qualitative response, the participant had to respond not only to the prompt with related content (a more general comprehension check) but also to the prompt as instructed (eg, a response enumerating concrete coping strategies to a prompt instructing participants to validate their own anxiety would be marked as a low fidelity response; see the publicly available code for the full qualitative coding system for fidelity check [52]).

We double-coded a random 20% of intervention responses (48 participants with 6 responses each, effective n=288) and found 87.13% average fidelity across these participants’ responses. Further, answering both comprehension check questions correctly shared only 0.01% of the variance with each participant’s fidelity score across their qualitative responses. We therefore determined that the comprehension check questions were poor indicators of completing the intervention with fidelity and chose to proceed with our planned analyses.

### Testing Hypothesis 1

In full intent-to-treat analyses with all participants who were randomized (n=522), we did not find support for the alternative hypothesis (*t*_520_=–0.71, *P*=.48; *d*=–0.06, 95% CI –0.27 to 0.15) and did find support for the null (noninferiority to placebo) hypothesis (*t*_520_=3.76, *P*<.001). These results were unchanged when we conducted a sensitivity test to determine whether results differed when participants who dropped out were assumed to have (1) experienced GAD-7 changes in the 25th percentile of the sample or (2) experienced GAD-7 changes in the 75th percentile of the sample (see publicly available code for the sensitivity tests for all hypotheses [52]). Therefore, we found evidence in favor of the placebo (Remain COVID Free SSI) being equally strong or stronger than the active condition (Contain COVID Anxiety SSI) in reducing generalized anxiety 2 weeks later. These results held when these tests were conducted in only the weighted-probability sample (n=500) and for only participants who answered both comprehension questions correctly (n=387). Within-group effect sizes indicated small but nonzero increases in generalized anxiety in both the Contain COVID Anxiety (*t*_260_=2.00; *d_z_*=0.12, 95% CI 0.002 to 0.25) and Remain COVID Free (*t*_260_=2.41; *d_z_*=0.15, 95% CI 0.03 to 0.27) groups.

### Testing Hypothesis 2

To make it possible to generate fully invertible matrices necessary to produce imputations, participant gender was dropped from the imputation model. In this case, the alternative hypothesis was operationalized as a change in social distancing intentions of others pre- to immediate post-Contain COVID Anxiety being statistically equivalent within a range of *d* of –0.33 to 0.33, while the null hypothesis was operationalized as a change in social distancing intentions in the same circumstance falling outside the effect range of *d* from –0.33 to 0.33. In full intent-to-treat analyses with all participants who were randomized to the Contain COVID Anxiety SSI (n=261), we found support for the alternative hypothesis (*t*_260_=4.63, *P*<.001) and did not find support for the null hypothesis (*t*_260_=0.70, *P*=.48; *d*=0.04, 95% CI –0.08 to 0.16). However, these results changed to unclear support for either the null or alternative hypothesis when we conducted a sensitivity test to determine whether results differed when participants who dropped out were assumed to have experienced (1) social distancing intentions of others changes in the 25th percentile of the sample or (2) social distancing intentions of others changes in the 75th percentile of the sample. Therefore, we found evidence that the participants in the Contain COVID Anxiety condition were statistically equivalent to participants in the Remain COVID Free condition in experiencing changes in social distancing intentions, though this result could be influenced by unmeasured confounding in participant dropout. These results held when these tests were conducted in only the weighted-probability sample (n=250) and in only participants who answered both comprehension questions correctly (n=126). See the publicly available code for the sensitivity analysis [52].

### Testing Hypothesis 3

In full intent-to-treat analyses with all participants who were randomized (n=522), we did not find support for the alternative hypothesis (*t*_520_=–0.21, *P*=.83; *d*=–0.02, 95% CI –0.23 to 0.19) and did find support for the null (noninferior to placebo) hypothesis (*t*_520_=2.40, *P*=.001). However, these results changed to unclear support for either the null or alternative hypothesis when we conducted a sensitivity test to determine whether results differed participants who dropped out were assumed to have experienced (1) ACQ-EC changes in the 25th percentile of the sample or (2) ACQ-EC changes in the 75th percentile of the sample. Therefore, we found evidence in favor of the placebo (Remain COVID Free) being equally strong or stronger than the active condition (Contain COVID Anxiety) in increasing perceived control over anxiety immediately postintervention, though this result could be influenced by unmeasured confounding in participant dropout. These results held when these tests were conducted in only the weighted-probability sample (n=500) and in only participants who answered both comprehension questions correctly (n=387). Within-group effect sizes were negligible in both the Contain COVID Anxiety (*t*_260_=1.03; *d_z_*=0.06, 95% CI –0.06 to 0.19) and Remain COVID Free (*t*_260_=1.63; *d_z_*=0.10, 95% CI –0.02 to 0.22). See the publicly available code for the sensitivity analysis [52].

## Discussion

### Principal Findings

Compared with a placebo control, a self-guided SSI for US adults did not improve short-term generalized anxiety or perceived control over anxiety during the COVID-19 pandemic. This high-powered randomized controlled trial (RCT), which used a nationally representative US sample, also demonstrated that this intervention did not worsen short-term generalized anxiety or perceived control. There was also statistically equivalent to zero iatrogenic movement within the intervention condition of beliefs in others’ willingness to social distance.

### Comparison With Prior Work

Interest in the use of brief, e-mental health interventions has increased substantially during the COVID-19 pandemic across the general adult population [69], and a large majority of these tools have minimal or no empirical support [70]. Even face-valid interventions containing evidence-based components may not necessarily improve mental health outcomes, and many mental health applications are used only once [71]. In this sample, a SSI for a community sample of adults, containing components associated with both proximal and longer-term mental health improvements in adolescents, did not lead to anxiety-related improvements above and beyond a placebo control. These differences could be due, at least in part, to sample characteristics: This study’s sample was older and more age-diverse than those for whom other self-guided SSIs have improved perceived control, anxiety, and depression [32,42,51], and participants were recruited from the broader US community rather than a clinically high-risk subgroup. Further, prior well-powered trials of SSIs targeting adults have significantly improved non-anxiety outcomes—such as positive psychotherapy expectancies [72] and positive parenting behaviors and distress tolerance in high-symptom individuals [73]—but self-guided SSI effects on clinical outcomes in adults outside of substance and alcohol use problems [37] have not been previously explored. It is also possible that the intervention tested by this study was simply not therapeutically effective, but that other interventions targeting similar outcomes in a similar sample may still be.

Accordingly, these results are the first to suggest that perceived control over anxiety and generalized anxiety symptoms may in fact be difficult to move in general adult samples via self-guided SSIs, at least in this nationally representative sample. Within-group effect sizes for perceived control over anxiety was negligible in both the active and placebo conditions, in contrast to within-group SSI effects seen in trials targeting adolescents. Further, nonzero increases were observed in generalized anxiety symptoms in both the active and placebo conditions over 2 weeks. Therefore, it is not the case that participants benefited from *either* condition (a placebo effect) but rather that they benefited from *neither* condition on targeted outcomes.

This design did not contain a wait list control condition, and we cannot explicitly rule out that receiving either light-touch intervention would have resulted in a smaller increase in generalized anxiety disorder symptoms compared with receiving nothing. This pattern of within-group effect sizes (ie, increasing generalized anxiety symptoms over time in both conditions) is consistent only with potentially preventative, as opposed to therapeutic, on average effects compared with “no treatment” control. Although we found no evidence of iatrogenic movement on social distancing intentions of others within our SSI, the lack of iatrogenic effects in other e-mental health interventions cannot be guaranteed without testing those outcomes directly. E-mental health applications hold promise in increasing mental health treatment access, [74] and well-powered tests of effectiveness must accompany (or ideally precede) dissemination if we wish to reduce overall mental health burden (eg, reducing subclinical anxiety symptoms) across general adult populations rather than solely the number of people without mental health support. Further, especially in the context of a pandemic, direct tests of iatrogenic outcomes should be included as primary outcomes in tests of single-session and light-touch mental health interventions.

We would like to propose 2 complementary paths toward building and understanding the impacts of effective SSIs for anxiety in adults, based on the results of this trial, which may generalize to evaluations of other light-touch interventions as well. First, given substantial heterogeneity in individual-level responses to any mental health intervention (including the SSI tested here), we recommend that researchers and program developers collect data necessary to build predictive models of individual-level response to SSIs. Predictive models require much larger sample sizes than typical clinical trials collect to identify subgroups of best responders. For example, recent simulation studies demonstrated that clinical trials may need as many as 500 participants per treatment arm to recover reliable predictions about who would benefit most from which treatment (ie, questions of moderation effects; [75])—far larger than typical mental health treatment RCTs (average n=52) [76]. Trials of self-guided SSIs create opportunities to quickly recruit large samples while retaining a rigorous experimental design. These larger sample sizes, combined with advances in feature engineering, could facilitate nuanced and definitive analyses regarding which individuals will (or will not) benefit from an extremely light-touch intervention. Such analyses could help situate self-guided SSIs within a stratified care system [77], where (for example) adults more and less likely to benefit from low-intensity support for anxiety are referred directly to the best-fit level of care.

Second, we recommend the systematic incorporation of qualitative and user-experience data into trials of self-guided SSIs. It has been posited that SSIs targeting adolescent mental health problems may show acceptability and efficacy, at least in part, because they do not “feel” like interventions to youth—that is, they are designed to be nonstigmatizing to users [39]. However, systematic qualitative data around participants’ experiences with SSIs for mental health are scarce, and this hypothesis has not been systematically tested. Collecting and analyzing qualitative and user experience data could clarify how people view self-guided SSIs as similar or different to longer-term and face-to-face interventions and whether these perceptions differ across distinct populations (eg, youth versus adults, given that many elements of youth-directed SSI design were developed through a developmentally specific lens). User experience data may be analyzed using both qualitative (eg, grounded theory) and quantitative (eg, topic modeling) methods to leverage this important information as much as possible during iterative intervention development.

### Limitations

There are certainly limitations to what this study can conclude. First, this study was conducted during the COVID-19 pandemic, and it is unclear whether the nonzero increases in generalized anxiety within both groups reflected the many structural challenges of pandemic conditions (which a self-guided SSI cannot change) or would have occurred regardless. Examination of within-group effect sizes in self-guided SSI trials conducted after the COVID-19 pandemic ends should examine whether negligible to slightly increasing within-group effect sizes persist for clinical anxiety in unselected adult samples. Other work suggests that certain outcomes, such as parenting behaviors and distress tolerance, may be modifiable via self-guided SSIs in high-symptom adults even during the pandemic [73]. Second, our original quality check measure—3 multiple-choice comprehension check items, created specifically for this trial—proved invalid as a gauge of intervention fidelity, sharing only 0.01% of variance with a subsequently developed, more rigorous, qualitatively coded intervention fidelity metric. This improved qualitatively coded fidelity measure showed that participants were highly successful in completing the interventions as intended, per their written responses to within-program prompts. Thus, it is unlikely the null results are due to the lack of participants engaging with and understanding the intervention content. It is also possible that the study’s sample, while representative of the US public across demographic variables, was subject to selection bias owing to their participation in Prolific. Additionally, should these SSIs be disseminated outside of an RCT context (ie, not posted as a paid research opportunity on Prolific), it is possible that a different pattern of results may emerge. Finally, although we did not observe differential dropout for our primary outcome 2 weeks later, there was higher dropout in the intervention group than in the placebo group during the baseline session containing the interventions. This pattern fits with sensitivity tests indicating that, if dropout did not occur at random, our statistical conclusions about perceived control over anxiety and the social distancing intentions of others become unclear. However, across all other sensitivity analyses, we found support for the null hypotheses, and within-group effect sizes would remain negligible regardless of dropout across conditions. Finally, this study was conducted in a US context, and its results cannot be assumed to generalize to other countries.

### Conclusions

Compared with a placebo control, an 8-minute, self-guided SSI for US adults did not improve short-term generalized anxiety nor perceived control over anxiety during the COVID-19 pandemic. Additionally, neither condition yielded any iatrogenic movement in a key public health behavior (assumed social distancing intentions of others). Our rigorous methods and well-powered sample bolster confidence in these results, which carry direct implications for future research on self-guided SSIs for mental health problems—both for anxiety in adults and more broadly. As with so many interventions targeting complex, individual-level problems, key questions for SSI research remain: “which intervention, for whom, and under what circumstances?” [8].

## Appendix

Multimedia Appendix 1 Full survey and interventions.

Multimedia Appendix 2 CONSORT-eHEALTH checklist (V 1.6.1).

Multimedia Appendix 3 COVID Anxiety SSI RR preprocessing and analyses.

## Abbreviations

ACQ-EC: Anxiety Control Questionnaire-Emotion Control
CDC: Centers for Disease Control and Prevention
GAD-7: Generalized Anxiety Disorder-7
IRB: institutional review board
RCT: randomized controlled trial
SSI: single-session intervention

## Data Availability

Data from this study are publicly available [52].
