# Supplementary material for: Web-Based Single Session Intervention for Perceived Control Over Anxiety During COVID-19: Randomized Controlled Trial
Source: JMIR Ment Health. 2022 Apr 12;9(4):e33473. doi: 10.2196/33473 (PMC9007232; doi:10.2196/33473)
Supplement: Multimedia Appendix 1 [file mental_v9i4e33473_app1.docx]

Multimedia Appendix 1. Full Survey and Interventions

**Between_Sub_Baseline_COVID_19_SSI**

**Start of Block: Demographics**

Q3

We selected these specific questions because previous research has shown they'll help us get a snapshot of who you are and how you've been feeling recently.

We know we won't capture everything that makes you who you are, but we appreciate you giving us a glimpse into your life.

You'll spend about 10 minutes answering these questions.

After that you'll be giving us important feedback on our materials. We need your advice for how to best communicate these ideas to other people.

Q108 Timing

First Click (1)

Last Click (2)

Page Submit (3)

Click Count (4)

| Page Break |  |
| --- | --- |

Q5

This set of questions will help us get to know you a bit better!

This is also the longest page of questions, and after that there's only 3 more (shorter) pages until you help us with our materials.

|  |
| --- |

prolific_id Please confirm your Prolific ID

________________________________________________________________

|  |
| --- |

age How old are you? (Please type a number, like 34)

________________________________________________________________

|  |  |
| --- | --- |

race_eth How do you usually describe yourself? (please choose the most applicable)

o American Indian and/or Alaska Native (1)

o Asian (including Asian Desi) (2)

o Black or African American (3)

o Hispanic or Latino/a or Latinx (4)

o Native Hawaiian or other Pacific Islander (5)

o White, non-Hispanic (includes Middle Eastern) (6)

o More than one race (7)

o Other, please specify (8) ________________________________________________

|  |
| --- |

gender With which gender(s) do you identify?

o Agender (1)

o Androgyne (2)

o Demigender (3)

o Genderqueer or gender fluid (4)

o Man (5)

o Questioning or unsure (6)

o Trans man (7)

o Trans woman (8)

o Woman (9)

o Additional gender category/identity or a combination of listed options: please specify (10) ________________________________________________

|  |
| --- |

sexual_orientation What is your sexual orientation? (select all that apply - optional)

o Asexual (1)

o Bisexual (2)

o Gay (3)

o Straight (heterosexual) (4)

o Lesbian (5)

o Pansexual (6)

o Queer (7)

o Questioning or unsure (8)

o Same-gender loving (9)

o Additional sexual orientation or a combination of listed options: please specify (10) ________________________________________________

|  |
| --- |

edu_level What is the highest degree or level of school you have completed? (If you’re currently enrolled in school, please indicate the highest degree you have *received.*)

o Less than a high school diploma (1)

o High school degree or equivalent (e.g. GED) (2)

o Some college, no degree (3)

o Associate degree (e.g. AA, AS) (4)

o Bachelor’s degree (e.g. BA, BS) (5)

o Master’s degree (e.g. MA, MS, MEd) (6)

o Professional degree (e.g. MD, DDS, DVM) (7)

o Doctorate (e.g. PhD, EdD) (8)

|  |
| --- |

zip_code_first_3 What are the first 3 digits of your zip code? For example, if your zip code was 78712 you would type "787"

We have discussed asking this question with Prolific and by Health and Human Services standards you cannot be re-identified based on providing only the first 3 digits of your zip code. (See https://www.hhs.gov/hipaa/for-professionals/privacy/special-topics/de-identification/index.html#zip for more information)

We're asking this question to better understand how COVID-19 is currently impacting your general community. You are also free to skip this question if you do not want to answer it.

________________________________________________________________

|  |
| --- |

income Information about income is very important to understand. Would you please give your best guess?Please indicate the answer that includes your entire household income in 2019 before taxes.

o Less than $10,000 (1)

o $10,000 to $19,999 (2)

o $20,000 to $29,999 (3)

o $30,000 to $39,999 (4)

o $40,000 to $49,999 (5)

o $50,000 to $59,999 (6)

o $60,000 to $69,999 (7)

o $70,000 to $79,999 (8)

o $80,000 to $89,999 (9)

o $90,000 to $99,999 (10)

o $100,000 to $149,999 (11)

o $150,000 or more (12)

|  |  |
| --- | --- |

relationship Which of the following best describes your relationship status?

o Not currently in a romantic relationship (1)

o In a romantic relationship but not living together (2)

o In a romantic relationship and living together (3)

o Engaged (4)

o Married (5)

|  |  |
| --- | --- |

children Do you have children?

o Yes (1)

o No (0)

|  |
| --- |

insurance_mh In the past 12 months, did you have health insurance that covers (helps pay for) mental health care like therapy or counseling?

o Yes (1)

o No (0)

|  |
| --- |

rec_therapy In the past 12 months, did you receive therapy from a professional counselor or therapist to help you with emotional or mental health problems such as feeling sad, blue, anxious, or nervous?

o Yes (1)

o No (0)

|  |
| --- |

rec_medication In the past 12 months, did a healthcare professional prescribe medications to help you with emotional or mental health problems such as feeling sad, blue, anxious, or nervous?

o Yes (1)

o No (0)

|  |
| --- |

perceived_need In the past 12 months, did you think you needed help for emotional or mental health problems such as feeling sad, blue, anxious, or nervous?

o Yes (1)

o No (0)

ideology Here is a 7-point scale on which the political views that people might hold are arranged from extremely liberal (1) to extremely conservative (7). Where would you place yourself on this scale?

o 1 - Extremely Liberal (1)

o 2 - Liberal (2)

o 3- Slightly Liberal (3)

o 4 - Moderate, Middle of the Road (4)

o 5 - Slightly Conservative (5)

o 6 - Conservative (6)

o 7 - Extremely Conservative (7)

demo_page_1_tim Timing

First Click (1)

Last Click (2)

Page Submit (3)

Click Count (4)

| Page Break |  |
| --- | --- |

|  |
| --- |

covid_sick In the past 30 days, have you been sick for more than one day with an illness that included any of the following: fever, cough, sore throat, or runny or stuffy nose?

o Yes (1)

o No (0)

*Display This Question:*

*If In the past 30 days, have you been sick for more than one day with an illness that included any o... = Yes*

|  |
| --- |

covid_symptoms Which of the following symptoms did you have?

▢ A fever/feverish (1)

▢ Cough (2)

▢ Sore throat (3)

▢ Runny or stuffy nose (4)

▢ Difficulty breathing (5)

*Display This Question:*

*If In the past 30 days, have you been sick for more than one day with an illness that included any o... = Yes*

|  |
| --- |

covid_test_or_not For this illness, were you tested for novel coronavirus (COVID-19)?

o Yes (1)

o No (0)

*Display This Question:*

*If For this illness, were you tested for novel coronavirus (COVID-19)? = Yes*

|  |
| --- |

covid_test_positive Was the test for novel coronavirus positive?

o Yes (1)

o No (0)

o Unknown (2)

*Display This Question:*

*If Do you have children? = Yes*

|  |
| --- |

schools_closed Are your children's schools' physical locations currently closed?

o Yes (1)

o No (0)

|  |
| --- |

c19_impacts How has the COVID-19 outbreak affected you in the past two weeks?

▢ Worked remotely or from home more than you usually do (1)

▢ Worked more hours than usual (2)

▢ Worked reduced hours (3)

▢ Was not able to work (4)

▢ Had difficulty arranging for childcare (5)

▢ Incurred increased costs for childcare expenses (6)

▢ Income or pay has been reduced (7)

▢ Not paid at all (8)

▢ Had serious financial problems (9)

demo_page_2_tim Timing

First Click (1)

Last Click (2)

Page Submit (3)

Click Count (4)

| Page Break |  |
| --- | --- |

|  |
| --- |

b_idas_dysphoria On this page is a list of feelings, sensations, problems and experiences that people sometimes have. Read these statements to determine how well they describe your recent feelings and experiences.

Then select the option that best describes how much you have felt or experienced things this way during the past TWO WEEKS, including today.

|  | Not at all (1) | A little bit (2) | Moderately (3) | Quite a bit (4) | Extremely (5) |
| --- | --- | --- | --- | --- | --- |
| I felt depressed (1) | o | o | o | o | o |
| I felt inadequate (2) | o | o | o | o | o |
| I felt fidgety, restless (3) | o | o | o | o | o |
| I blamed myself for things (4) | o | o | o | o | o |
| I felt discouraged about things (5) | o | o | o | o | o |
| I had little interest in my usual hobbies or activities (6) | o | o | o | o | o |
| I had trouble concentrating (7) | o | o | o | o | o |
| I had trouble making up my mind (8) | o | o | o | o | o |
| I talked more slowly than usual (9) | o | o | o | o | o |
| I found myself worrying all the time (10) | o | o | o | o | o |

|  |
| --- |

b_gad_7 Over the last 2 weeks, how often have you been bothered by the following problems?

|  | Not at all (0) | Several days (1) | More than half the days (2) | Nearly every day (3) |
| --- | --- | --- | --- | --- |
| Feeling nervous, anxious, or on edge (1) | o | o | o | o |
| Not being able to stop or control worrying (2) | o | o | o | o |
| Worrying too much about different things (3) | o | o | o | o |
| Trouble relaxing (4) | o | o | o | o |
| Being so restless that it's hard to sit still (5) | o | o | o | o |
| Becoming easily annoyed or irritable (6) | o | o | o | o |
| Feeling afraid as if something awful might happen (7) | o | o | o | o |

|  |
| --- |

b_self_hate The following questions ask you to think about **your own beliefs and experiences**. Please base your

responses on how you are feeling **right now**. There are no right or wrong answers, we are interested in what **you** think and feel.

|  | Not at all true for me  1 (1) | 2 (2) | 3 (3) | Somewhat true for me  4 (4) | 5 (5) | 6 (6) | Very true for me  7 (7) |
| --- | --- | --- | --- | --- | --- | --- | --- |
| I hate myself (1) | o | o | o | o | o | o | o |
| I am a failure (2) | o | o | o | o | o | o | o |
| I feel disgusted when I think about myself (3) | o | o | o | o | o | o | o |
| I am ashamed of myself (4) | o | o | o | o | o | o | o |
| I have no value (5) | o | o | o | o | o | o | o |
| I wish I could escape from myself (6) | o | o | o | o | o | o | o |
| I am not proud of myself (7) | o | o | o | o | o | o | o |

b_mh_symp_tim Timing

First Click (1)

Last Click (2)

Page Submit (3)

Click Count (4)

**End of Block: Demographics**

**Start of Block: Pre Intervention**

|  |
| --- |

b_acq Listed below are a number of statements describing a set of beliefs. Please read each statement carefully and, on the 0-5 scale given, indicate how much you think each statement is true about you **right now**.

|  | 0 - Strongly Disagree (0) | 1 - Moderately Disagree (1) | 2 - Slightly Disagree (2) | 3 - Slightly Agree (3) | 4 - Moderately Agree (4) | 5 - Strongly Agree (5) |
| --- | --- | --- | --- | --- | --- | --- |
| I am unconcerned if I become anxious in a difficult situation, because I am confident in my ability to cope with my symptoms (1) | o | o | o | o | o | o |
| I can relax when I want (2) | o | o | o | o | o | o |
| I can put worrisome thoughts out of my mind easily (3) | o | o | o | o | o | o |
| I am able to control my level of anxiety (4) | o | o | o | o | o | o |

b_soc_dist Starting today, for how long do you believe others would be willing to engage in the following behaviors?

|  | Less than a month (1) | 1 month (2) | 2-3 months (3) | 4 months or more (4) |
| --- | --- | --- | --- | --- |
| Avoid going out to a restaurant, bar, or club (1) | o | o | o | o |
| Avoid going to a family gathering like a birthday party or wedding or funeral (2) | o | o | o | o |
| Avoid going to a social gathering with friends, peers, or coworkers (not including relatives) (3) | o | o | o | o |

|  |
| --- |

b_hand_wash_int

|  | Strongly Agree (7) | (6) | (5) | (4) | (3) | (2) | Strongly Disagree (1) |
| --- | --- | --- | --- | --- | --- | --- | --- |
| In the future, I intend to wash my hands at least 10 times a day (1) | o | o | o | o | o | o | o |
| In the future, I intend to wash my hands more often (2) | o | o | o | o | o | o | o |
| In the future, I intend to wash my hands as often as possible (3) | o | o | o | o | o | o | o |

**End of Block: Pre Intervention**

**Start of Block: COVID-19 Hand Washing SSI**

c19_hw_slide_1

**Welcome!** We are psychologists from universities around the world. Among other things, we study behavior and how to improve our habits.

Right now we all need to improve our habits around hand-washing. COVID-19 is classified by the World Health Organization as a pandemic, and **one of the few ways to help slow its spread is to frequently and thoroughly wash our hands.**

So, we want to give away our best advice for improving hand-washing behavior during COVID-19, based on science and experience working with patients around the world...

c19_hw_slide_1_tim Timing

First Click (1)

Last Click (2)

Page Submit (3)

Click Count (4)

| Page Break |  |
| --- | --- |

c19_hw_slide_2 **What will I be doing?**

In this **5-minute activity**, we will share:

1. The **science** behind why hand-washing is so important

2. **Advice** from people around the world for how they’ve improved hand-washing habits

3. An **opportunity** to see action plans for improving hand-washing habits.

c19_hw_slide_2_tim Timing

First Click (1)

Last Click (2)

Page Submit (3)

Click Count (4)

| Page Break |  |
| --- | --- |

c19_hw_slide_3 **What Does the Science Say About Hand-Washing?** A lot of people ask us whether hand-washing is actually that important. Lots of people didn’t wash their hands very much before the pandemic—and they didn’t get sick all the time.

**So, do my personal hand-washing habits *really* make a difference?**

**Scientifically, *yes*. Hand-washing is one of the only things that is often under our control that can majorly slow the spread of COVID-19!**

c19_hw_slide_3_tim Timing

First Click (1)

Last Click (2)

Page Submit (3)

Click Count (4)

| Page Break |  |
| --- | --- |

c19_hw_slide_4 **What Does the Science Say About Hand-Washing?**

**Soap** does two things really well: It tightly clings to the shells of viruses and water at the same time. Soap uses this unique “clinginess” to **pull apart the COVID-19 virus**, like a crowbar, **leaving it unable to infect you.** This gif shows how soap does this!

Citations: World Health Organization https://tinyurl.com/vox-soap-gif

c19_hw_slide_4_tim Timing

First Click (1)

Last Click (2)

Page Submit (3)

Click Count (4)

| Page Break |  |
| --- | --- |

c19_hw_slide_5 **We Also Need to Wash Our Hands Effectively Enough to Slow the Virus!** We need to give soap a long enough time to do its job! Just washing for a couple of seconds doesn’t give the soap long enough to pry the virus apart, which is why the World Health Organization recommends we wash our hands for at least 20 seconds (Or the time it takes to sing happy birthday twice)

Also, according to a study from the British Medical Journal, we should all wash our hands at least 10 times a day during pandemic circumstances.

Luckily, 85% of people wash their hands right after using restrooms. But we need to do better to keep ourselves safe during COVID-19

(Especially because people are pretty bad at not touching their faces. Most of us touch our face around 23 times an hour, even without realizing it!)

Citations: British Medical Journal CDC American Journal of Infection Control

c19_hw_slide_5_tim Timing

First Click (1)

Last Click (2)

Page Submit (3)

Click Count (4)

| Page Break |  |
| --- | --- |

c19_hw_slide_6 **Ok, But How Can I Actually Wash My Hands More Effectively?**

We’ve talked to people around the world and figured out a simple,

**3-step plan for better hand-washing habits:**

1. **Write down specific times** you know you need to wash your hands. 2. **Set reminders in your calendar** or **alarms on your phone** to make sure you **wash your hands at least 10 times each day.** 3. Sing **happy birthday twice** to your favorite celebrity **while washing your hands**

c19_hw_slide_6_tim Timing

First Click (1)

Last Click (2)

Page Submit (3)

Click Count (4)

| Page Break |  |
| --- | --- |

c19_hw_slide_7 **What Has Worked for Others** Here are 3 examples of what’s **worked for other people** after they learned how to get better at hand-washing. MK (we’re using fake initials to keep their information confidential) made this plan: **Step 1:** For my 'specific times', I decided to just wash my hands every hour on the hour to make it easier to remember. It was also more often in between meetings so it was easier to excuse myself

**Step 2:** I really liked the calendar reminders idea so I didn’t have my phone going off at weird times

**Step 3:** I decided to sing happy birthday to Oprah because I thought it would make me happy Later, MK said: “At first I thought I wouldn’t be able to keep up with this pace, but I’ve gotten into a pretty good groove. It’s wild how much more I’m washing my hands now than I used to.”

c19_hw_slide_7_tim Timing

First Click (1)

Last Click (2)

Page Submit (3)

Click Count (4)

| Page Break |  |
| --- | --- |

c19_hw_slide_8 **What Has Worked for Others, Part 2** GS shared this plan with us:

**Step 1:** For my 'specific times', I tried to schedule mine in between the lessons I’m teaching my kids who I’m quarantined with right now

**Step 2:** I had to use my phone, otherwise I never would have heard the reminders!

**Step 3:** I decided to sing happy birthday to Batman out loud because it made my kids laugh when I did it

We also talked to them about our materials, and GS said:

“Taking some time to do this really helped me feel like I was being a lot more hygenic. I noticed that I also started to wash my hands more frequently at appropriate times, like before preparing food, since I was already in the habit.”

c19_hw_slide_8_tim Timing

First Click (1)

Last Click (2)

Page Submit (3)

Click Count (4)

| Page Break |  |
| --- | --- |

c19_hw_slide_9 **What Has Worked For Others, Part 3** CG told us:

**Step 1:** For my 'specific times', I had to schedule them at pretty random times since I’m trying to help take care of family members

**Step 2:** I used a combination of my phone and calendar, since I’m more likely to be paying attention to one or the other at different times of day

**Step 3:** I decided to sing Happy Birthday to my grandfather since he’s a celebrity to me

After implementing their plan, CG said:

“I’m glad I’m actually washing my hands more often. I thought I was doing ok before but seeing how much more often I’m washing now I realize how wrong I was!”

c19_hw_slide_9_tim Timing

First Click (1)

Last Click (2)

Page Submit (3)

Click Count (4)

| Page Break |  |
| --- | --- |

c19_hw_slide_10 **Now You Can Use These Action Plans To Help Yourself** Using these people’s experiences as a guide, you can wash your hands more effectively. Just following these steps can boost your hand-washing—and protect yourself and others during this pandemic.

c19_hw_slide_10_tim Timing

First Click (1)

Last Click (2)

Page Submit (3)

Click Count (4)

| Page Break |  |
| --- | --- |

c19_hw_slide_11 **Here's a Reminder!**

Here’s the 3 steps you can apply to more effectively wash your hands in general: 1. **Write down specific times** you know you need to wash your hands.

2. **Set** **reminders in your calendar** or **alarms on your phone** to make sure you **wash your hands at least 10 times each day.**

3. **Sing happy birthday twice** to your favorite celebrity **while washing your hands**

We would also appreciate you answering the questions on the following page so we can understand how our program is impacting people (We want to change it to make it even better!)

c19_hw_slide_11_tim Timing

First Click (1)

Last Click (2)

Page Submit (3)

Click Count (4)

**End of Block: COVID-19 Hand Washing SSI**

**Start of Block: Post Intervention**

*Display This Question:*

*If cond = 1*

c19_anx_comp_1_t1 As part of step 1 the 3-step coping process with COVID-19 related anxiety, you write about:

o Only things you can't control (1)

o Only things you can control (2)

o Both things you can and can't control (3)

o None of these are part of the 3-step coping process for COVID-19 related anxiety (4)

*Display This Question:*

*If cond = 1*

c19_anx_comp_2_t1 According to the 3-step coping process for COVID-19 related anxiety, what do you do in step 2 of the coping process?

o Choose only one thing you can control to focus on (1)

o Choose at least two things you can control to focus on (2)

o Choose only one thing you can't control to focus on (3)

o Choose at least two things you can't control to focus on (4)

*Display This Question:*

*If cond = 0*

c19_hw_comp_1_t1 As part of step 3 of the 3-step process for more effectively washing your hands during COVID-19, you write about:

o Which song you will sing along to make sure you wash your hands for long enough (1)

o Which celebrity you will sing happy birthday to twice while you wash your hands (2)

o A list of reasons why you should wash your hands (3)

o None of these are part of the 3-step coping process for more effectively washing your hands during COVID-19 (4)

*Display This Question:*

*If cond = 0*

c19_hw_comp_2_t1 According to the 3-step process for more effectively washing your hands during COVID-19, what do you do in step 2 of the coping process?

o Choose which kind of reminders (alarms on cell phone or calendar reminders) you will use to help yourself remember to wash your hands (1)

o Choose how many reminders you will set to help yourself remember to wash your hands (2)

o Choose how often you will wash your hands (3)

o Choose how long you will wash your hands (4)

|  |
| --- |

pi_acq Listed below are a number of statements describing a set of beliefs. Please read each statement carefully and, on the 0-5 scale given, indicate how much you think each statement is true about you **right now**.

|  | 0 - Strongly Disagree (0) | 1 - Moderately Disagree (1) | 2 - Slightly Disagree (2) | 3 - Slightly Agree (3) | 4 - Moderately Agree (4) | 5 - Strongly Agree (5) |
| --- | --- | --- | --- | --- | --- | --- |
| I am unconcerned if I become anxious in a difficult situation, because I am confident in my ability to cope with my symptoms (1) | o | o | o | o | o | o |
| I can relax when I want (2) | o | o | o | o | o | o |
| I can usually put worrisome thoughts out of my mind easily (3) | o | o | o | o | o | o |
| I am able to control my level of anxiety (4) | o | o | o | o | o | o |

pi_soc_dist Starting today, for how long do you believe others would be willing to engage in the following behaviors?

|  | Less than a month (1) | 1 month (2) | 2-3 months (3) | 4 months or more (4) |
| --- | --- | --- | --- | --- |
| Avoid going out to a restaurant, bar, or club (1) | o | o | o | o |
| Avoid going to a family gathering like a birthday party or wedding or funeral (2) | o | o | o | o |
| Avoid going to a social gathering with friends, peers, or coworkers (not including relatives) (3) | o | o | o | o |

|  |
| --- |

pi_hand_wash_int

|  | Strongly Agree (7) | (6) | (5) | (4) | (3) | (2) | Strongly Disagree (1) |
| --- | --- | --- | --- | --- | --- | --- | --- |
| In the future, I intend to wash my hands at least 10 times a day (1) | o | o | o | o | o | o | o |
| In the future, I intend to wash my hands more often (2) | o | o | o | o | o | o | o |
| In the future, I intend to wash my hands as often as possible (3) | o | o | o | o | o | o | o |

**End of Block: Post Intervention**

**Start of Block: COVID-19 Anxiety SSI**

c19_anx_slide_1

**We need your help!** We are psychologists from universities around the world. We study the brain, anxiety, and how to cope when things are hard. ...And right now, things are **hard**. In fact, we’re sharing this activity while quarantined ourselves, after being exposed to the COVID-19 virus. We’re doing our best to help loved ones and patients while caring for ourselves, too.

So, we want to give away our best advice for handling anxiety around COVID-19, based on **science** and **experience** working with patients around the world...

c19_anx_slide_1_tim Timing

First Click (1)

Last Click (2)

Page Submit (3)

Click Count (4)

| Page Break |  |
| --- | --- |

c19_anx_slide_2

**What Will I Be Doing?** In this **5-minute activity**, we will share:

1. The **science** behind why it’s normal to feel anxious right now

2. **Advice** from people around the world for how they’ve coped

3. An **opportunity** for you to pay it forward, and give others your best advice for coping right now.

**We’re all in this together.** Someone out there needs the advice that only you can give.

c19_anx_slide_2_tim Timing

First Click (1)

Last Click (2)

Page Submit (3)

Click Count (4)

| Page Break |  |
| --- | --- |

c19_anx_slide_3 **What Does the Science Say About COVID-19 Anxiety?** A lot of people ask us why anxiety even exists. Worrying can make it hard to even think about work or school. So, **does worry have any real purpose**?

**Scientifically, yes. In fact, anxiety has been *necessary* to human survival!**

Early humans developed “anxiety,” or fear, as a **tool** to help us **avoid danger** in our environments. (Think wooly mammoths and saber-toothed tigers). People who were better at avoiding danger lived longer and better lives. Our anxiety response is part of what **makes us human and helps keep us alive**.

Citation: Ethnology and Sociobiology

c19_anx_slide_3_tim Timing

First Click (1)

Last Click (2)

Page Submit (3)

Click Count (4)

| Page Break |  |
| --- | --- |

c19_anx_slide_4 **Recent Scientific Findings Help Explain Why Being Anxious is Normal Right Now** In fact, recent computational neuroscience findings show the inferior frontal gyrus (IFG) in our brains (the red highlighted areas in the picture below) might help us figure out *when* to get anxious enough to avoid dangerous situations. **Being at least a little anxious** at least some of the time right now means **your brain is doing its job.**

Citation: Frontiers in Systems Neuroscience

https://tinyurl.com/ifg-scan

c19_anx_slide_4_tim Timing

First Click (1)

Last Click (2)

Page Submit (3)

Click Count (4)

| Page Break |  |
| --- | --- |

c19_anx_slide_5 **In Other Words Feeling Anxiety In Response to COVID-19 is a Scientifically Typical, Adaptive Response!** When they were asked in private, **up to 78% of people admitted to feeling anxious about COVID-19**. So you’re not nearly as alone in your anxiety as you might feel.

It can be hard to believe this when we see others sending e-mails like nothing is happening, friends posting jokes on social media, or media reports of people seeming to continue their everyday lives. But the truth is **a vast majority of people are worried**, and the number of people who aren’t worried is shrinking by the day.

Citation: Elon University National Poll, March 18. 2020

c19_anx_slide_5_tim Timing

First Click (1)

Last Click (2)

Page Submit (3)

Click Count (4)

| Page Break |  |
| --- | --- |

c19_anx_slide_6 **Ok, But What Do I Do About My Anxiety?** Feeling less alone in our anxiety can be helpful all by itself, but we can understand people want an easy-to-use plan to help feel better.

This can be especially true for people whose anxiety is no longer helping them engage in adaptive behaviors (washing hands more frequently, reducing contact with people in high risk groups) and instead just making them feel miserable.

We’ve talked to people around the world and figured out **the simplest way to feel more in control of your anxiety is following this 3 step plan:**

1. **Remind yourself feeling anxious and stressed right now is totally normal.** Then write down some parts of your current stress **you can’t control** and what parts **you can control**.

2. From the list of things **you can control, pick one thing** that bothers you a lot.

3. Figure out **one small step** you can take to help yourself with that **one thing** you can control (Repeat these 3 steps as many times as necessary)

c19_anx_slide_6_tim Timing

First Click (1)

Last Click (2)

Page Submit (3)

Click Count (4)

| Page Break |  |
| --- | --- |

c19_anx_slide_7 **What Has Worked for Others** Here are 3 examples of what’s **worked for other people** after they learned the science behind COVID-19 anxiety.

JH (we’re using fake initials to keep their information confidential) made this plan: **Step 1:** I reminded myself, "It’s understandable I’m freaking out since a global pandemic is literally happening right now on top of all of my regular stress"

What I Can’t Control: How fast the virus spreads, Feeling unfocused What I Can Control: Helping others I know, How I respond to feeling socially disconnected

**Step 2:** I picked changing, 'how I respond to feeling socially disconnected'

**Step 3:** My 'one small step' was reaching out to a few friends to set up Zoom hangouts Later, JH said: “Taking this simple first step helped re-connect me with people I hadn’t been able to hang out with because of physical distance. I’m not pretending everything is suddenly wonderful, but now I feel generally **more confident I can handle my stress and anxiety** when it comes up.”

c19_anx_slide_7_tim Timing

First Click (1)

Last Click (2)

Page Submit (3)

Click Count (4)

| Page Break |  |
| --- | --- |

c19_anx_slide_8 **What Has Worked For Others, Part 2** PB shared this plan with us: **Step 1:** I reminded myself, "Anyone who lost their job during all of this would be having a hard time, so it’s not like I’m messing up"

What I Can’t Control: When the restaurant I work at re-opens, How fast my thoughts are moving What I Can Control: Staying in touch with people who might hire me in the future, How I handle my thoughts (working out vs. thinking over and over)

**Step 2:** I picked, 'staying in touch with people who might hire me in the future'

**Step 3:** My 'one small step' was reaching out to multiple old bosses who know my work to check in on them and get leads When talking about our materials, PB said: “I kind of thought materials like this might be BS before I started, but it was nice to focus on what I can control rather than all the things I can’t. I wish it were better, and I’m still glad I’m doing what I can by finding little ways to change what I do. Once I took the first step the other steps got easier way quicker than I would have thought.”

c19_anx_slide_8_tim Timing

First Click (1)

Last Click (2)

Page Submit (3)

Click Count (4)

| Page Break |  |
| --- | --- |

c19_anx_slide_9 **What Has Worked for Others, Part 3** ZL told us: **Step 1:** I reminded myself, "There’s a reason we don’t normally expect people to teach their children, work a full-time job, and provide support to other people. Of course I’m anxious!"

What I Can’t Control: Getting annoyed more often around my kids, The safety of my far-away relatives What I Can Control: Getting more sleep (at least sometimes), How often I check the news

**Step 2:** I picked, 'Getting more sleep (at least sometimes)'

**Step 3:** My 'one small step' was giving myself a bedtime routine and get realistic about how early I have to be up with the kids being at home more often After implementing their plan, ZL said: “When I started quarantining with my kids I thought I was going to lose it. I love them, but trying to provide them with attention and education in addition to everything else left me emotionally strung out. These materials helped me figure out I didn’t have to choose the 'perfect' thing to start working on to feel better. Making one small change to start was a way more realistic way to start making my life easier.”

c19_anx_slide_9_tim Timing

First Click (1)

Last Click (2)

Page Submit (3)

Click Count (4)

| Page Break |  |
| --- | --- |

c19_anx_slide_10 **Now We Need Your Action Plan for Helping Yourself and Others** Using these people’s experiences as a guide, please create an action plan that may be helpful to you.

**With your permission, we’ll use your anonymous plan as one of many examples, to help others around the world.** Once we collect many people’s plans, we will post them online for anyone to see.

Along with helping others, making a plan might help you **figure out what you CAN do to get through this stressful time.**

We don't care about spelling, grammar, or anything like that. We just want your insights and advice so we can work together to help other people.

You'll start making your plan on the next page, with one page per step so you don't have to do a bunch of scrolling. Once you finish your action plan we’ll give you a copy of the three steps and the action plan you end up writing for yourself so you can save it for later.

c19_anx_slide_10_tim Timing

First Click (1)

Last Click (2)

Page Submit (3)

Click Count (4)

| Page Break |  |
| --- | --- |

c19_anx_normal_qual

**Step 1**

Please consider sharing a way to remind yourself it's understandable and scientifically normal to be anxious right now (~1 sentence)

(Example: It’s understandable I’m freaking out since a global pandemic is literally happening right now on top of all of my regular stress)

________________________________________________________________

________________________________________________________________

________________________________________________________________

________________________________________________________________

________________________________________________________________

c19_anx_cant_c1_qual Please share **one** example of something you’ve been worrying about that **you can't control**

(~1 sentence or even just a few words)

(Example: How fast the virus spreads)

________________________________________________________________

c19_anx_cant_c2_qual Please share **another** example of something you’ve been worrying about that **you can't control**

(~1 sentence or even just a few words)

(Example: How fast the virus spreads)

________________________________________________________________

c19_anx_can_c1_qual Please share **one** example of something you’ve been worrying about that **you can control**

(~1 sentence or even just a few words)

(Example: How I respond to feeling socially disconnected)

________________________________________________________________

c19_anx_can_c2_qual Please share **another** example of something you’ve been worrying about that **you can control**

(~1 sentence or even just a few words)

(Example: How I respond to feeling socially disconnected)

________________________________________________________________

c19_anx_slide_11_tim Timing

First Click (1)

Last Click (2)

Page Submit (3)

Click Count (4)

| Page Break |  |
| --- | --- |

c19_anx_step_2_chose

**Step 2**

Please choose one of the things you've been worrying about that **you can control**

(Bonus points if it's really been bothering you)

o ${c19_anx_can_c1_qual/ChoiceTextEntryValue} (1)

o ${c19_anx_can_c2_qual/ChoiceTextEntryValue} (2)

c19_anx_slide_12_tim Timing

First Click (1)

Last Click (2)

Page Submit (3)

Click Count (4)

| Page Break |  |
| --- | --- |

c19_anx_step_3_qual

**Step 3**

You've chosen to focus on this thing you've been worrying about that **you can control:** ${c19_anx_step_2_chose/ChoiceGroup/SelectedChoices}

Please write **one small step** you can take to help increase your confidence in dealing with this worry. It doesn't have to be perfect, and even "small" steps can make a big difference. (~1-2 sentences)

(Example: I can control how I respond to feeling socially disconnected by reaching out to a few friends to set up Zoom hangouts.)

________________________________________________________________

________________________________________________________________

________________________________________________________________

________________________________________________________________

________________________________________________________________

c19_anx_slide_13_tim Timing

First Click (1)

Last Click (2)

Page Submit (3)

Click Count (4)

| Page Break |  |
| --- | --- |

|  |
| --- |

c19_anx_slide_11 **Thank You For Helping Us!**

Here’s your action plan, which we’ll be putting to use to help others deal with COVID-19 anxiety.

**Step 1:**

You reminded yourself that it's normal to be anxious right now by writing:

*${c19_anx_normal_qual/ChoiceTextEntryValue}*

You told us about these things that are bothering you and **you can't control:**

*${c19_anx_cant_c1_qual/ChoiceTextEntryValue}* **and** *${c19_anx_cant_c2_qual/ChoiceTextEntryValue}*

You told us about these things that are bothering you and **you can control:**

*${c19_anx_can_c1_qual/ChoiceTextEntryValue}* **and** *${c19_anx_can_c2_qual/ChoiceTextEntryValue}*

**Step 2:**

You chose to focus on this thing that was bothering you that you can control:

*${c19_anx_step_2_chose/ChoiceGroup/SelectedChoices}*

**Step 3:**

Your **one small step** toward dealing with **one thing you can control** is:

*${c19_anx_step_3_qual/ChoiceTextEntryValue}*

Here’s a reminder of the 3 steps you can apply to anything you’re stressed about that’s within your control:

1. Remind yourself **it's normal to be anxious**; there are some things you can't control and **some things you can control**

2. **Figure out one thing you can control** that's bothering you

3. Figure out **one small step** you can take to deal better with **one thing you can control** (Repeat this step as many times as necessary)

Please feel free to take screenshots of this page, write down your action plan/the steps in your phone/on paper, or whatever you would like. We would also appreciate you **answering the questions on the following page so we can understand how our program is impacting people**

(We want to change it to make it even better!)

Before we move on though, do you consent to us sharing your action plan with others? It's ok either way, and we appreciate you going through these materials no matter what!

o Yes (1)

o No (0)

c19_anx_slide_14_tim Timing

First Click (1)

Last Click (2)

Page Submit (3)

Click Count (4)

**End of Block: COVID-19 Anxiety SSI**

2 Week Follow-Up Survey

**Between_Sub_Follow_Up_COVID_19_SSI**

**Start of Block: Follow Up**

Q3

We selected these specific questions because previous research has shown they'll help us get a snapshot of how you've been feeling recently.

We know we won't capture everything that makes you who you are, but we appreciate you giving us a glimpse into your life.

You'll spend about 2 minutes answering these questions, and thanks again for helping us understand how you're doing right now!

|  |
| --- |

prolific_id Please confirm your Prolific ID here

________________________________________________________________

|  |
| --- |

f_gad_7 Over the last 2 weeks, how often have you been bothered by the following problems?

|  | Not at all (0) | Several days (1) | More than half the days (2) | Nearly every day (3) |
| --- | --- | --- | --- | --- |
| Feeling nervous, anxious, or on edge (1) | o | o | o | o |
| Not being able to stop or control worrying (2) | o | o | o | o |
| Worrying too much about different things (3) | o | o | o | o |
| Trouble relaxing (4) | o | o | o | o |
| Being so restless that it's hard to sit still (5) | o | o | o | o |
| Becoming easily annoyed or irritable (6) | o | o | o | o |
| Feeling afraid as if something awful might happen (7) | o | o | o | o |

|  |
| --- |

f_acq Listed below are a number of statements describing a set of beliefs. Please read each statement carefully and, on the 0-5 scale given, indicate how much you think each statement is true about you **right now**.

|  | 0 - Strongly Disagree (0) | 1 - Moderately Disagree (1) | 2 - Slightly Disagree (2) | 3 - Slightly Agree (3) | 4 - Moderately Agree (4) | 5 - Strongly Agree (5) |
| --- | --- | --- | --- | --- | --- | --- |
| I am unconcerned if I become anxious in a difficult situation, because I am confident in my ability to cope with my symptoms (1) | o | o | o | o | o | o |
| I can relax when I want (2) | o | o | o | o | o | o |
| I can put worrisome thoughts out of my mind easily (3) | o | o | o | o | o | o |
| I am able to control my level of anxiety (4) | o | o | o | o | o | o |

sesoi_anx Compared to about two weeks ago when you first took our survey, how would you rate the extent of your anxiety right now?

o Much less anxiety (1)

o A little less anxiety (2)

o The same amount of anxiety (3)

o A little more anxiety (4)

o A lot more anxiety (5)

**End of Block: Follow Up**
