# Supplementary material for: Web-Based Single Session Intervention for Perceived Control Over Anxiety During COVID-19: Randomized Controlled Trial
Source: JMIR Ment Health. 2022 Apr 12;9(4):e33473. doi: 10.2196/33473 (PMC9007232; doi:10.2196/33473)
Supplement: Multimedia Appendix 3 [file mental_v9i4e33473_app3.docx]

**COVID_Anxiety_SSI_RR_Preprocessing_and_Analyses**

Mallory L. Dobias and Michael C. Mullarkey

June 05, 2020

*## Packages*
**if**(**!require**(psych)){**install.packages**('psych')}
**library**(psych)
**if**(**!require**(tidyverse)){**install.packages**('tidyverse')}
**library**(tidyverse)
**if**(**!require**(stringr)){**install.packages**('stringr')}
**library**(stringr)
**if**(**!require**(DescTools)){**install.packages**('DescTools')}
**library**(DescTools)
**if**(**!require**(boot)){**install.packages**('boot')}
**library**(boot)
**if**(**!require**(missForest)){**install.packages**('missForest')}
**library**(missForest)
**if**(**!require**(ggstatsplot)){**install.packages**('ggstatsplot')}
**library**(ggstatsplot)
**if**(**!require**(ggthemes)){**install.packages**('ggthemes')}
**library**(ggthemes)
**if**(**!require**(performance)){**install.packages**('performance')}
**library**(performance)
**if**(**!require**(MOTE)){**install.packages**('MOTE')}
**library**(MOTE)
**if**(**!require**(canprot)){**install.packages**('canprot')}
**library**(canprot)
**if**(**!require**(nnet)){**install.packages**('nnet')}
**library**(nnet)
**if**(**!require**(tictoc)){**install.packages**('tictoc')}
**library**(tictoc)
**if**(**!require**(beepr)){**install.packages**('beepr')}
**library**(beepr)
**if**(**!require**(readr)){**install.packages**('readr')}
**library**(readr)
**if**(**!require**(Hmisc)){**install.packages**('Hmisc')}
**library**(Hmisc)
**if**(**!require**(RColorBrewer)){**install.packages**('RColorBrewer')}
**library**(RColorBrewer)
**if**(**!require**(reshape2)){**install.packages**('reshape2')}
**library**(reshape2)
**if**(**!require**(randomForest)){**install.packages**('randomForest')}
**library**(randomForest)
*#install.packages("devtools")*
*#devtools::install_github("jashu/beset", build_vignettes = F)*
**library**(beset)
**if**(**!require**(summarytools)){**install.packages**('summarytools')}
**library**(summarytools)
**if**(**!require**(lubridate)){**install.packages**('lubridate')}
**library**(lubridate)
**if**(**!require**(broom)){**install.packages**('broom')}
**library**(broom)
**if**(**!require**(cowplot)){**install.packages**('cowplot')}
**library**(cowplot)
**if**(**!require**(metafor)){**install.packages**('metafor')}
**library**(metafor)
**if**(**!require**(gridExtra)){**install.packages**('gridExtra')}
**library**(gridExtra)
**if**(**!require**(cocor)){**install.packages**('cocor')}
**library**(cocor)
**if**(**!require**(factoextra)){**install.packages**('factoextra')}
**library**(factoextra)
**if**(**!require**(Amelia)){**install.packages**('Amelia')}
**library**(Amelia)
**if**(**!require**(TOSTER)){**install.packages**('TOSTER')}
**library**(TOSTER)
**if**(**!require**(car)){**install.packages**('car')}
**library**(car)
**if**(**!require**(stats)){**install.packages**('stats')}
**library**(stats)
**if**(**!require**(magrittr)){**install.packages**('magrittr')}
**library**(magrittr)

## **Data Masking**

Test responses generated in qualtrics on June 4, 2020 (N = 500). This first chunk will be run in a separate R file by JLS before sending the .csv output at the end of this code to MCM for masked analyses.

*# Loading raw data (Downloaded as numeric from Qualtrics)*
raw_baseline <- **read.csv**(file = "test_baseline_data_prolific_covid_SSI_June_4_2020.csv", header = TRUE, sep = ",", stringsAsFactors = F)
*#View(head(raw, n = 3)) # looks like there's extra info from qualtrics*

*## This code will happen in a separate file, put here for simplicity: creating labels for condition*

*# Deleting first two, unnecessary rows with extra info from qualtrics*
raw_baseline2 <- raw_baseline[**-c**(1,2),]

*## Seeing how many participants we have in each condition before we start the masking process*

raw_baseline2 **%>%**
 **group_by**(cond) **%>%**
 **tally**()

## # A tibble: 2 x 2
## cond n
## <chr> <int>
## 1 0 250
## 2 1 250

*## Only for simulation adding fake Prolific IDs*

raw_baseline2 <- raw_baseline2 **%>%**
 **mutate**(prolific_id = 1**:**500)

*## Now read in follow-up data*

raw_follow_up <- **read.csv**(file = "test_fu_data_prolific_covid_SSI_June_4_2020.csv", header = TRUE, sep = ",", stringsAsFactors = F)

*# Deleting first two, unnecessary rows with extra info from qualtrics*
raw_follow_up <- raw_follow_up[**-c**(1,2),]

*## Only for simulation adding fake Prolific IDs*

raw_follow_up <- raw_follow_up **%>%**
 **mutate**(prolific_id = 1**:**500)

*## And join based on Prolific ID*

raw_combined <- **left_join**(raw_baseline2, raw_follow_up, by = "prolific_id")

*## This process will allow us to mask intervention order. The senior author JLS will e-mail her decision to CGB and not disclose her decision to any other authors, especially MCM who will conduct the primary analyses.*

raw_combined <- raw_combined **%>%**
 **mutate**(cond = **case_when**(cond **==** "0" **~** "Y", *# or Z*
 cond **==** "1" **~** "Z", *# or Y*
 TRUE **~** NA_character_))

**write.csv**(raw_combined, "test_data_masked_intervention_covid_SSI.csv")

## **Reading in Masked Data**

Starting here (plus the packages + root.dir chunk) will be the coding file MCM will use to analyze the data in a masked manner.

raw2_masked <- **read.csv**(file = "test_data_masked_intervention_covid_SSI.csv", header = TRUE, sep = ",", stringsAsFactors = F)

*## This process will allow us to mask intervention order. The lead author MCM will e-mail his decision to CGB and not disclose his decision to any other authors, especially JLS who did the original masking of the intervention_order variable. CGB will put the information from MCM and JLS in a Box file that will not be shared with other members of the team until the primary analyses are completed. This code will be modified once the conditions are unmasked to facilitate full interpretation of the results.*

raw2_masked <- raw2_masked **%>%**
 **mutate**(cond = **case_when**(cond **==** "Y" **~** "0", *# or 1*
 cond **==** "Z" **~** "1", *# or 0*
 TRUE **~** NA_character_))

## **Cleaning/Preprocessing Data**

*# Selecting variables - all collected variables, minus time variables and minus character variables (for imputation)*
raw3 <- raw2_masked **%>%** dplyr**::select**(age, race_eth, gender, sexual_orientation, edu_level, income**:**ideology, covid_sick**:**c19_impacts, b_idas_dysphoria_1**:**b_self_hate_7, b_acq_1**:**b_hand_wash_int_3, c19_anx_comp_1_t1**:**pi_hand_wash_int_3, c19_anx_step_2_chose, c19_anx_slide_11, c19_anx_comp_1_t1**:**cond, survey_complete,prolific_id,sesoi_anx,**contains**("f_gad_7_"),**contains**("f_acq_"))

*# Converting empty cells to NAs*
raw4 <- raw3 **%>%** **mutate_all**(na_if,"")

raw4**$**f_acq_1

## [1] 3 5 4 2 1 4 2 3 2 3 2 2 5 4 0 0 1 5 5 2 1 1 2 0 0 2 4 5 5 5 3 3 3 3 0 1 1
## [38] 5 4 1 5 0 0 3 5 0 4 4 5 3 3 1 1 2 0 3 5 0 4 1 0 5 1 5 0 2 1 2 1 4 2 4 5 5
## [75] 4 4 1 1 5 0 3 0 3 2 5 2 4 4 0 2 3 0 5 2 3 1 0 1 1 5 4 3 1 5 0 1 2 5 5 5 4
## [112] 0 5 1 1 2 0 4 2 0 5 5 0 0 5 1 3 4 0 5 2 3 0 0 0 1 0 5 5 4 5 0 4 4 2 1 0 3
## [149] 1 3 3 3 5 3 1 0 1 1 5 1 4 1 0 5 5 1 1 2 2 5 0 1 3 4 3 0 2 2 1 2 5 3 4 0 3
## [186] 4 0 1 4 4 4 4 1 2 4 2 1 4 2 0 3 2 5 3 4 2 5 2 2 1 2 2 3 5 0 5 2 0 2 0 2 4
## [223] 2 4 3 4 5 4 0 3 4 0 3 2 3 2 3 0 0 2 5 3 1 2 5 2 2 1 5 1 3 2 4 3 2 0 0 5 1
## [260] 1 2 5 5 4 2 0 5 0 5 1 5 4 3 3 3 5 4 0 3 2 4 5 3 0 0 4 1 1 0 4 3 1 1 3 4 4
## [297] 5 2 4 5 3 4 0 4 1 0 2 1 5 4 3 3 1 1 5 1 1 4 1 1 4 3 4 0 5 1 5 5 0 5 3 5 4
## [334] 3 3 1 3 1 5 1 3 2 0 3 5 1 5 3 4 5 4 4 3 1 5 4 0 3 4 0 3 3 2 2 0 5 4 1 4 1
## [371] 4 4 3 0 0 5 5 4 0 5 5 2 2 5 0 0 5 0 5 0 5 1 1 3 4 2 3 0 3 1 4 3 0 1 5 1 3
## [408] 5 2 1 0 3 1 3 5 1 4 5 3 1 4 4 1 0 1 3 5 2 0 2 4 3 4 5 3 5 5 0 3 2 2 5 3 1
## [445] 1 3 5 3 1 3 3 2 3 1 0 2 2 3 4 3 2 1 4 3 3 2 1 5 4 4 5 2 0 1 4 3 0 0 5 2 3
## [482] 4 0 4 2 1 4 1 3 1 2 0 0 2 4 3 2 5 2 0

*# Converting variable classes and recoding (must be character or numeric to impute)*
raw5 <- raw4 **%>%** **mutate**(age = **as.numeric**(age), race_eth = **as.factor**(race_eth),
 race_eth = **recode_factor**(race_eth, `1` = "American Indian and/or Alaska Native",
 `2` = "Asian",
 `3` = "African American",
 `4` = "Hispanic or Latino/a",
 `5` = "Native Hawaiian or Pacific Islander",
 `6` = "White, Non-Hispanic",
 `7` = "More than one race",
 `8` = "Other"),
 gender = **as.factor**(gender),
 gender = **recode_factor**(gender, `1` = "Agender",
 `2` = "Androgyne",
 `3` = "Demigender",
 `4` = "Genderqueer or gender fluid",
 `5` = "Man",
 `6` = "Questioning or unsure",
 `7` = "Trans man",
 `8` = "Trans woman",
 `9` = "Woman",
 `10` = "Other"),
 sexual_orientation = **as.factor**(sexual_orientation),
 sexual_orientation = **recode_factor**(sexual_orientation, `1` = "Asexual",
 `2` = "Bisexual",
 `3` = "Gay",
 `4` = "Heterosexual",
 `5` = "Lesbian",
 `6` = "Pansexual",
 `7` = "Queer",
 `8` = "Questioning or unsure",
 `9` = "Same-gender loving",
 `10` = "Other"),
 edu_level = **as.factor**(edu_level),
 edu_level = **recode_factor**(edu_level, `1` = "Less than high school degree",
 `2` = "High school degree",
 `3` = "Some college, no degree",
 `4` = "Associate degree",
 `5` = "Bachelor's degree",
 `6` = "Master's degree",
 `7` = "Professional degree",
 `8` = "Doctorate"),
 income = **as.factor**(income),
 income = **recode_factor**(income, `1` = "Less than $10,000",
 `2` = "$10,000 to $19,999",
 `3` = "$20,000 to $29,999",
 `4` = "$30,000 to $39,999",
 `5` = "$40,000 to $49,999",
 `6` = "$50,000 to $59,999",
 `7` = "$60,000 to $69,999",
 `8` = "$70,000 to $79,999",
 `9` = "$80,000 to $89,999",
 `10` = "$90,000 to $99,999",
 `11` = "$100,000 to $149,999",
 `12` = "$150,000 or more"),
 relationship = **as.factor**(relationship),
 relationship = **recode_factor**(relationship, `1` = "No current relationship",
 `2` = "Relationship, not living together",
 `3` = "Relationship, living together",
 `4` = "Engaged",
 `5` = "Married"),
 children = **as.factor**(children),
 children = **recode_factor**(children, `0` = "No",
 `1` = "Yes"),
 insurance_mh = **as.factor**(insurance_mh),
 insurance_mh = **recode_factor**(insurance_mh, `0` = "No",
 `1` = "Yes"),
 rec_therapy = **as.factor**(rec_therapy),
 rec_therapy = **recode_factor**(rec_therapy, `0` = "No",
 `1` = "Yes"),
 rec_medication = **as.factor**(rec_medication),
 rec_medication = **recode_factor**(rec_medication, `0` = "No",
 `1` = "Yes"),
 perceived_need = **as.factor**(perceived_need),
 perceived_need = **recode_factor**(perceived_need, `0` = "No",
 `1` = "Yes"),
 ideology = **as.numeric**(ideology),

 covid_sick = **as.factor**(covid_sick),
 covid_sick = **recode_factor**(covid_sick, `0` = "No",
 `1` = "Yes"),
 covid_symptoms = **as.factor**(covid_symptoms),
 covid_symptoms = **recode_factor**(covid_symptoms, `1` = "fever",
 `2` = "cough",
 `3` = "sore throat",
 `4` = "runny or stuffy nose",
 `5` = "difficulty breathing"),
 covid_test_or_not = **as.factor**(covid_test_or_not),
 covid_test_or_not = **recode_factor**(covid_test_or_not, `0` = "No",
 `1` = "Yes"),
 covid_test_positive = **as.factor**(covid_test_positive),
 covid_test_positive = **recode_factor**(covid_test_positive, `0` = "No",
 `1` = "Yes",
 `2` = "Unknown"),
 schools_closed = **as.factor**(schools_closed),
 schools_closed = **recode_factor**(schools_closed, `0` = "No",
 `1` = "Yes")) **%>%**

 **mutate_at**(**vars**(**contains**("b_idas_")), **funs**(dplyr**::recode**(., '1' = 1, '2' = 2, '3' = 3, '4' = 4, '5' = 5))) **%>%**
 **mutate_at**(**vars**(**contains**("b_gad_7_")), **funs**(dplyr**::recode**(., '0' = 0, '1' = 1, '2' = 2, '3' = 3))) **%>%**
 **mutate_at**(**vars**(**contains**("b_self_hate_")), **funs**(dplyr**::recode**(., '0' = 0, '1' = 1, '2' = 2, '3' = 3, '4' = 4, '5' = 5, '6' = 6, '7' = 7))) **%>%**
 **mutate_at**(**vars**(**contains**("b_acq_")), **funs**(dplyr**::recode**(., '0' = 0, '1' = 1, '2' = 2, '3' = 3, '4' = 4, '5' = 5))) **%>%**
 **mutate_at**(**vars**(**contains**("b_soc_dist_")), **funs**(dplyr**::recode**(., '1' = 1, '2' = 2, '3' = 3, '4' = 4))) **%>%**
 **mutate_at**(**vars**(**contains**("b_hand_wash_int_")), **funs**(dplyr**::recode**(., '1' = 1, '2' = 2, '3' = 3, '4' = 4, '5' = 5, '6' = 6, '7' = 7))) **%>%**
 **mutate**(
 c19_anx_comp_1_t1 = **as.factor**(c19_anx_comp_1_t1),
 c19_anx_comp_1_t1 = **recode_factor**(c19_anx_comp_1_t1, `1` = "only can't control",
 `2` = "only can control",
 `3` = "both",
 `4` = "none"),
 c19_anx_comp_2_t1 = **as.factor**(c19_anx_comp_2_t1),
 c19_anx_comp_2_t1 = **recode_factor**(c19_anx_comp_2_t1, `1` = "one thing you can control",
 `2` = "two things you can control",
 `3` = "one thing you can't control",
 `4` = "two things you can't control"),
 c19_hw_comp_1_t1 = **as.factor**(c19_hw_comp_1_t1),
 c19_hw_comp_1_t1 = **recode_factor**(c19_hw_comp_1_t1, `1` = "pick song",
 `2` = "pick celebrity",
 `3` = "list reasons",
 `4` = "none"),
 c19_hw_comp_2_t1 = **as.factor**(c19_hw_comp_2_t1),
 c19_hw_comp_2_t1 = **recode_factor**(c19_hw_comp_2_t1, `1` = "choose alarms",
 `2` = "how many reminders",
 `3` = "how often wash hands",
 `4` = "how long wash hands")) **%>%**
 **mutate_at**(**vars**(**contains**("pi_acq_")), **funs**(dplyr**::recode**(., '0' = 0, '1' = 1, '2' = 2, '3' = 3, '4' = 4, '5' = 5))) **%>%**
 **mutate_at**(**vars**(**contains**("pi_hand_wash_int_")), **funs**(dplyr**::recode**(., '1' = 1, '2' = 2, '3' = 3, '4' = 4, '5' = 5, '6' = 6, '7' = 7))) **%>%**
 **mutate**(c19_anx_step_2_chose = **as.factor**(c19_anx_step_2_chose),
 c19_anx_step_2_chose = **recode_factor**(c19_anx_step_2_chose, `1` = "Piped text 1",
 `2` = "Piped text 2")) **%>%**
 **mutate**(c19_anx_slide_11 = **as.factor**(c19_anx_slide_11),
 c19_anx_slide_11 = **recode_factor**(c19_anx_slide_11, `1` = "Yes",
 `0` = "No")) **%>%**
 **mutate_at**(**vars**(**contains**("f_acq_")), **funs**(dplyr**::recode**(., '0' = 0, '1' = 1, '2' = 2, '3' = 3, '4' = 4, '5' = 5))) **%>%**
 **mutate_at**(**vars**(**contains**("pi_soc_dist_")), **funs**(dplyr**::recode**(., '0' = 0, '1' = 1, '2' = 2, '3' = 3, '4' = 4))) **%>%**
 **mutate_at**(**vars**(**contains**("pi2_hand_wash_int_")), **funs**(dplyr**::recode**(., '1' = 1, '2' = 2, '3' = 3, '4' = 4, '5' = 5, '6' = 6, '7' = 7))) **%>%**
 **mutate_at**(**vars**(**contains**("f_gad_7_")), **funs**(dplyr**::recode**(., '0' = 0, '1' = 1, '2' = 2, '3' = 3))) **%>%**
 **mutate**(cond = **as.factor**(cond),
 sesoi_anx = **as.numeric**(sesoi_anx)) **%>%**
 **mutate**(survey_complete = **as.factor**(survey_complete)) **%>%**
 **mutate_if**(is.numeric, as.integer)

## **Performing the Quality Check**

Need at least 75% of people to answer both questions following the Contain COVID Anxiety SSI to proceed with analyses.

*## For simulated data we should see 250 in each group and we do*

data **%>%**
 **group_by**(cond) **%>%**
 **tally**()

## # A tibble: 2 x 2
## cond n
## <fct> <int>
## 1 0 250
## 2 1 250

*## Let's create the "how many people got both comprehension questions correct" variable*

data <- data **%>%**
 **mutate**(
 anx_ssi_question_1_correct = **case_when**(c19_anx_comp_1_t1 **==** "both" **~** 1,
 TRUE **~** 0),
 anx_ssi_question_2_correct = **case_when**(c19_anx_comp_2_t1 **==** "one thing you can control" **~** 1,
 TRUE **~** 0),
 both_anx_ssi_questions_correct = **ifelse**(anx_ssi_question_1_correct **==** 1 **&**
 anx_ssi_question_2_correct **==** 1,1,0)
 )

*## Then see how many people got both questions correct (We're also counting NAs as incorrect based on how we code this to be even more conservative.) In the random simulated data only 13 people got both questions correct*

answered_both_comp_correctly <- data **%>%**
 **group_by**(both_anx_ssi_questions_correct) **%>%**
 **tally**() **%>%**
 **print**()

## # A tibble: 2 x 2
## both_anx_ssi_questions_correct n
## <dbl> <int>
## 1 0 478
## 2 1 22

*## This calculates the percentage of people who got both questions correct regardless of final sample size.*
*## In this simulated, random data we only see 4.4% of the sample answering both correctly. In that case we would stop. Only if this percentage is 75% or higher do we proceed with the rest of our confirmatory analyses.*

answered_both_comp_correctly_percent <- answered_both_comp_correctly **%>%**
 **ungroup**() **%>%**
 **mutate**(countT= **sum**(n)) **%>%**
 **mutate**(percent_both_anx_ssi_questions_correct=**paste0**(**round**(100*****n**/**countT,2),'%')) **%>%**
 **filter**(both_anx_ssi_questions_correct **==** 1) **%>%**
 dplyr**::select**(percent_both_anx_ssi_questions_correct) **%>%**
 **print**()

## # A tibble: 1 x 1
## percent_both_anx_ssi_questions_correct
## <chr>
## 1 4.4%

## **Testing for Differential Dropout Based on Observed Treatment Condition**

Here’s where we run a two-proportions Z-test, where we compare the proportion of people who drop out before completing the study (0 = no; 1 = yes) as a function of treatment assignment (0 = washing hands SSI; 1 = covid anxiety SSI)

I’ll use this resource: <http://www.sthda.com/english/wiki/two-proportions-z-test-in-r>

I created an embedded data variable in the qualtrics survey flow (“survey_complete”) to determine who finished vs. did not finish their survey. Note: This code won’t run in the simulated data without modifying it, as qualtrics has generated 500 responses that are all “complete” surveys. But, I’ll go ahead and write the code now.

*# Selecting variables*
chi_df <- data **%>%** dplyr**::select**(cond, survey_complete)

*# Since all surveys are coding as complete, let's throw some random non-completes in there (Only for simulated data, this will be commented out for actual analyses)*

chi_df <- chi_df **%>%**
 **mutate**(survey_complete = **sample**(0**:**1, size = 500, replace = TRUE),
 cond_temp = **as.character**(cond),
 cond = **as.numeric**(cond),
 cond = **case_when**(
 cond **==** 1 **~** 0,
 cond **==** 2 **~** 1,
 )) **%>%**
 dplyr**::select**(**-**cond_temp)

chi_table <-**table**(chi_df**$**cond, chi_df**$**survey_complete, deparse.level = 2)

*## Can see here which variables are which and which values correspond to their placement in the table*
chi_table

## chi_df$survey_complete
## chi_df$cond 0 1
## 0 134 116
## 1 136 114

chi_table["0","0"]

## [1] 134

chi_table["1","0"]

## [1] 136

*# Running the the proportions Z test (Proportion of Dropouts based on total number of people who were randomized to each intervention order)*
**prop.test**(x = **c**(chi_table["0","0"], chi_table["1","0"]), n = **c**(chi_table["0","0"]**+**chi_table["0","1"],
 chi_table["1","0"]**+**chi_table["1","1"]))

##
## 2-sample test for equality of proportions with continuity correction
##
## data: c(chi_table["0", "0"], chi_table["1", "0"]) out of c(chi_table["0", "0"] + chi_table["0", "1"], chi_table["1", "0"] + c(chi_table["0", "0"], chi_table["1", "0"]) out of chi_table["1", "1"])
## X-squared = 0.0080515, df = 1, p-value = 0.9285
## alternative hypothesis: two.sided
## 95 percent confidence interval:
## -0.0993685 0.0833685
## sample estimates:
## prop 1 prop 2
## 0.536 0.544

## **Imputing Data**

Here’s where we use the Amelia Package/multiple imputation in R to impute data for our main outcomes (genrealized anxiety at 2 week follow-up, perceived control of anxiety post SSI, and social distancing intentions post SSI).

Note: we have to manually drop data from the simulated data, and won’t run that code on the actual data.

We used the following variables to impute data for our three hypotheses:

1. Hypothesis 1 (GAD-7/generalized anxiety): baseline gad 7 mean, baseline acq, condition, idas dysphoria mean, binary whether they received meds or therapy in the past 12 months (either or neither), and self-hate mean
2. Hypothesis 2 (social distancing): baseline social distancing, condition, age, gender (male, female, non-binary), education level, income level
3. Hypothesis 3 (perceived control of anxiety): baseline acq, intervention order, idas dysphoria mean, baseline gad 7 mean, binary whether they received meds or therapy in the past 12 months (either or neither), and self-hate mean

*# Imputing data for hypothesis 1/3*

*# Selecting variables for perceived control post-first SSI imputation*
acq_imp <- data **%>%** **select**(id, cond, b_idas_dysphoria_mean, b_gad_7_mean, f_gad_7_mean,
 b_self_hate_mean, rec_mh_care_for_imp, b_acq_mean, pi_acq_mean, f_acq_mean)

*# For simulated data only, have to randomly remove some of the data (In this case 20%)*

acq_imp <- **prodNA**(acq_imp, 0.20)

*# Creating matrix to set bounds for ALL acq outcome variables; column number, min value, max value*
acq_bounds_all <- **matrix**(**c**(8,0,5, 9,0,5), nrow = 2, ncol = 3, byrow = TRUE) *# Check with Mallory re: GAD-7*

*# Running the multiple imputation model*
*# Note, we use 20 for this example, but will change m to match the % of missing data in our outcome variables*
**set.seed**(13)
acq_out <- **amelia**(acq_imp, m = 20, noms = **c**("cond", "rec_mh_care_for_imp"), idvars = "id",
 bounds=acq_bounds_all)

## -- Imputation 1 --
##
## 1 2 3 4 5 6 7 8 9 10 11 12 13
##
## -- Imputation 2 --
##
## 1 2 3 4 5 6 7 8 9 10 11
##
## -- Imputation 3 --
##
## 1 2 3 4 5 6 7 8 9 10 11
##
## -- Imputation 4 --
##
## 1 2 3 4 5 6 7 8 9 10 11
##
## -- Imputation 5 --
##
## 1 2 3 4 5 6 7 8 9 10 11 12
##
## -- Imputation 6 --
##
## 1 2 3 4 5 6 7 8 9 10
##
## -- Imputation 7 --
##
## 1 2 3 4 5 6 7 8 9 10
##
## -- Imputation 8 --
##
## 1 2 3 4 5 6 7 8 9 10 11 12
##
## -- Imputation 9 --
##
## 1 2 3 4 5 6 7 8 9 10 11
##
## -- Imputation 10 --
##
## 1 2 3 4 5 6 7 8 9 10 11 12
##
## -- Imputation 11 --
##
## 1 2 3 4 5 6 7 8 9 10 11 12
##
## -- Imputation 12 --
##
## 1 2 3 4 5 6 7 8 9 10 11 12
##
## -- Imputation 13 --
##
## 1 2 3 4 5 6 7 8 9 10 11 12 13
##
## -- Imputation 14 --
##
## 1 2 3 4 5 6 7 8 9 10
##
## -- Imputation 15 --
##
## 1 2 3 4 5 6 7 8 9 10 11 12
##
## -- Imputation 16 --
##
## 1 2 3 4 5 6 7 8 9 10 11 12
##
## -- Imputation 17 --
##
## 1 2 3 4 5 6 7 8 9 10 11
##
## -- Imputation 18 --
##
## 1 2 3 4 5 6 7 8 9 10 11
##
## -- Imputation 19 --
##
## 1 2 3 4 5 6 7 8 9 10 11
##
## -- Imputation 20 --
##
## 1 2 3 4 5 6 7 8 9 10 11

*# Creating diagnostic plots*
**plot**(acq_out, which.vars = 7**:**9)
**par**(mfrow=(**c**(1,1)))


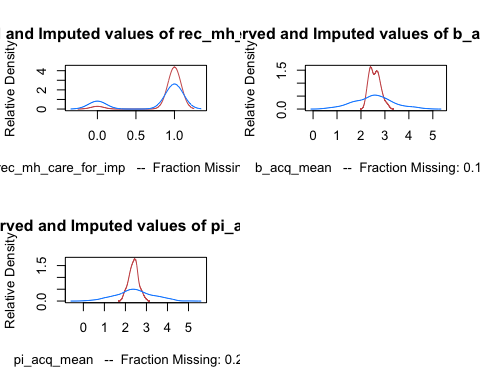


**compare.density**(acq_out, var = "f_gad_7_mean")


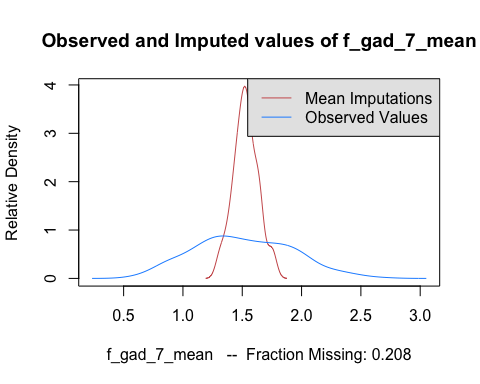


**overimpute**(acq_out, var = "f_gad_7_mean")


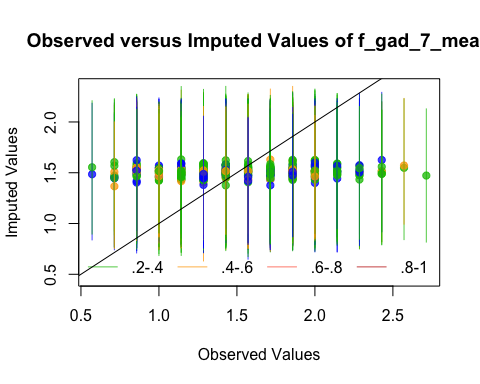


**missmap**(acq_out)


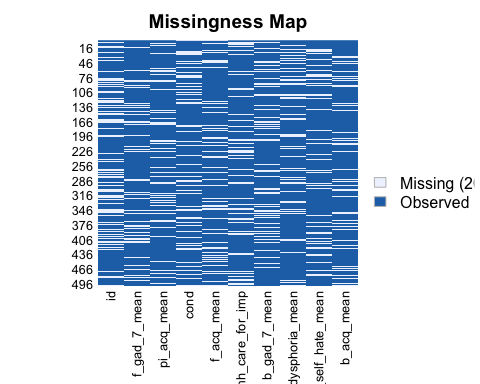


**plot**(acq_out, which.vars = 7**:**9)
**par**(mfrow=(**c**(1,1)))


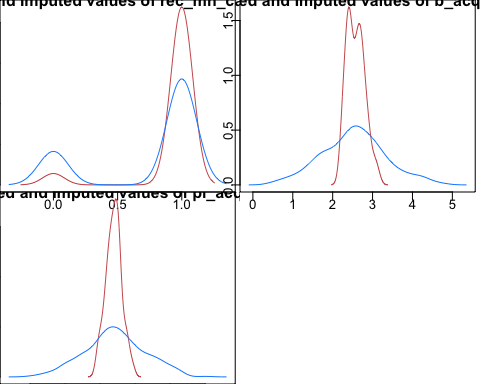


**compare.density**(acq_out, var = "pi_acq_mean")


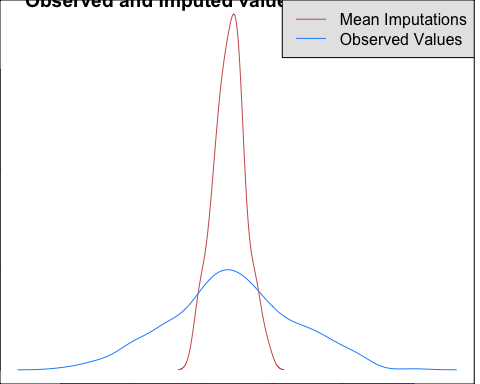


**overimpute**(acq_out, var = "pi_acq_mean")


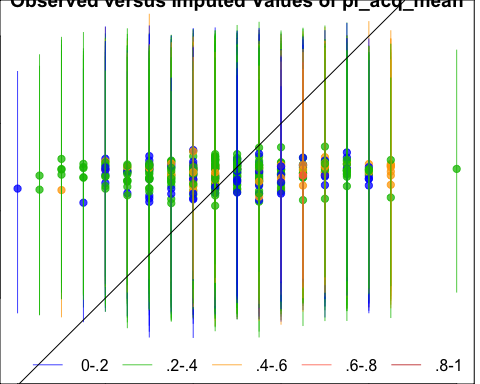


**missmap**(acq_out)


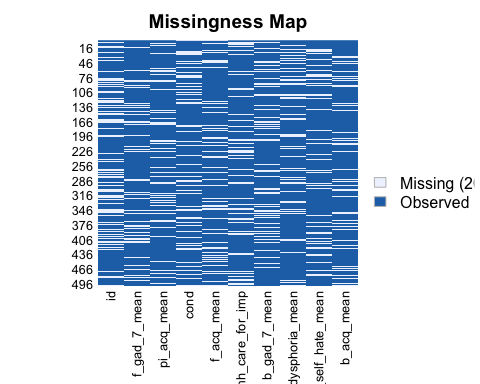


*# Imputing data for hypothesis 2*

*# Selecting variables for perceived control post-first SSI imputation*
soc_dist_imp <- data **%>%** **select**(id, cond, age, gender, edu_level, income,
 b_soc_dist_mean, pi_soc_dist_mean)

*# For simulated data only, have to randomly remove some of the data (In this case 20%)*

soc_dist_imp <- **prodNA**(soc_dist_imp, 0.20)

*# Creating matrix to set bounds for ALL soc dist outcome variables; column number, min value, max value*
soc_dist_bounds_all <- **matrix**(**c**(7,1,4, 8,1,4), nrow = 2, ncol = 3, byrow = TRUE)

*# Running the multiple imputation model*
**set.seed**(13)
soc_dist_out <- **amelia**(soc_dist_imp, m = 20, noms = **c**("cond", "gender"),
 ords = **c**("edu_level", "income"), idvars = "id",
 bounds=soc_dist_bounds_all)

## -- Imputation 1 --
##
## 1 2 3 4 5 6 7 8 9 10 11 12
##
## -- Imputation 2 --
##
## 1 2 3 4 5 6 7 8 9 10 11 12 13
##
## -- Imputation 3 --
##
## 1 2 3 4 5 6 7 8 9 10 11
##
## -- Imputation 4 --
##
## 1 2 3 4 5 6 7 8 9 10 11 12
##
## -- Imputation 5 --
##
## 1 2 3 4 5 6 7 8 9 10 11 12 13
##
## -- Imputation 6 --
##
## 1 2 3 4 5 6 7 8 9 10 11 12
##
## -- Imputation 7 --
##
## 1 2 3 4 5 6 7 8 9 10 11 12 13
##
## -- Imputation 8 --
##
## 1 2 3 4 5 6 7 8 9 10 11 12
##
## -- Imputation 9 --
##
## 1 2 3 4 5 6 7 8 9 10 11 12 13
##
## -- Imputation 10 --
##
## 1 2 3 4 5 6 7 8 9 10 11 12 13 14
##
## -- Imputation 11 --
##
## 1 2 3 4 5 6 7 8 9 10 11 12
##
## -- Imputation 12 --
##
## 1 2 3 4 5 6 7 8 9 10 11 12 13
##
## -- Imputation 13 --
##
## 1 2 3 4 5 6 7 8 9 10 11 12
##
## -- Imputation 14 --
##
## 1 2 3 4 5 6 7 8 9 10 11 12 13
##
## -- Imputation 15 --
##
## 1 2 3 4 5 6 7 8 9 10 11 12 13
##
## -- Imputation 16 --
##
## 1 2 3 4 5 6 7 8 9 10 11 12 13 14
##
## -- Imputation 17 --
##
## 1 2 3 4 5 6 7 8 9 10 11 12
##
## -- Imputation 18 --
##
## 1 2 3 4 5 6 7 8 9 10 11 12 13
##
## -- Imputation 19 --
##
## 1 2 3 4 5 6 7 8 9 10 11 12 13
##
## -- Imputation 20 --
##
## 1 2 3 4 5 6 7 8 9 10 11 12 13

*# Creating diagnostic plots*
**plot**(soc_dist_out, which.vars = 7**:**8)


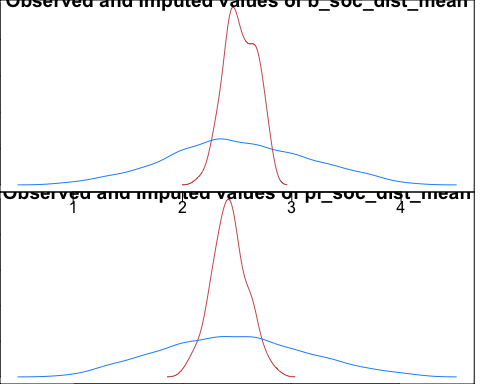


**par**(mfrow=(**c**(1,1)))
**compare.density**(soc_dist_out, var = "pi_soc_dist_mean")


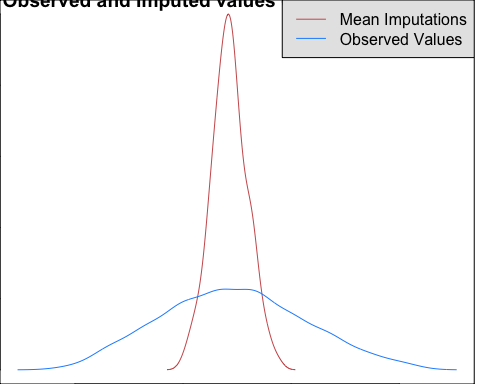


**overimpute**(soc_dist_out, var = "pi_soc_dist_mean")


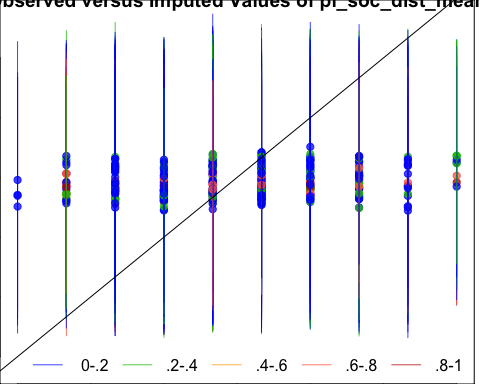


**missmap**(soc_dist_out)


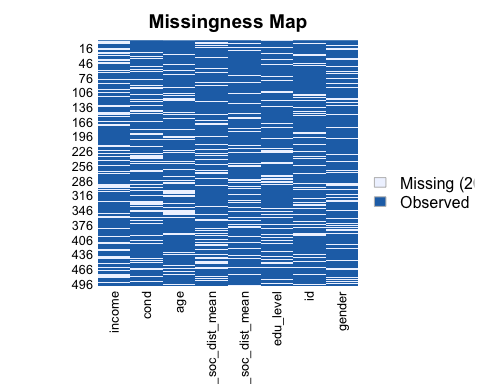


*# Imputing data for hypothesis 3*

*# For simulated data only, have to randomly remove some of the data for pi_acq_mean and f_acq_mean*

*# Selecting variables for perceived control post-first SSI imputation*
*# acq_imp <- data %>% select(id, cond, b_idas_dysphoria_mean, b_gad_7_mean,*
*# b_self_hate_mean, rec_mh_care_for_imp, b_acq_mean, pi_acq_mean, f_acq_mean)*
*#*
*# # Creating matrix to set bounds for ALL acq outcome variables; column number, min value, max value*
*# acq_bounds_all <- matrix(c(8,0,5, 9,0,5), nrow = 2, ncol = 3, byrow = TRUE)*
*#*
*# # Running the multiple imputation model*
*# # Note, we use 20 for this example, but will change m to match the % of missing data in our outcome variables*
*# set.seed(13)*
*# acq_out <- amelia(acq_imp, m = 20, noms = c("cond", "rec_mh_care_for_imp"), idvars = "id",*
*# bounds=acq_bounds_all)*
*#*
*# # Creating diagnostic plots*
*# plot(acq_out, which.vars = 7:9)*
*# par(mfrow=(c(1,1)))*
*# compare.density(acq_out, var = "pi_acq_mean")*
*#*
*# overimpute(acq_out, var = "pi_acq_mean")*
*# missmap(acq_out)*

## **Does the COVID Anxiety SSI Decrease GAD-7 Scores More Than the Control? Directly Testing the Alternative Hypothesis of Hypothesis 1**

Testing hypothesis 1 using a linear regression. We first examine a list all of the multiply imputed datasets.

*# Amelia creates a list of dataframes equal to our number of imputations, which we can pass to the first argument of the 'map' function in the purrr package*

**class**(acq_out**$**imputations)

## [1] "mi" "list"

*## A template for basic map() usage: map(YOUR_LIST, YOUR_FUNCTION)*
*## This allows us to return a list of dataframes (using map) that's flexible across number of imputations.*

*## See https://community.rstudio.com/t/simplest-way-to-modify-the-same-column-in-multiple-dataframes-in-a-list/13076 for code I modified to make this happen*

*## This tutorial for map was also helpful! https://jennybc.github.io/purrr-tutorial/ls01_map-name-position-shortcuts.html#load_packages*
*## Branched off of these resources https://jennybc.github.io/purrr-tutorial/*

## **Assumption Checks for Hypothesis 1**

These assumption checks are all visual in nature without hard cut-offs. Therefore, if we choose to conduct any data transformations we will present both the non-transformed results and the transformed results (along with the results of all assumption checks).

*# Checking assumptions in imputed data*

*# First have to run the linear model of interest on all imputed datasets*

gad_lms_no_summ_for_all_imps <- **map**(acq_out**$**imputations, **~**{
 **lm**(f_gad_7_mean **~** b_gad_7_mean **+** cond, data = .x)
})

*## Checking linearity & homoscedasticity using residuals vs. predicted values plot*

*# Do it once*

**plot**(gad_lms_no_summ_for_all_imps**$**imp1**$**fitted.values, gad_lms_no_summ_for_all_imps**$**imp1**$**residuals)


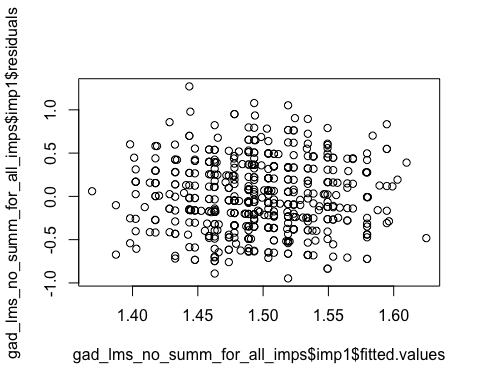


*# Do it for all imputations HAVE TO CALL THE WHOLE THING, NOT JUST THE VARIABLE*

gad_linearity_scedast_for_all_imps <- **map**(acq_out**$**imputations, **~**{
 fitted <- **lm**(f_gad_7_mean **~** b_gad_7_mean **+** cond, data = .x)**$**fitted.values
 residuals <- **lm**(f_gad_7_mean **~** b_gad_7_mean **+** cond, data = .x)**$**residuals
 **plot**(fitted,residuals)
})


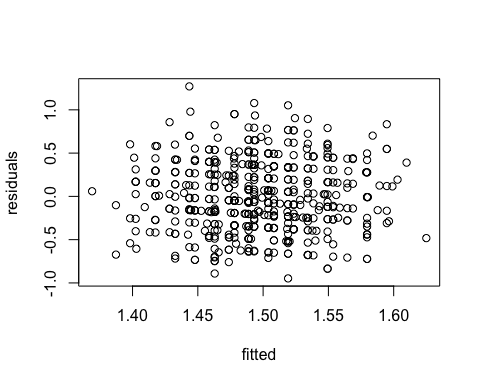

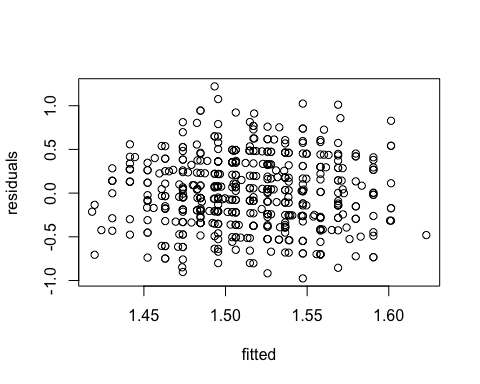

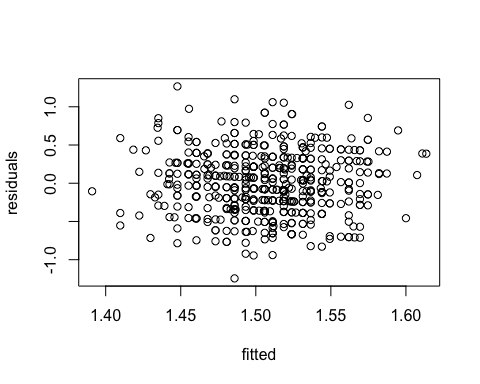

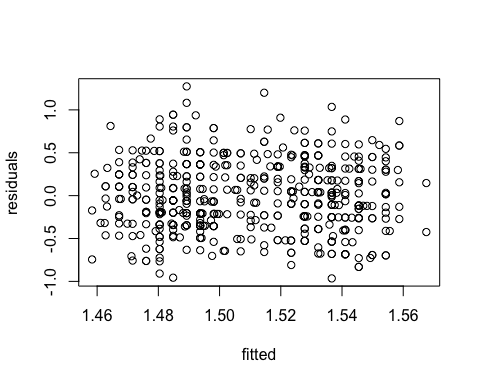

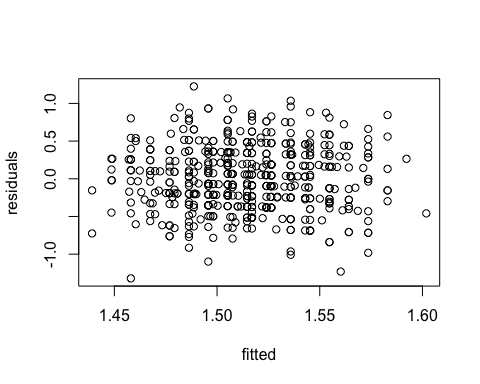

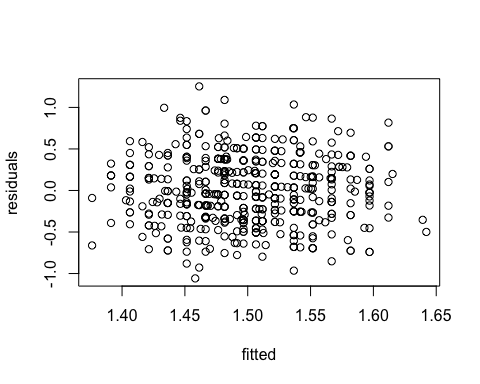

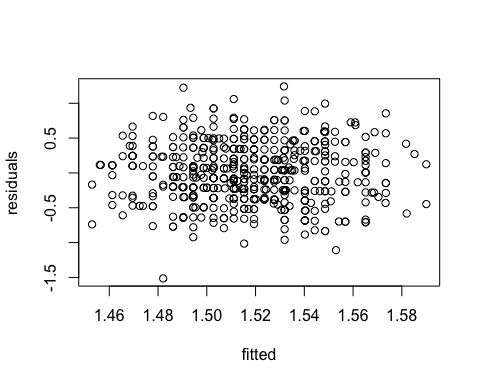

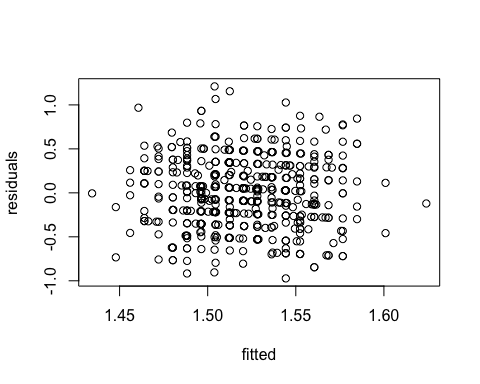

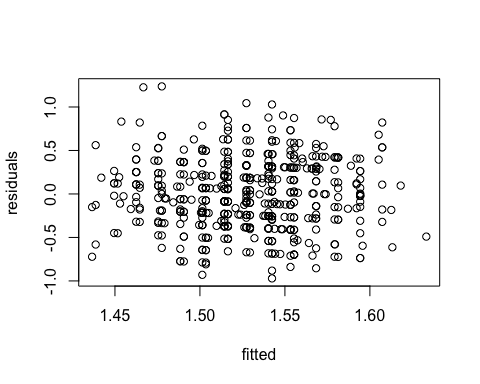

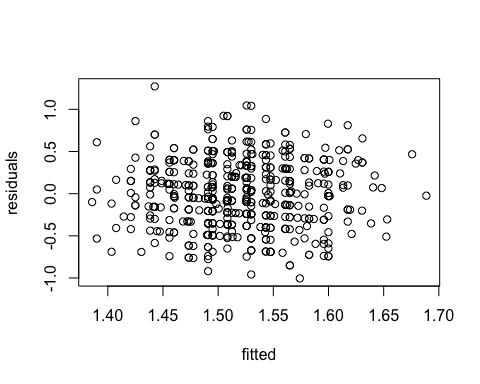

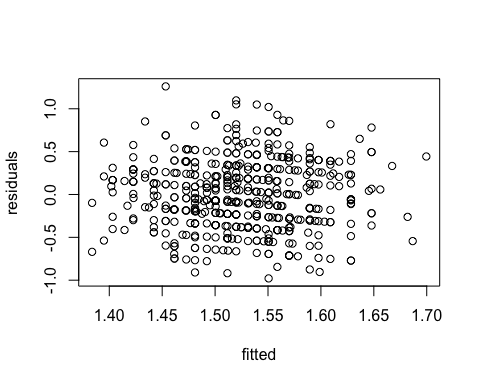

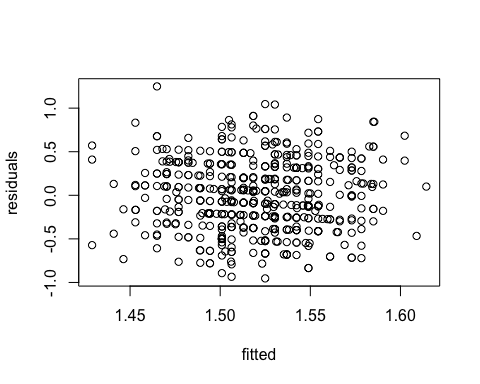

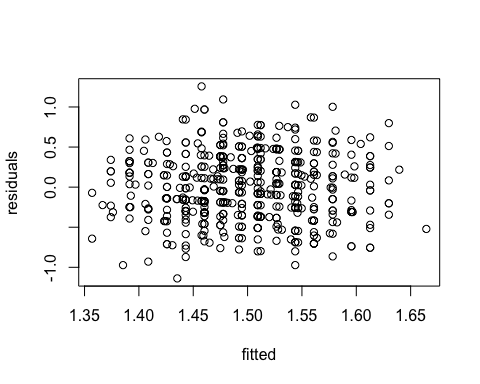

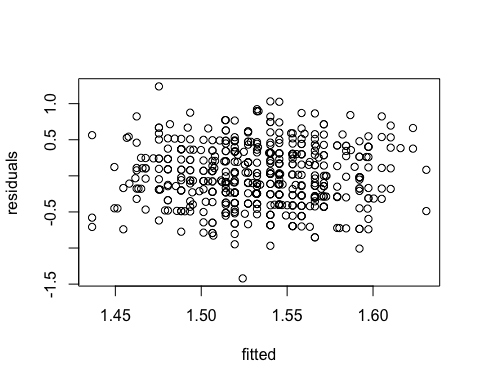

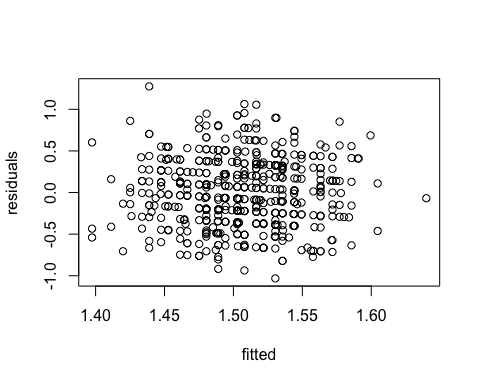

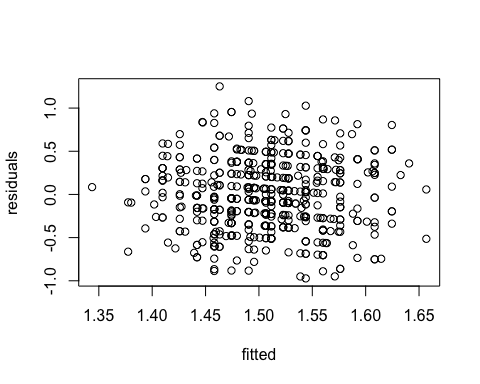

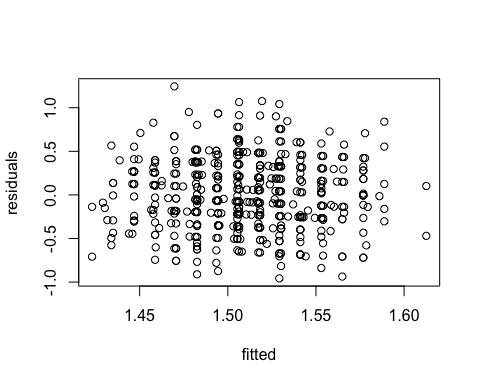

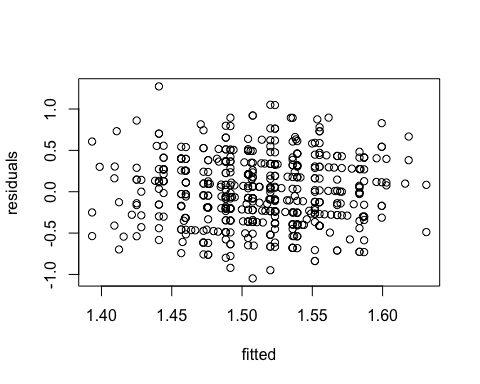

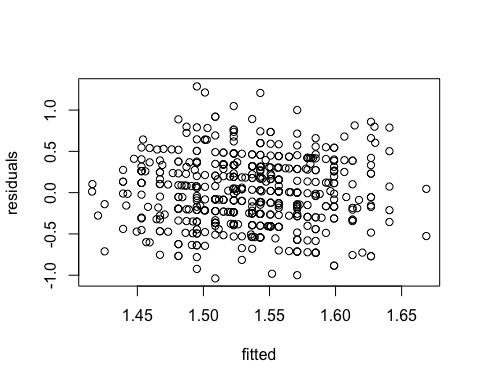

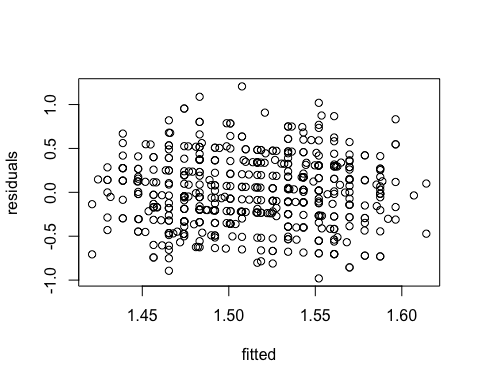


*## Checking normality using a qq plot*

*# Do it once*
**qqnorm**(gad_lms_no_summ_for_all_imps**$**imp1**$**residuals)
**qqline**(gad_lms_no_summ_for_all_imps**$**imp1**$**residuals) *#qq plot with line*

*# Do it for all imputations*

gad_norm_resid_for_all_imps <- **map**(gad_lms_no_summ_for_all_imps, **~**{
 **qqnorm**(.x**$**residuals)
 **qqline**(.x**$**residuals)
})


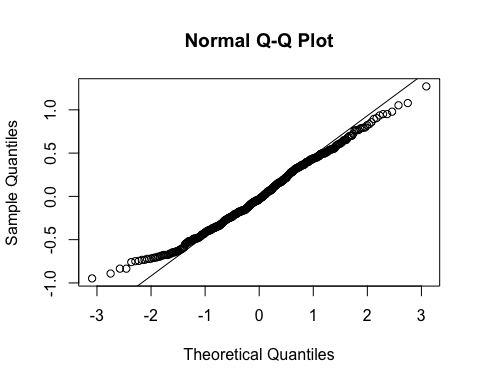

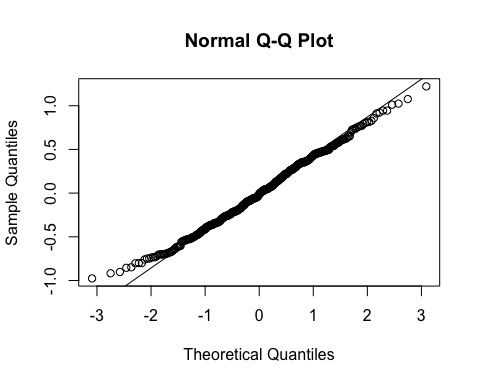

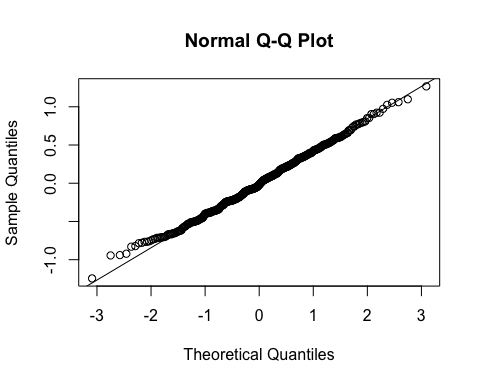

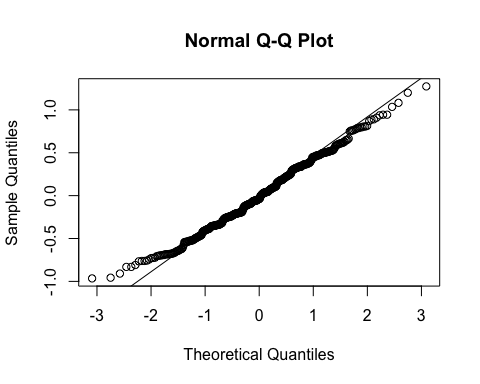

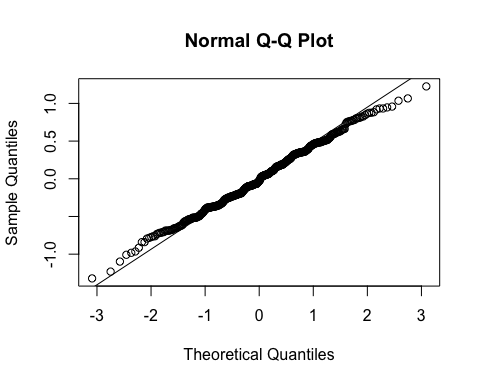

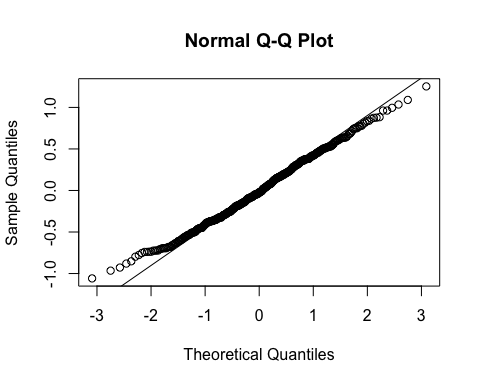

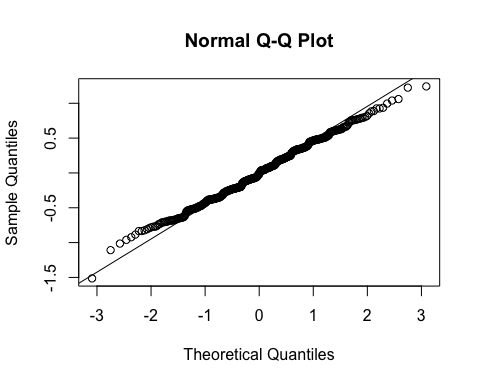

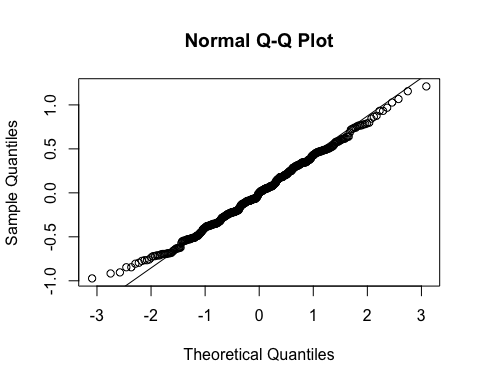

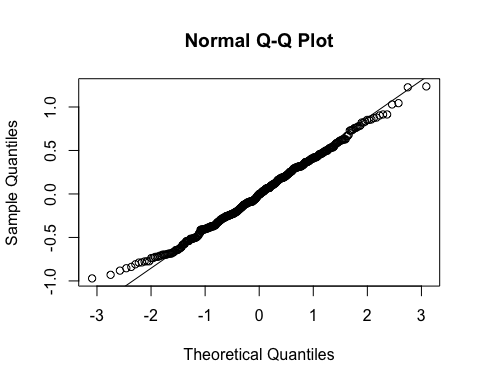

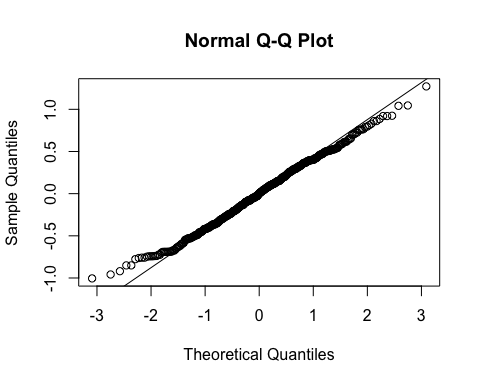

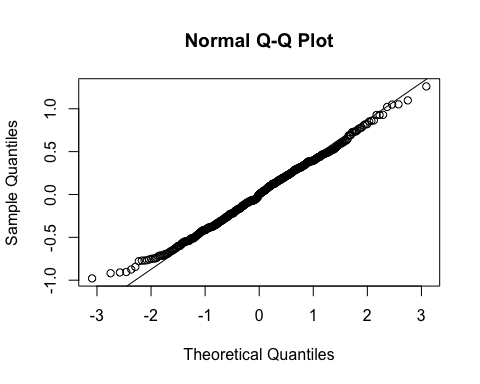

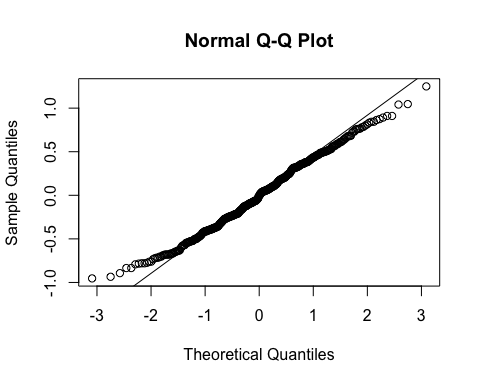

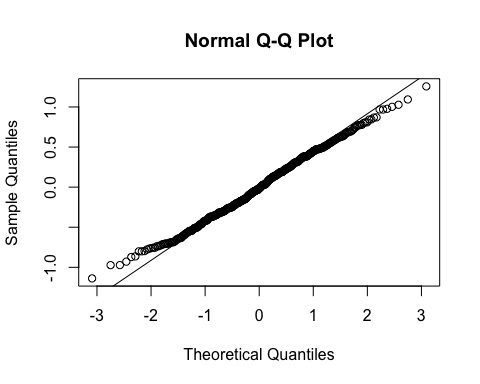

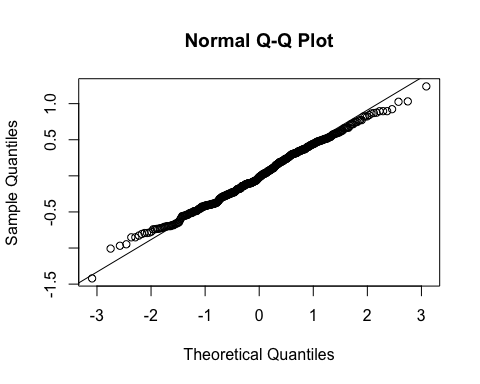

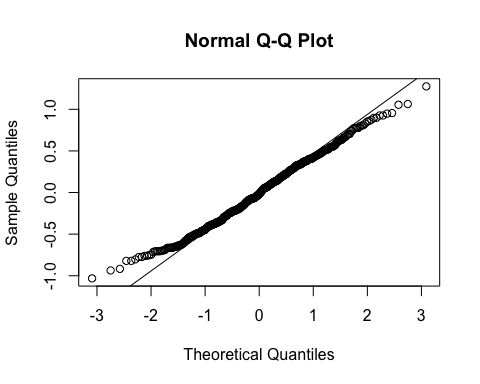

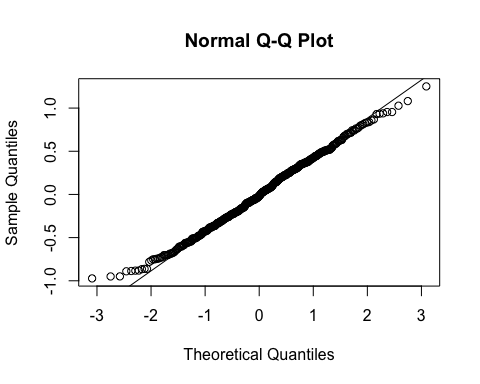

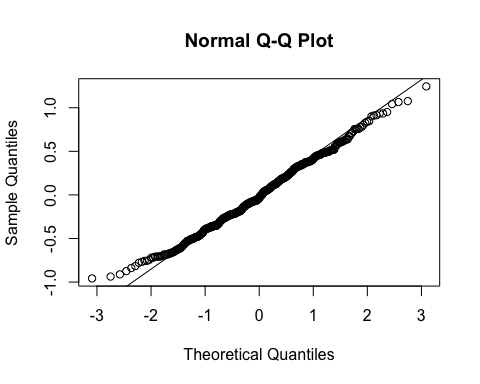

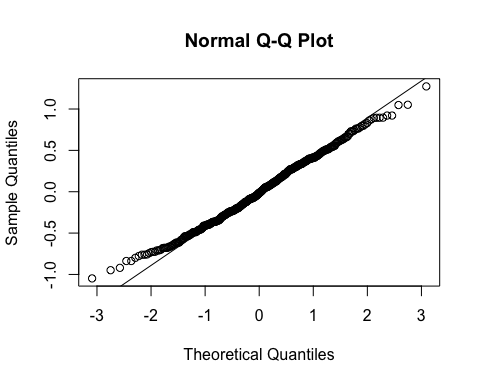

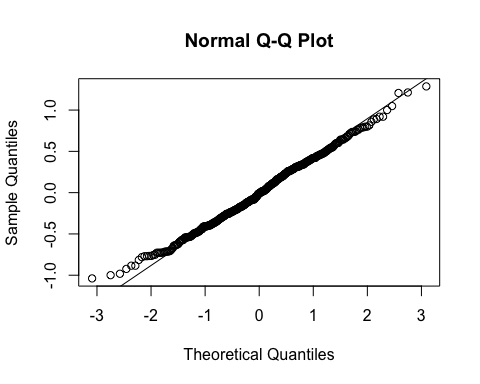

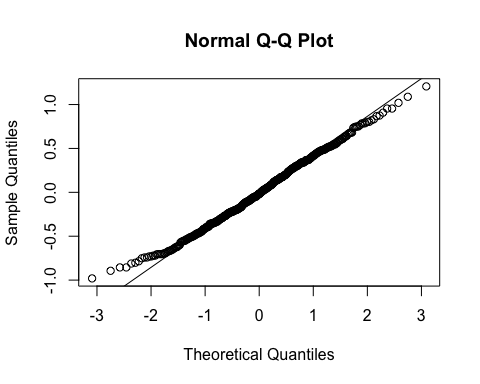


*## Checking multicollinearity using variance inflation factors (VIFs)*

*# Do it once*

car**::vif**(gad_lms_no_summ_for_all_imps**$**imp1)

## b_gad_7_mean cond
## 1.000749 1.000749

*# Do it for all imputations*

gad_vif_for_all_imps <- **map**(gad_lms_no_summ_for_all_imps, **~**{
 car**::vif**(.x)
})
gad_vif_for_all_imps

## $imp1
## b_gad_7_mean cond
## 1.000749 1.000749
##
## $imp2
## b_gad_7_mean cond
## 1.000118 1.000118
##
## $imp3
## b_gad_7_mean cond
## 1.001807 1.001807
##
## $imp4
## b_gad_7_mean cond
## 1.000372 1.000372
##
## $imp5
## b_gad_7_mean cond
## 1.00039 1.00039
##
## $imp6
## b_gad_7_mean cond
## 1.000004 1.000004
##
## $imp7
## b_gad_7_mean cond
## 1.001479 1.001479
##
## $imp8
## b_gad_7_mean cond
## 1.00017 1.00017
##
## $imp9
## b_gad_7_mean cond
## 1.000097 1.000097
##
## $imp10
## b_gad_7_mean cond
## 1.000912 1.000912
##
## $imp11
## b_gad_7_mean cond
## 1.001726 1.001726
##
## $imp12
## b_gad_7_mean cond
## 1.000002 1.000002
##
## $imp13
## b_gad_7_mean cond
## 1.000189 1.000189
##
## $imp14
## b_gad_7_mean cond
## 1.003055 1.003055
##
## $imp15
## b_gad_7_mean cond
## 1.001097 1.001097
##
## $imp16
## b_gad_7_mean cond
## 1.000084 1.000084
##
## $imp17
## b_gad_7_mean cond
## 1.000249 1.000249
##
## $imp18
## b_gad_7_mean cond
## 1.000004 1.000004
##
## $imp19
## b_gad_7_mean cond
## 1.000321 1.000321
##
## $imp20
## b_gad_7_mean cond
## 1.000373 1.000373

*## Checking for influential observations in the data*

*# Do it once*
cook <- **cooks.distance**(gad_lms_no_summ_for_all_imps**$**imp1)
**plot**(cook, ylab="Cook's distances")

*## Do it for all imputations*

gad_cook_dist_for_all_imps <- **map**(gad_lms_no_summ_for_all_imps, **~**{
 cook <- **cooks.distance**(.x)
 **plot**(cook, ylab="Cook's distances")
})


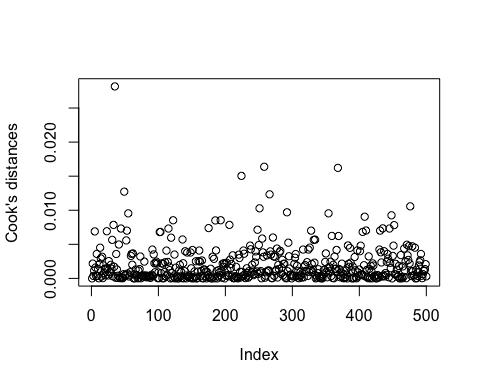

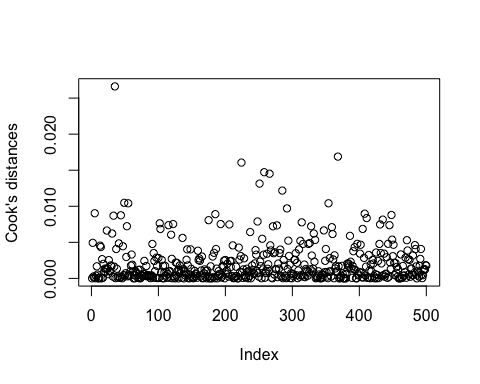

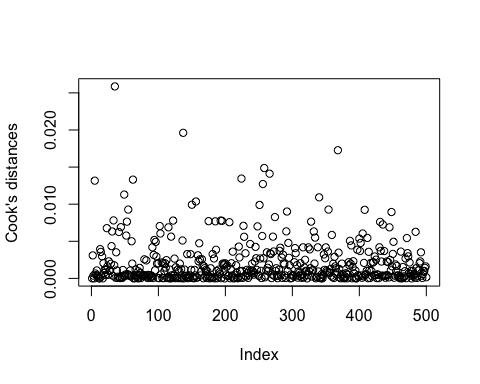

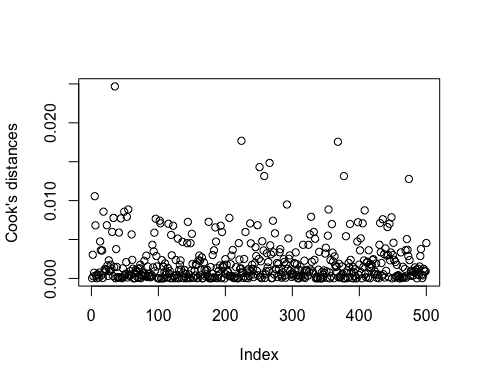

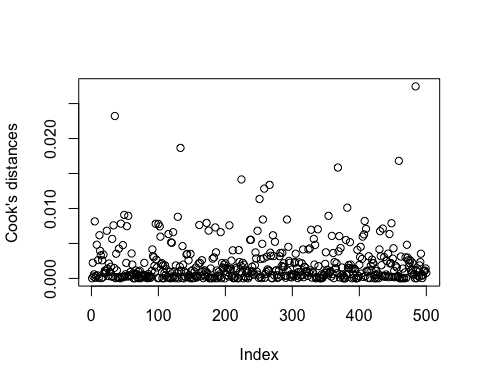

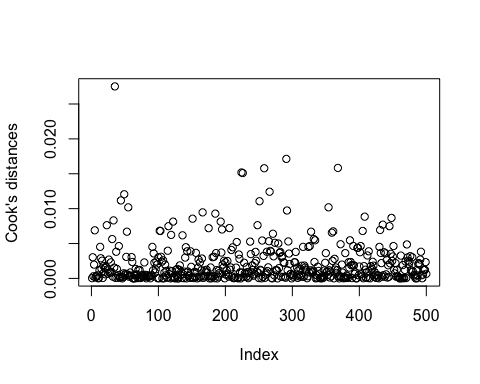

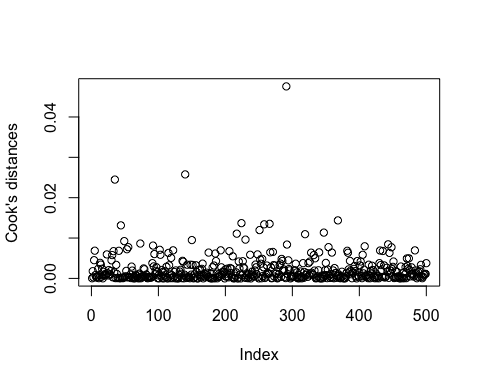

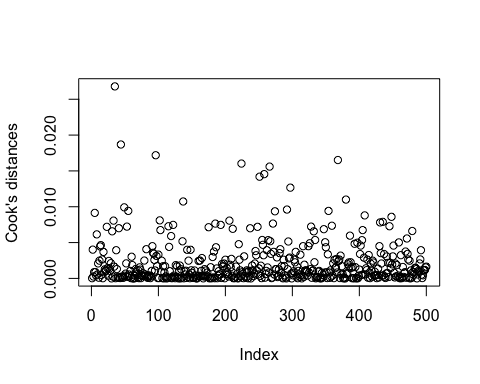

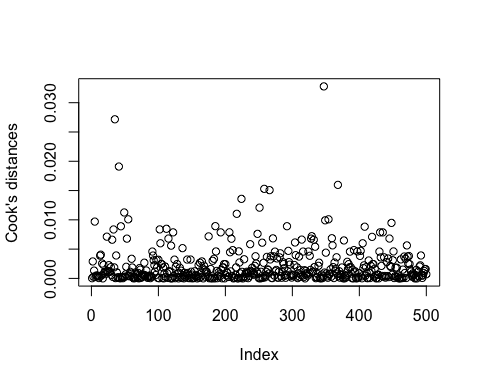

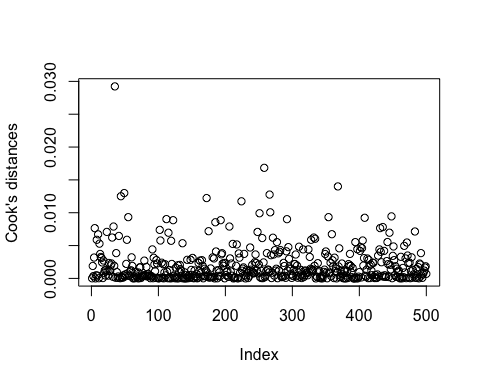

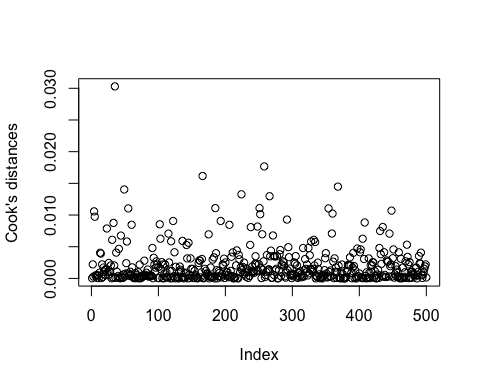

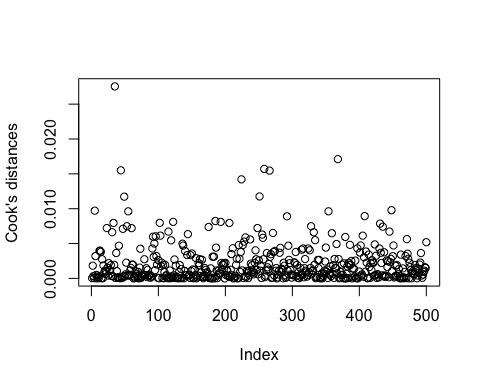

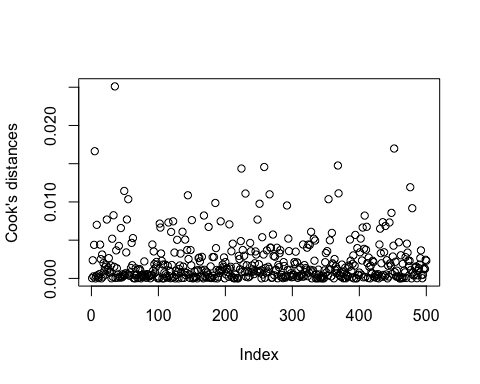

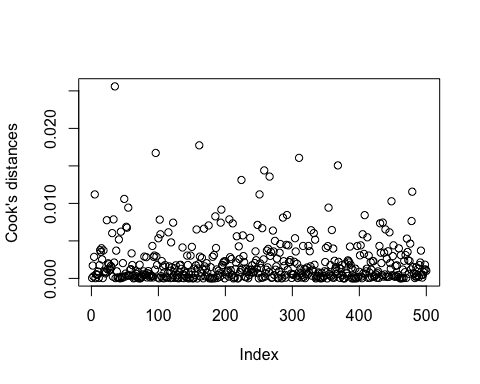

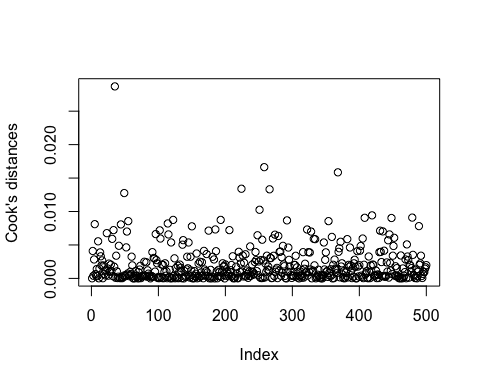

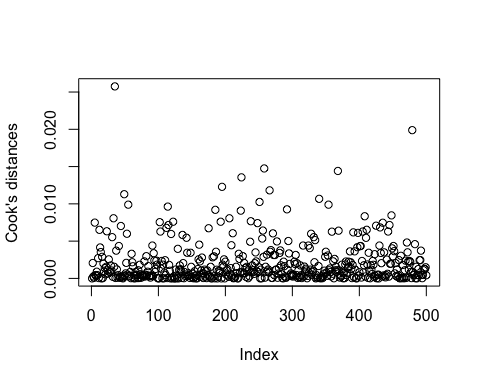

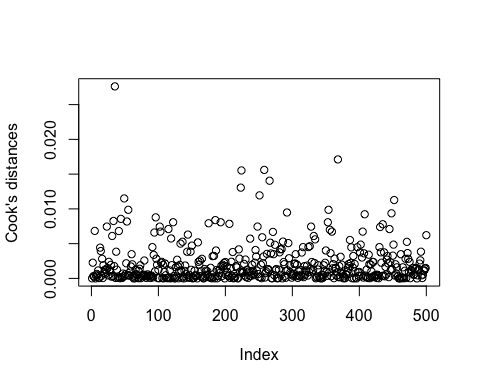

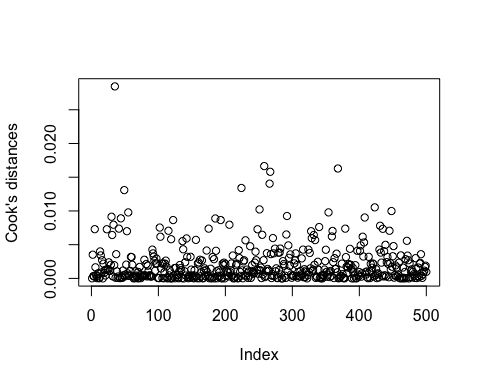

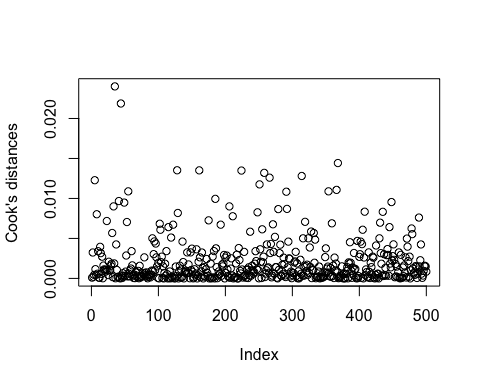

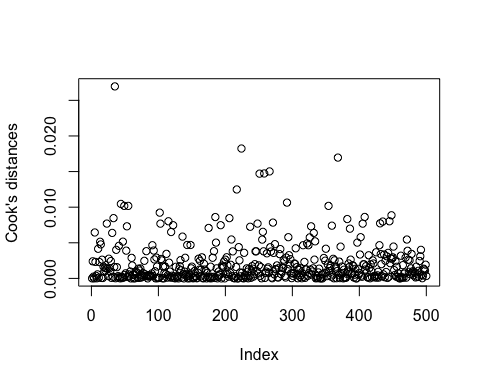


## **Data Transformation (If Necessary) in Response to Violations of Assumptions**

We will run all models first without these transformations, and then, as a sensitivity analysis, run the models with these transformations

*## If residuals appear positively skewed, we'll do a square root transformation of the dependent variable*

gad_out_imputations_with_gad_diff_asump_vio <- **map**(acq_out**$**imputations, **~**{
 .x **%>%**
 **mutate**(f_gad_7_mean_sqrt = **sqrt**(**abs**(f_gad_7_mean)))
})

*## If residuals appear negatively skewed, we'll do a square root transformation of the dependent variable following a scale reversal*

gad_out_imputations_with_acq_diff_asump_vio <- **map**(acq_out**$**imputations, **~**{
 .x **%>%**
 **mutate**(f_gad_7_mean_scale_reverse = dplyr**::recode**(f_gad_7_mean, '0' = 5, '1' = 4, '2' = 3, '3' = 2, '4' = 1, '5' = 0),
 f_gad_7_mean_scale_reverse_sqrt = **sqrt**(**abs**(f_gad_7_mean_scale_reverse)))
})

*## If there are outliers as defined by greater than the 95th % away, we'll Winsorize those values*

gad_out_imputations_with_acq_diff_asump_vio <- **map**(acq_out**$**imputations, **~**{
 .x **%>%**
 **mutate**(f_gad_7_mean_wins = **Winsorize**(f_gad_7_mean))
})

## **For The Simulated Data, We’d Fail to Reject the Null Hypothesis for Hypothesis 1**

*## Now let's aggregate across all our imputations to get our "average" outcome for testing alternative hypothesis for hypothesis 3*

*## Now need to run linear regressions on all the dataframes*
*## (We were using Zelig but: 1. It's a deprecated package and I can't figure out what it's doing/it's documentation is all over the place AND 2. Doing this here allows us to run similar code for all hypotheses/check this against the Zelig output if necessary 3. We need to do something like this anyway to check all assumptions)*

gad_lms_for_all_imps <- **map**(acq_out**$**imputations, **~**{
 **summary**(**lm**(f_gad_7_mean **~** b_gad_7_mean **+** cond, data = .x))
})

*## This gets a tidy version of estimate and standard error for all predictors + intercept for each regression*

gad_all_ests_for_all_imps_test <- **map**(gad_lms_for_all_imps, **~** **tidy**(.x))

*## Now I need to extract degrees of freedom for each linear regression (This has numerator and denominator)*
*## Haven't integrated this yet, since the df are the same across all imputed datasets. Can make this more funcitonal at some point in the future*

gad_df_for_all_imps <- **map**(gad_lms_for_all_imps, extract, **c**("df"))

*## Now have to extract specific estimate/standard error to eventually pass to the Amelia mi.meld function in order to aggregate the test statistics across imputed datasets according to Rubin's rules*

gad_int_order_ests_for_all_imps_test <- **map_df**(gad_all_ests_for_all_imps_test, **~**{
 .x **%>%**
 **filter**(term **==** "cond1") **%>%**
 dplyr**::select**(estimate,std.error)
}
)

*## Now pass those to the mi.meld function (Have to convert the columns/vectors to matrices to make this work)*

mi_avg_est_hyp_1_alt <- **mi.meld**(**as.matrix**(gad_int_order_ests_for_all_imps_test**$**estimate), **as.matrix**(gad_int_order_ests_for_all_imps_test**$**std.error), byrow = T)

*## This is the average estimate + the average standard error across imputeted datasets according to Rubin's rules*

mi_avg_est_hyp_1_alt**$**q.mi[1,1]

## [1] -0.02755698

mi_avg_est_hyp_1_alt**$**se.mi[1,1]

## [1] 0.04530468

*## Now let's calculate the t value by taking the esimate divided by the standard error*

mi_avg_t_value_hyp_1_alt <- mi_avg_est_hyp_1_alt**$**q.mi[1,1] **/** mi_avg_est_hyp_1_alt**$**se.mi[1,1]

*## And now the p value based on the just-calculated t value and degrees of freedom (Multiplied by 2 for a 2-tailed test) https://stats.stackexchange.com/questions/238427/why-the-lower-tail-f-is-used-when-mannualy-calculating-the-p-value-from-t-score*

p_value_hyp_1_alt <- 2***pt**(mi_avg_t_value_hyp_1_alt, df = 498, lower.tail = FALSE)
p_value_hyp_1_alt

## [1] 1.456707

*## Calculating d effect size with confidence intervals based on the t value using the MOTE package*
*## See also https://www.aggieerin.com/shiny-server/tests/indtt.html*
*## This effect size exactly matches what we were previously calculating from the Z-test from the Zelig package*

**d.ind.t.t**(mi_avg_t_value_hyp_1_alt, 250, 250, a = 0.0167) *# t value, n group 1, n group 2, alpha level*

## $d
## [1] -0.05451347
##
## $dlow
## [1] -0.2684753
##
## $dhigh
## [1] 0.1597212
##
## $n1
## [1] 250
##
## $n2
## [1] 250
##
## $df
## [1] 498
##
## $t
## [1] -0.608259
##
## $p
## [1] 0.543293
##
## $estimate
## [1] "$d_s$ = -0.05, 98.33\\% CI [-0.27, 0.16]"
##
## $statistic
## [1] "$t$ = (498) = -0.61, $p$ = .543"

## **Directly Testing the Null of Hypotheis 1**

In this case using randomly simulated data we get a p = .01 for our equivalence test of the null for hypothesis one, so we would intepret this evidence as in support of the null hypothesis for hypothesis 3: Contain COVID Anxiety was equivalent or inferior to the Remain COVID Free SSI at improving perceived control over anxiety.

*# Now, assuming the above is null, we directly test the null hypothesis for Hypothesis 1 (i.e. can we really say there is no difference between SSIs OR that the placebo is superior?)*

*# In order to apply Rubin's rule for aggregating estimates/standard errors across all imputed datasets, we need to have estimates and standard errors across all of those datasets. Unfortunately, the TOSTER package doesn't output the info we need to follow this process. So, we'll now re-create the equivalence test from scratch (Starting with the t-test, which is the test the equivalence test is based upon) to get the info we need.*

*#Start with running a t-test on every imputed dataset after regressing out baseline for the variable of interest (In this case general anxiety).*

*## Do it once*

acq_out**$**imputations**$**imp1**$**diff_score_res = **lm**(f_gad_7_mean **~** b_gad_7_mean **+** cond, data = acq_out**$**imputations**$**imp1)**$**residuals

t_test_example <- **t.test**(acq_out**$**imputations**$**imp1**$**diff_score_res **~** acq_out**$**imputations**$**imp1**$**cond, var.equal = T)

t_test_example**$**parameter *# degrees of freedom*

## df
## 498

t_test_example**$**estimate *# Estimates for both groups*

## mean in group 0 mean in group 1
## -2.063224e-17 -2.390143e-17

t_test_example**$**stderr *# Standard error*

## [1] 0.03653921

*## Do it for all imputations*

*# First run a t-test on all dataframes and save the values*

gad_t_tests_hyp_1_for_all_imps <- **map**(acq_out**$**imputations, **~**{
 .x**$**diff_score_res = **lm**(f_gad_7_mean **~** b_gad_7_mean **+** cond, data = .x)**$**residuals
 t_test_example <- **t.test**(.x**$**diff_score_res **~** .x**$**cond, var.equal = T)
})

*# Then extract the values we need (Using the tidy argument omits standard error)*

gad_t_tests_tidy_hyp_1_for_all_imps <- **map**(gad_t_tests_hyp_1_for_all_imps, **~** **tidy**(.x))

*# Using the tidy argument omits standard error from its output but we can calculate it based on the t ratio and the difference between the two sample means. T value statistic = estimate / standard error -> standard error = estimate / T value statistic. The standard error for imputation 1 using this method also matches when we calculate it manually above. We also map this to a dataframe rather than a list so we can extract the values easily in the next step.*

gad_t_tests_tidy_std_err_hyp_1_for_all_imps <- **map_df**(gad_t_tests_tidy_hyp_1_for_all_imps, **~**{
 .x **%>%**
 **mutate**(estimate = estimate2 **-** estimate1,
 std.error = **abs**(estimate **/** statistic))
})

*## Now have to put estimate and standard error into the mi.meld function to apply Rubin's rules for aggregating across multiple imputed datasets (Have to convert the columns/vectors to matrices to make this work)*

mi_avg_est_hyp_1_null <- **mi.meld**(**as.matrix**(gad_t_tests_tidy_std_err_hyp_1_for_all_imps**$**estimate), **as.matrix**(gad_t_tests_tidy_std_err_hyp_1_for_all_imps**$**std.error), byrow = T)

*## This is the average estimate + the average standard error across imputeted datasets according to Rubin's rules*

mi_avg_est_hyp_1_null**$**q.mi[1,1]

## [1] 6.838638e-18

mi_avg_est_hyp_1_null**$**se.mi[1,1]

## [1] 0.03675401

*# But we also need the pooled standard deviation to give the correct bounds for the equivalence test, since we have to multiply the d values of interest (defined ahead of time) by the pooled standard deviation to get the equivalence bounds we need in this circumstance*

*# Calculating pooled standard deviation from average standard error using this formula from Cochrane: https://handbook-5-1.cochrane.org/chapter_7/7_7_3_3_obtaining_standard_deviations_from_standard_errors.htm*

pooled_sd_hyp_1_null <- (mi_avg_est_hyp_1_null**$**se.mi[1,1])**/sqrt**(((1**/**250) **+** (1**/**250)))

*# Now create equivlanece bounds in scale units rather than d effect size units*

low_eqbound <- -0.33 ***** pooled_sd_hyp_1_null
high_eqbound <- 0.33 ***** pooled_sd_hyp_1_null

*# Let's create a lower bound t value*

t_low <- (mi_avg_est_hyp_1_null**$**q.mi[1,1] **+** low_eqbound)**/**mi_avg_est_hyp_1_null**$**se.mi[1,1]

*# And an upper bound t value*

t_high <- (mi_avg_est_hyp_1_null**$**q.mi[1,1] **+** high_eqbound)**/**mi_avg_est_hyp_1_null**$**se.mi[1,1]

*# Then compute p values for both*

p1 <- **pt**(t_low, 498, lower.tail = TRUE) *# P value for the low test, i.e. is the effect reliably greater than*
*## d = -0.63 (favoring the placebo)*
p2 <- **pt**(t_high, 498, lower.tail = FALSE) *# P vlue for the high test, i.e. is the effect reliably less than*
*## d = 0.21 (smallest effect size of interest favoring the intervention)*

*# And take the higher of those two as the final p value for the equivalence test*

**max**(p1, p2)

## [1] 0.0001247215

## **Conducting a Unit Test to Validate Our From Scratch Equivalence Testing Procedure**

This is a unit test to validate that our method and the TOSTER package return the same results when applied to the same dataset. We focus on the first imputed dataset for this test, and our version returns the same result as the TOSTER test. Please see the in-chunk comments for more detail.

*## Let's unit test this by confirming with the equivalence test package to make sure my method gets the same answer on the same dataset. We'll use imp1 as we've been using that as our test throughout*

df_equiv <- acq_out**$**imputations**$**imp1 **%>%**
 **mutate**(
 diff_score_res = **lm**(f_gad_7_mean **~** b_gad_7_mean)**$**residuals *#creating a dataframe with residuals for ACQ residual scores*
 ) **%>%**
 dplyr**::group_by**(cond) **%>%** *#grouping by SSI condition*
 **summarise**(
 mean_diff_score_res = **mean**(diff_score_res), *#we'll be looking at means and sds in each group*
 sd_diff_score_res = **sd**(diff_score_res)
 )

**TOSTtwo**(m1 = df_equiv[[1,2]], m2 = df_equiv[[2,2]], sd1 = df_equiv[[1,3]], sd2 = df_equiv[[2,3]],
 n1 = 250, n2 = 250, low_eqbound_d = -0.33, high_eqbound_d = 0.33, alpha = 0.0167,
 var.equal = TRUE, plot = FALSE, verbose = TRUE)

## TOST results:
## t-value lower bound: 4.40 p-value lower bound: 0.000007
## t-value upper bound: -2.98 p-value upper bound: 0.002
## degrees of freedom : 498
##
## Equivalence bounds (Cohen's d):
## low eqbound: -0.33
## high eqbound: 0.33
##
## Equivalence bounds (raw scores):
## low eqbound: -0.1348
## high eqbound: 0.1348
##
## TOST confidence interval:
## lower bound 96.66% CI: -0.052
## upper bound 96.66% CI: 0.104
##
## NHST confidence interval:
## lower bound 98.33% CI: -0.062
## upper bound 98.33% CI: 0.114
##
## Equivalence Test Result:
## The equivalence test was significant, t(498) = -2.980, p = 0.00151, given equivalence bounds of -0.135 and 0.135 (on a raw scale) and an alpha of 0.0167.
## Null Hypothesis Test Result:
## The null hypothesis test was non-significant, t(498) = 0.710, p = 0.478, given an alpha of 0.0167.
## Based on the equivalence test and the null-hypothesis test combined, we can conclude that the observed effect is statistically not different from zero and statistically equivalent to zero.

*## Now back to our method*

acq_out**$**imputations**$**imp1**$**diff_score_res = **lm**(f_gad_7_mean **~** b_gad_7_mean, data = acq_out**$**imputations**$**imp1)**$**residuals

t_test_example <- **t.test**(acq_out**$**imputations**$**imp1**$**diff_score_res **~** acq_out**$**imputations**$**imp1**$**cond, var.equal = T)

*## We're getting the same t-value and p-value as the t-test conducted above (Not the equivalence test yet)*

tidy_t_test_example <- **tidy**(t_test_example)

*## How do the estimates match up? They match exactly*

tidy_t_test_example_w_estimate <- tidy_t_test_example **%>%**
 **mutate**(estimate = estimate2 **-** estimate1,
 std.error = **abs**(estimate **/** statistic))

df_equiv[[1,2]]

## [1] 0.01291746

tidy_t_test_example_w_estimate**$**estimate1

## [1] 0.01291746

df_equiv[[2,2]]

## [1] -0.01302121

tidy_t_test_example_w_estimate**$**estimate2

## [1] -0.01302121

pooled_sd_hyp_1_null_t_test_example <- (tidy_t_test_example_w_estimate**$**std.error)**/sqrt**(((1**/**250) **+** (1**/**250)))

*# Now create equivlanece bounds in scale units rather than d effect size units*
*## These are exactly the same as above at the thousandths decimal place.*

low_eqbound_t_test_example <- -0.33 ***** pooled_sd_hyp_1_null_t_test_example
high_eqbound_t_test_example <- 0.33 ***** pooled_sd_hyp_1_null_t_test_example

*# Let's create a lower bound t value*

*# To get matching results with the TOSTER package we need to add the equivalence bounds to the estimate values . Once we do this we get the exact same t values as the TOSTER package.*

t_low_t_test_example <- (tidy_t_test_example_w_estimate**$**estimate **+** low_eqbound_t_test_example)**/**tidy_t_test_example_w_estimate**$**std.error

*# And an upper bound t value*

t_high_t_test_example <- (tidy_t_test_example_w_estimate**$**estimate **+** high_eqbound_t_test_example)**/**tidy_t_test_example_w_estimate**$**std.error

*# Then compute p values for both*

*# In order to match the p value from our output to the TOSTER package output, we need to have lower.tail = TRUE for the lower bound example and lower.tail = FALSE for the high bound example.*

p1 <- **pt**(t_low_t_test_example, 498, lower.tail = TRUE) *# P value for the low test, i.e. is the effect reliably greater than*
*## d = -0.63 (favoring the placebo)*
p2 <- **pt**(t_high_t_test_example, 498, lower.tail = FALSE)

**max**(p1, p2)

## [1] 0.00151348

## **Can We Be Sure Our SSIs Aren’t Negatively Impacting Social Distancing Intentions?**

Testing hypothesis 2 using a paired-equivalence test (including baseline & follow-up social distancing scores, with equivalence bounds; testing both null and alternative hypotheses using the equivalence test here). I.e. can we say that the SSIs did not decrease (or increase) social distancing willingness/intentions?

## **Directly Testing the Alternative Hypothesis for Hypothesis 2**

Or, can we say that the effect of viewing both interventions is statistically equivalent to zero using a paired equivalence test? In this simulated data we reject the null hypothesis that the social distancing intentions do not differ post both SSIs compared to pre both SSIs (p < .001). Therefore, the evidence supports the alternative hypothesis that the pre to post both SSIs effect on social distancing intentions is 0.

*# In order to apply Rubin's rule for aggregating estimates/standard errors across all imputed datasets, we need to have estimates and standard errors across all of those datasets. Unfortunately, the TOSTER package doesn't output the info we need to follow this process. So, we'll now re-create the equivalence test from scratch (Starting with the t-test, which is the test the equivalence test is based upon) to get the info we need.*

*#Start with running a paired t-test on every imputed dataset after regressing out baseline for the variable of interest (In this case perceived control over anxiety). Note: I think, as was pointed out by Mallory, that we could probably literally use the same estimates and standard errors calculated above in this case, since the t-test of the diff_acq_mean after residualizing the b_acq_mean should be equivalent to the estimates we get from the regression above. But I've included this code both to be explicit about what we're doing and guard against any potential idiosyncratic differences between those estimates I might not know about.*

*## Do it once*

df_equiv_hyp_2_alt <- soc_dist_out**$**imputations**$**imp1 **%>%**
 **filter**(cond **==** 1)

t_test_hyp_2_example <- **t.test**(df_equiv_hyp_2_alt**$**b_soc_dist_mean, df_equiv_hyp_2_alt**$**pi_soc_dist_mean, var.equal = T, paired = TRUE)

t_test_hyp_2_example**$**parameter *# degrees of freedom*

## df
## 240

t_test_hyp_2_example**$**estimate *# Single estimate here, which will allow us to simplify the code a bit later*

## mean of the differences
## -0.01470568

t_test_hyp_2_example**$**stderr *# Standard error*

## [1] 0.05415632

*## Do it for all imputations*

*# First run a t-test on all dataframes and save the values*

soc_dist_t_tests_hyp_2_for_all_imps <- **map**(soc_dist_out**$**imputations, **~**{
 df_equiv_hyp_2_alt <- .x **%>%**
 **filter**(cond **==** 1)
 **t.test**(df_equiv_hyp_2_alt**$**b_soc_dist_mean, df_equiv_hyp_2_alt**$**pi_soc_dist_mean, var.equal = T, paired = TRUE)
})

*# Then extract the values we need (Using the tidy argument omits standard error)*

soc_dist_t_tests_tidy_hyp_2_for_all_imps <- **map**(soc_dist_t_tests_hyp_2_for_all_imps, **~** **tidy**(.x))

*# Using the tidy argument omits standard error from its output but we can calculate it based on the t ratio and the difference between the two sample means. T value statistic = estimate / standard error -> standard error = estimate / T value statistic. The standard error for imputation 1 using this method also matches when we calculate it manually above. We also map this to a dataframerather than a list so we can extract the values easily in the next step.*

soc_dist_t_tests_tidy_std_err_hyp_2_for_all_imps <- **map_df**(soc_dist_t_tests_tidy_hyp_2_for_all_imps, **~**{
 .x **%>%**
 **mutate**(
 std.error = **abs**(estimate **/** statistic))
})

*## Now have to put estimate and standard error into the mi.meld function to apply Rubin's rules for aggregating across multiple imputed datasets (Have to convert the columns/vectors to matrices to make this work)*

mi_avg_est_hyp_2_altern <- **mi.meld**(**as.matrix**(soc_dist_t_tests_tidy_std_err_hyp_2_for_all_imps**$**estimate), **as.matrix**(soc_dist_t_tests_tidy_std_err_hyp_2_for_all_imps**$**std.error), byrow = T)

*## This is the average estimate + the average standard error across imputeted datasets according to Rubin's rules*

mi_avg_est_hyp_2_altern**$**q.mi[1,1]

## [1] 0.00382091

mi_avg_est_hyp_2_altern**$**se.mi[1,1]

## [1] 0.07334538

*# But we also need the pooled standard deviation to give the correct bounds for the equivalence test, since we have to multiply the d values of interest (defined ahead of time) by the pooled standard deviation to get the equivalence bounds we need in this circumstance*

*# Calculating pooled standard deviation from average standard error using this formula from Cochrane: https://handbook-5-1.cochrane.org/chapter_7/7_7_3_2_obtaining_standard_deviations_from_standard_errors_and.htm*

pooled_sd_hyp_2_altern <- (mi_avg_est_hyp_2_altern**$**se.mi[1,1]) ***** **sqrt**(250)

*# Now create equivlanece bounds in scale units rather than d effect size units*

low_eqbound_hyp_2_altern <- -0.33 ***** pooled_sd_hyp_2_altern
high_eqbound_hyp_2_altern <- 0.33 ***** pooled_sd_hyp_2_altern

*# Let's create a lower bound t value*

t_low_hyp_2_altern <- (mi_avg_est_hyp_2_altern**$**q.mi[1,1] **+** low_eqbound_hyp_2_altern)**/**mi_avg_est_hyp_2_altern**$**se.mi[1,1]

*# And an upper bound t value*

t_high_hyp_2_altern <- (mi_avg_est_hyp_2_altern**$**q.mi[1,1] **+** high_eqbound_hyp_2_altern)**/**mi_avg_est_hyp_2_altern**$**se.mi[1,1]

*# Then compute p values for both*

p1_hyp_2_altern <- **pt**(t_low_hyp_2_altern, 249, lower.tail = TRUE) *# P value for the low test, i.e. is the effect reliably greater than*
*## d = -0.21 (favoring a decrease in social distancing intentions)*
p2_hyp_2_altern <- **pt**(t_high_hyp_2_altern, 249, lower.tail = FALSE) *# P vlue for the high test, i.e. is the effect reliably less than*
*## d = 0.21 (favoring an increase in social distancing intentions)*

*# And take the higher of those two as the final p value for the equivalence test*

**max**(p1_hyp_2_altern, p2_hyp_2_altern)

## [1] 2.455521e-07

## **Directly Testing the Null of Hypothesis 2**

Or whether social distancing intentions either increased or decreased pre to post both SSIs. In this case we get a p-value of .94. (This test would not have been conducted on this data per our pre-specified analytic plan, given the significant p value for the test of the alternative hypothesis for hypothesis 2, but is included here so reviewers can see how we would conduct the direct test of the null for hypothesis 2).

*## Now let's calculate the t value for the paired t-test by taking the esimate divided by the standard error*

mi_avg_t_value_hyp_2_null <- mi_avg_est_hyp_2_altern**$**q.mi[1,1] **/** mi_avg_est_hyp_2_altern**$**se.mi[1,1]

*## And now the p value based on the just-calculated t value and degrees of freedom. Multiplying the value by 2 to make it a 2-tailed test*

p_value_hyp_2_null <- 2***pt**(mi_avg_t_value_hyp_2_null, df = 249, lower.tail = T)
p_value_hyp_2_null

## [1] 1.041505

*## Calculating d_zeffect size with confidence intervals based on the t value using the MOTE package*
*## See also https://www.aggieerin.com/shiny-server/tests/deptdifft.html*

**d.dep.t.diff.t**(mi_avg_t_value_hyp_2_null, 250, a = 0.05) *# t value, sample size, alpha level*

## $d
## [1] 0.003294761
##
## $dlow
## [1] -0.1206679
##
## $dhigh
## [1] 0.1272508
##
## $n
## [1] 250
##
## $df
## [1] 249
##
## $t
## [1] 0.05209475
##
## $p
## [1] 0.958495
##
## $estimate
## [1] "$d_z$ = 0.00, 95\\% CI [-0.12, 0.13]"
##
## $statistic
## [1] "$t$(249) = 0.05, $p$ = .958"

## **Does the COVID Anxiety SSI Increase ACQ Scores More Than the Control? Directly Testing the Alternative Hypothesis of Hypothesis 3**

Testing hypothesis 3 using a linear regression. We first create a list all of the multiply imputed datasets.

*# Amelia creates a list of dataframes equal to our number of imputations, which we can pass to the first argument of the 'map' function in the purrr package*

**class**(acq_out**$**imputations)

## [1] "mi" "list"

*## A template for basic map() usage: map(YOUR_LIST, YOUR_FUNCTION)*
*## This allows us to return a list of dataframes (using map) that's flexible across number of imputations.*

*## See https://community.rstudio.com/t/simplest-way-to-modify-the-same-column-in-multiple-dataframes-in-a-list/13076 for code I modified to make this happen*

*## This tutorial for map was also helpful! https://jennybc.github.io/purrr-tutorial/ls01_map-name-position-shortcuts.html#load_packages*
*## Branched off of these resources https://jennybc.github.io/purrr-tutorial/*

## **Assumption Checks for Hypothesis 3**

These assumption checks are all visual in nature without hard cut-offs. Therefore, if we choose to conduct any data transformations we will present both the non-transformed results and the transformed results (along with the results of all assumption checks).

*# Checking assumptions in imputed data*

*# First have to run the linear model of interest on all imputed datasets*

acq_lms_no_summ_for_all_imps <- **map**(acq_out**$**imputations, **~**{
 **lm**(pi_acq_mean **~** b_acq_mean **+** cond, data = .x)
})

*## Checking linearity & homoscedasticity using residuals vs. predicted values plot*

*# Do it once*

**plot**(acq_lms_no_summ_for_all_imps**$**imp1**$**fitted.values, acq_lms_no_summ_for_all_imps**$**imp1**$**residuals)


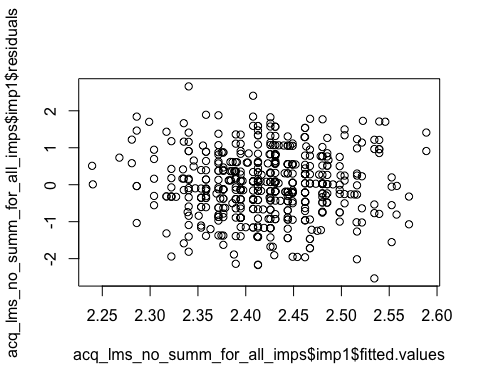


*# Do it for all imputations HAVE TO CALL THE WHOLE THING, NOT JUST THE VARIABLE*

acq_linearity_scedast_for_all_imps <- **map**(acq_out**$**imputations, **~**{
 fitted <- **lm**(pi_acq_mean **~** b_acq_mean **+** cond, data = .x)**$**fitted.values
 residuals <- **lm**(pi_acq_mean **~** b_acq_mean **+** cond, data = .x)**$**residuals
 **plot**(fitted,residuals)
})


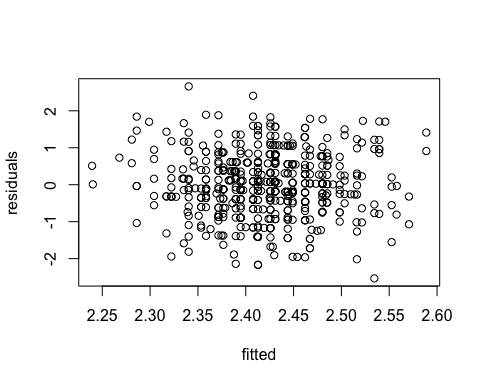

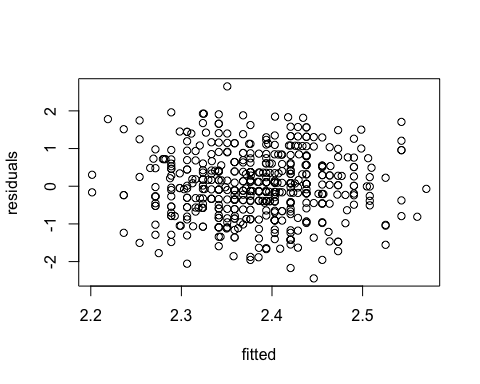

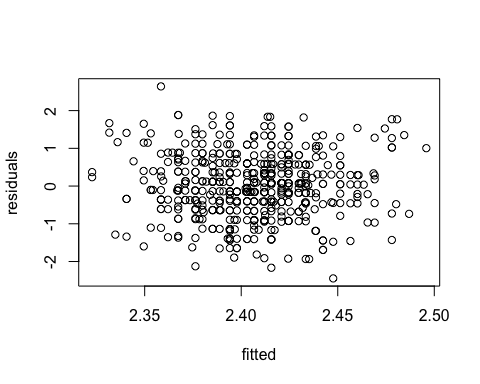

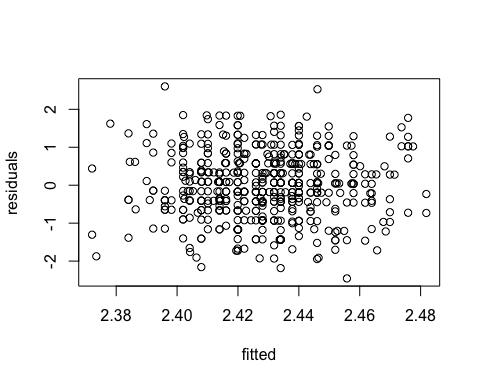

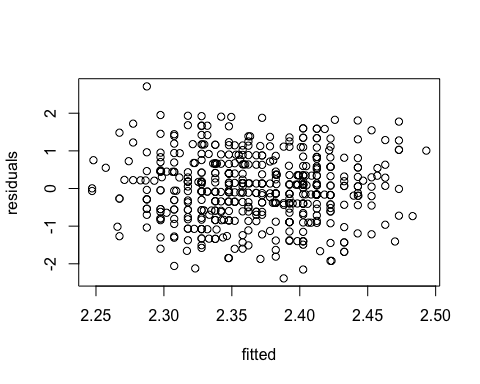

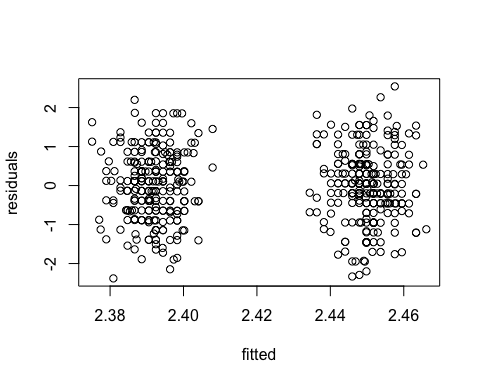

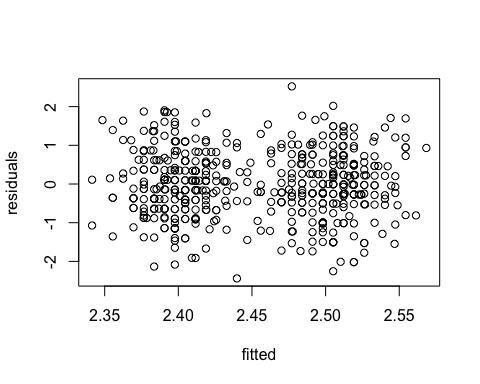

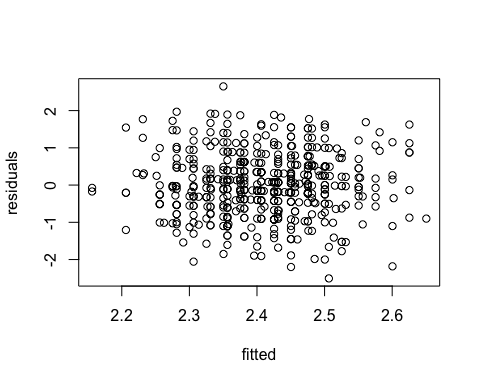

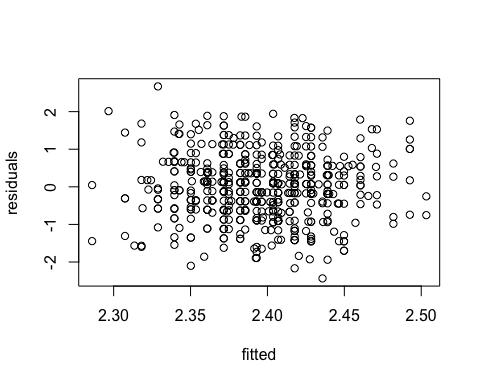

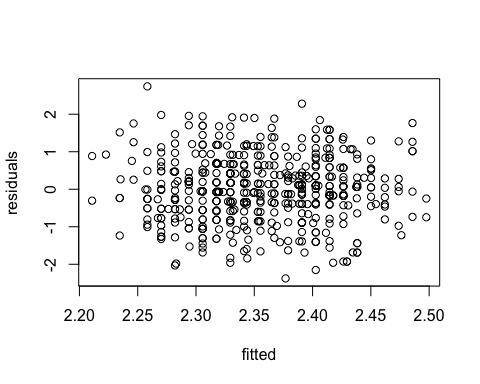

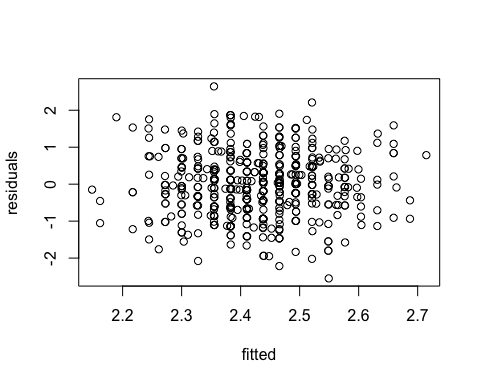

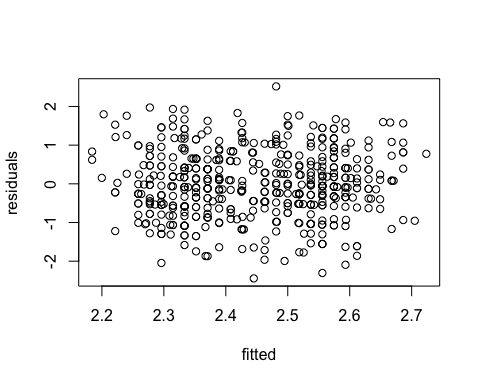

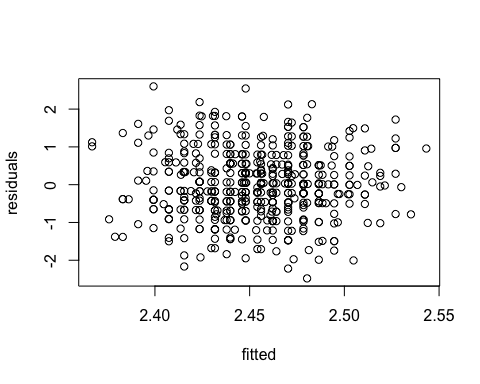

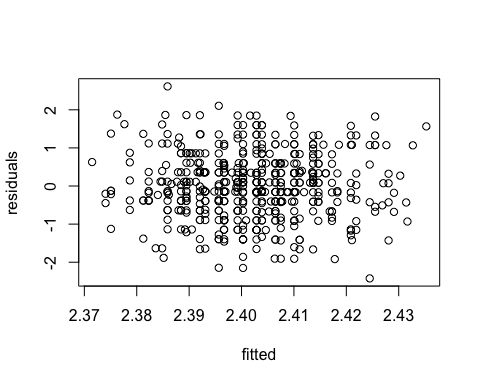

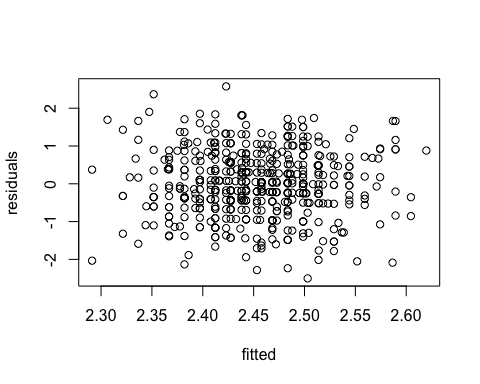

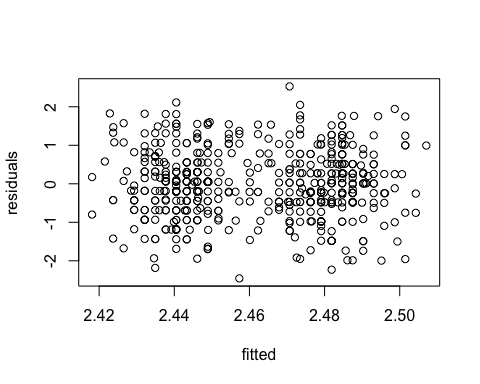

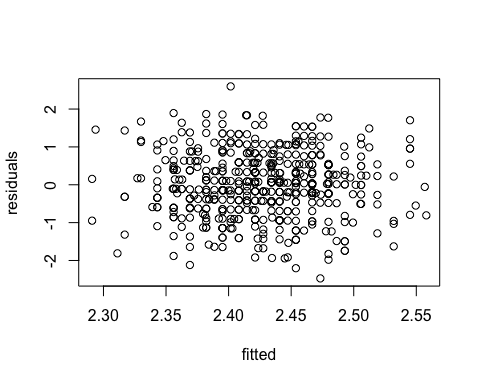

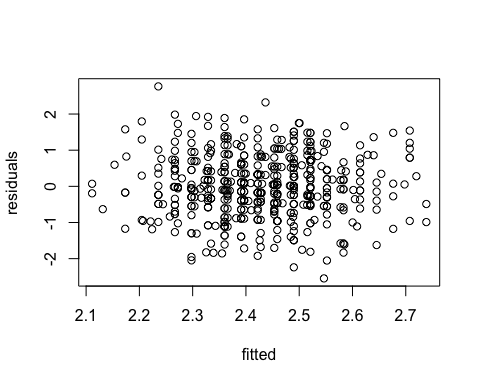

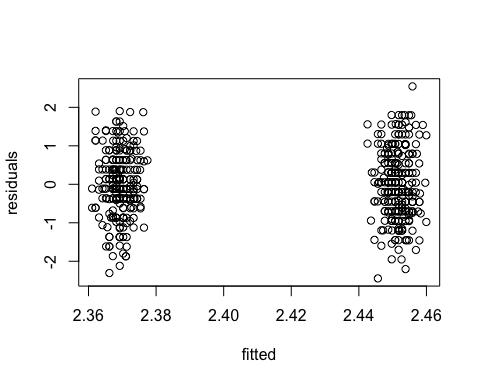

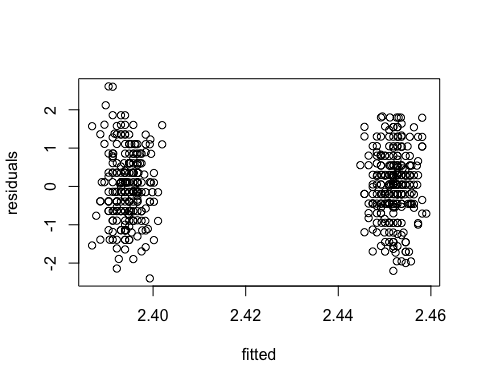


*## Checking normality using a qq plot*

*# Do it once*
**qqnorm**(acq_lms_no_summ_for_all_imps**$**imp1**$**residuals)
**qqline**(acq_lms_no_summ_for_all_imps**$**imp1**$**residuals) *#qq plot with line*

*# Do it for all imputations*

acq_norm_resid_for_all_imps <- **map**(acq_lms_no_summ_for_all_imps, **~**{
 **qqnorm**(.x**$**residuals)
 **qqline**(.x**$**residuals)
})


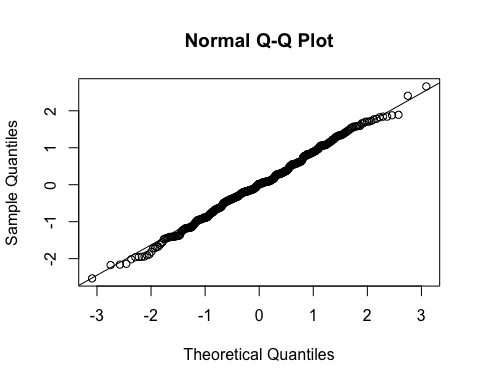

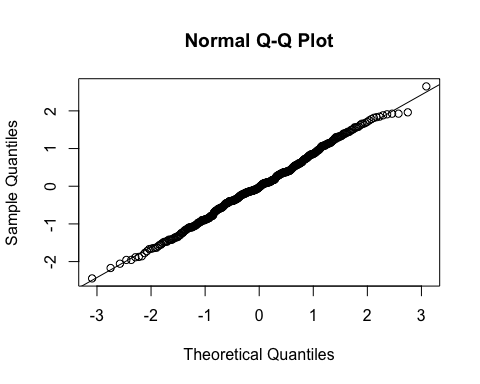

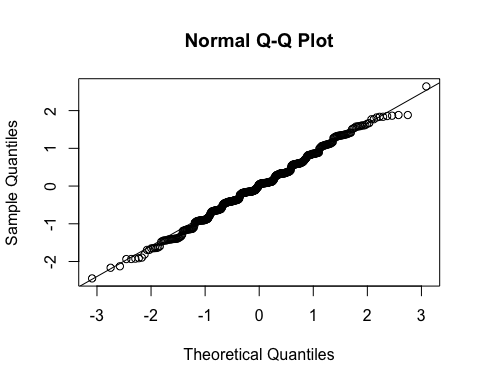

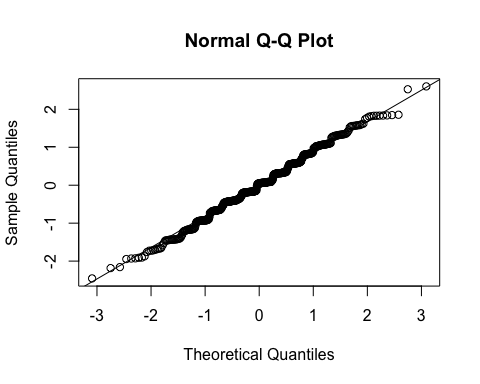

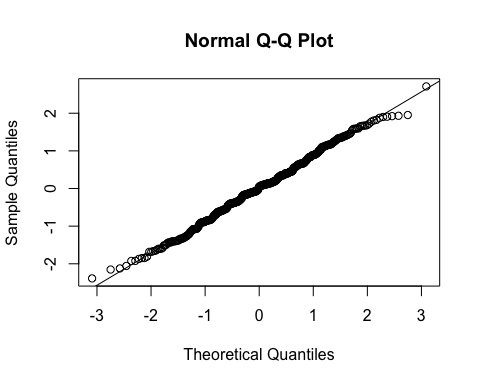

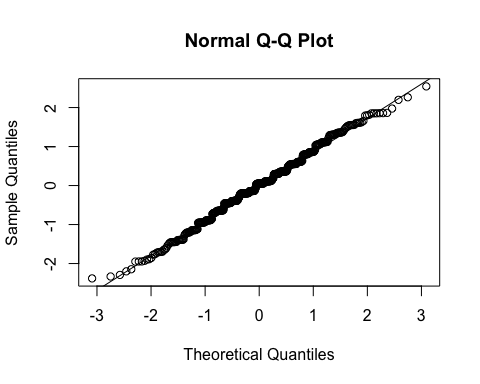


*## Checking multicollinearity using variance inflation factors (VIFs)*

*# Do it once*

car**::vif**(acq_lms_no_summ_for_all_imps**$**imp1)

## b_acq_mean cond
## 1.005776 1.005776

*# Do it for all imputations*

acq_vif_for_all_imps <- **map**(acq_lms_no_summ_for_all_imps, **~**{
 car**::vif**(.x)
})
acq_vif_for_all_imps

## $imp1
## b_acq_mean cond
## 1.005776 1.005776
##
## $imp2
## b_acq_mean cond
## 1.015931 1.015931
##
## $imp3
## b_acq_mean cond
## 1.002839 1.002839
##
## $imp4
## b_acq_mean cond
## 1.006399 1.006399
##
## $imp5
## b_acq_mean cond
## 1.002915 1.002915
##
## $imp6
## b_acq_mean cond
## 1.008841 1.008841
##
## $imp7
## b_acq_mean cond
## 1.005739 1.005739
##
## $imp8
## b_acq_mean cond
## 1.015048 1.015048
##
## $imp9
## b_acq_mean cond
## 1.01426 1.01426
##
## $imp10
## b_acq_mean cond
## 1.007849 1.007849
##
## $imp11
## b_acq_mean cond
## 1.002727 1.002727
##
## $imp12
## b_acq_mean cond
## 1.009615 1.009615
##
## $imp13
## b_acq_mean cond
## 1.005331 1.005331
##
## $imp14
## b_acq_mean cond
## 1.001436 1.001436
##
## $imp15
## b_acq_mean cond
## 1.007394 1.007394
##
## $imp16
## b_acq_mean cond
## 1.009273 1.009273
##
## $imp17
## b_acq_mean cond
## 1.014007 1.014007
##
## $imp18
## b_acq_mean cond
## 1.007393 1.007393
##
## $imp19
## b_acq_mean cond
## 1.003475 1.003475
##
## $imp20
## b_acq_mean cond
## 1.007007 1.007007

*## Checking for influential observations in the data*

*# Do it once*
cook <- **cooks.distance**(acq_lms_no_summ_for_all_imps**$**imp1)
**plot**(cook, ylab="Cook's distances")

*## Do it for all imputations*

acq_cook_dist_for_all_imps <- **map**(acq_lms_no_summ_for_all_imps, **~**{
 cook <- **cooks.distance**(.x)
 **plot**(cook, ylab="Cook's distances")
})

## **Data Transformation (If Necessary) in Response to Violations of Assumptions**

We will run all models first without these transformations, and then, as a sensitivity analysis, run the models with these transformations

*## If residuals appear positively skewed, we'll do a square root transformation of the dependent variable*

acq_out_imputations_with_acq_diff_asump_vio <- **map**(acq_out**$**imputations, **~**{
 .x **%>%**
 **mutate**(pi_acq_mean_sqrt = **sqrt**(**abs**(pi_acq_mean)))
})

*## If residuals appear negatively skewed, we'll do a square root transformation of the dependent variable following a scale reversal*

acq_out_imputations_with_acq_diff_asump_vio <- **map**(acq_out**$**imputations, **~**{
 .x **%>%**
 **mutate**(pi_acq_mean_scale_reverse = dplyr**::recode**(pi_acq_mean, '0' = 5, '1' = 4, '2' = 3, '3' = 2, '4' = 1, '5' = 0),
 pi_acq_mean_scale_reverse_sqrt = **sqrt**(**abs**(pi_acq_mean_scale_reverse)))
})

*## If there are outliers as defined by greater than the 95th % away, we'll Winsorize those values*

acq_out_imputations_with_acq_diff_asump_vio <- **map**(acq_out**$**imputations, **~**{
 .x **%>%**
 **mutate**(pi_acq_mean_wins = **Winsorize**(pi_acq_mean))
})

## **For The Simulated Data, We’d Fail to Reject the Null Hypothesis for Hypothesis 3**

*## Now let's aggregate across all our imputations to get our "average" outcome for testing alternative hypothesis for hypothesis 3*

*## Now need to run linear regressions on all the dataframes*
*## (We were using Zelig but: 1. It's a deprecated package and I can't figure out what it's doing/it's documentation is all over the place AND 2. Doing this here allows us to run similar code for all hypotheses/check this against the Zelig output if necessary 3. We need to do something like this anyway to check all assumptions)*

acq_lms_for_all_imps <- **map**(acq_out**$**imputations, **~**{
 **summary**(**lm**(pi_acq_mean **~** b_acq_mean **+** cond, data = .x))
})

*## This gets a tidy version of estimate and standard error for all predictors + intercept for each regression*

acq_all_ests_for_all_imps_test <- **map**(acq_lms_for_all_imps, **~** **tidy**(.x))

*## Now I need to extract degrees of freedom for each linear regression (This has numerator and denominator)*
*## Haven't integrated this yet, since the df are the same across all imputed datasets. Can make this more funcitonal at some point in the future*

acq_df_for_all_imps <- **map**(acq_lms_for_all_imps, extract, **c**("df"))

*## Now have to extract specific estimate/standard error to eventually pass to the Amelia mi.meld function in order to aggregate the test statistics across imputed datasets according to Rubin's rules*

acq_int_order_ests_for_all_imps_test <- **map_df**(acq_all_ests_for_all_imps_test, **~**{
 .x **%>%**
 **filter**(term **==** "cond1") **%>%**
 dplyr**::select**(estimate,std.error)
}
)

*## Now pass those to the mi.meld function (Have to convert the columns/vectors to matrices to make this work)*

mi_avg_est_hyp_3_alt <- **mi.meld**(**as.matrix**(acq_int_order_ests_for_all_imps_test**$**estimate), **as.matrix**(acq_int_order_ests_for_all_imps_test**$**std.error), byrow = T)

*## This is the average estimate + the average standard error across imputeted datasets according to Rubin's rules*

mi_avg_est_hyp_3_alt**$**q.mi[1,1]

## [1] -0.05981329

mi_avg_est_hyp_3_alt**$**se.mi[1,1]

## [1] 0.1000843

*## Now let's calculate the t value by taking the esimate divided by the standard error*

mi_avg_t_value_hyp_3_alt <- mi_avg_est_hyp_3_alt**$**q.mi[1,1] **/** mi_avg_est_hyp_3_alt**$**se.mi[1,1]

*## And now the p value based on the just-calculated t value and degrees of freedom (Multiplied by 2 for a 2-tailed test) https://stats.stackexchange.com/questions/238427/why-the-lower-tail-f-is-used-when-mannualy-calculating-the-p-value-from-t-score*

p_value_hyp_3_alt <- 2***pt**(mi_avg_t_value_hyp_3_alt, df = 498, lower.tail = FALSE)
p_value_hyp_3_alt

## [1] 1.449641

*## Calculating d effect size with confidence intervals based on the t value using the MOTE package*
*## See also https://www.aggieerin.com/shiny-server/tests/indtt.html*
*## This effect size exactly matches what we were previously calculating from the Z-test from the Zelig package*

**d.ind.t.t**(mi_avg_t_value_hyp_3_alt, 250, 250, a = 0.0167) *# t value, n group 1, n group 2, alpha level*

## $d
## [1] -0.05356081
##
## $dlow
## [1] -0.2675236
##
## $dhigh
## [1] 0.1606701
##
## $n1
## [1] 250
##
## $n2
## [1] 250
##
## $df
## [1] 498
##
## $t
## [1] -0.5976292
##
## $p
## [1] 0.550359
##
## $estimate
## [1] "$d_s$ = -0.05, 98.33\\% CI [-0.27, 0.16]"
##
## $statistic
## [1] "$t$ = (498) = -0.60, $p$ = .550"

## **Directly Testing the Null of Hypotheis 3**

In this case using randomly simulated data we get a p = .01 for our equivalence test of the null for hypothesis one, so we would intepret this evidence as in support of the null hypothesis for hypothesis 3: Contain COVID Anxiety was equivalent or inferior to the Remain COVID Free SSI at improving perceived control over anxiety.

*# Now, assuming the above is null, we directly test the null hypothesis for Hypothesis 1 (i.e. can we really say there is no difference between SSIs OR that the placebo is superior?)*

*# In order to apply Rubin's rule for aggregating estimates/standard errors across all imputed datasets, we need to have estimates and standard errors across all of those datasets. Unfortunately, the TOSTER package doesn't output the info we need to follow this process. So, we'll now re-create the equivalence test from scratch (Starting with the t-test, which is the test the equivalence test is based upon) to get the info we need.*

*#Start with running a t-test on every imputed dataset after regressing out baseline for the variable of interest (In this case perceived control over anxiety). Note: I think, as was pointed out by Mallory, that we could probably literally use the same estimates and standard errors calculated above in this case, since the t-test of the diff_acq_mean after residualizing the b_acq_mean should be equivalent to the estimates we get from the regression above. But I've included this code both to be explicit about what we're doing and guard against any potential idiosyncratic differences between those estimates I might not know about.*

*## Do it once*

acq_out**$**imputations**$**imp1**$**diff_score_res = **lm**(pi_acq_mean **~** b_acq_mean **+** cond, data = acq_out**$**imputations**$**imp1)**$**residuals

t_test_example <- **t.test**(acq_out**$**imputations**$**imp1**$**diff_score_res **~** acq_out**$**imputations**$**imp1**$**cond, var.equal = T)

t_test_example**$**parameter *# degrees of freedom*

## df
## 498

t_test_example**$**estimate *# Estimates for both groups*

## mean in group 0 mean in group 1
## -1.316987e-16 8.841116e-17

t_test_example**$**stderr *# Standard error*

## [1] 0.07741277

*## Do it for all imputations*

*# First run a t-test on all dataframes and save the values*

acq_t_tests_hyp_3_for_all_imps <- **map**(acq_out**$**imputations, **~**{
 .x**$**diff_score_res = **lm**(pi_acq_mean **~** b_acq_mean **+** cond, data = .x)**$**residuals
 t_test_example <- **t.test**(.x**$**diff_score_res **~** .x**$**cond, var.equal = T)
})

*# Then extract the values we need (Using the tidy argument omits standard error)*

acq_t_tests_tidy_hyp_3_for_all_imps <- **map**(acq_t_tests_hyp_3_for_all_imps, **~** **tidy**(.x))

*# Using the tidy argument omits standard error from its output but we can calculate it based on the t ratio and the difference between the two sample means. T value statistic = estimate / standard error -> standard error = estimate / T value statistic. The standard error for imputation 1 using this method also matches when we calculate it manually above. We also map this to a dataframe rather than a list so we can extract the values easily in the next step.*

acq_t_tests_tidy_std_err_hyp_3_for_all_imps <- **map_df**(acq_t_tests_tidy_hyp_3_for_all_imps, **~**{
 .x **%>%**
 **mutate**(estimate = estimate2 **-** estimate1,
 std.error = **abs**(estimate **/** statistic))
})

*## Now have to put estimate and standard error into the mi.meld function to apply Rubin's rules for aggregating across multiple imputed datasets (Have to convert the columns/vectors to matrices to make this work)*

mi_avg_est_hyp_3_null <- **mi.meld**(**as.matrix**(acq_t_tests_tidy_std_err_hyp_3_for_all_imps**$**estimate), **as.matrix**(acq_t_tests_tidy_std_err_hyp_3_for_all_imps**$**std.error), byrow = T)

*## This is the average estimate + the average standard error across imputeted datasets according to Rubin's rules*

mi_avg_est_hyp_3_null**$**q.mi[1,1]

## [1] 7.478358e-17

mi_avg_est_hyp_3_null**$**se.mi[1,1]

## [1] 0.07751304

*# But we also need the pooled standard deviation to give the correct bounds for the equivalence test, since we have to multiply the d values of interest (defined ahead of time) by the pooled standard deviation to get the equivalence bounds we need in this circumstance*

*# Calculating pooled standard deviation from average standard error using this formula from Cochrane: https://handbook-5-1.cochrane.org/chapter_7/7_7_3_3_obtaining_standard_deviations_from_standard_errors.htm*

pooled_sd_hyp_3_null <- (mi_avg_est_hyp_3_null**$**se.mi[1,1])**/sqrt**(((1**/**250) **+** (1**/**250)))

*# Now create equivlanece bounds in scale units rather than d effect size units*

low_eqbound <- -0.63 ***** pooled_sd_hyp_3_null
high_eqbound <- 0.21 ***** pooled_sd_hyp_3_null

*# Let's create a lower bound t value*

t_low <- (mi_avg_est_hyp_3_null**$**q.mi[1,1] **+** low_eqbound)**/**mi_avg_est_hyp_3_null**$**se.mi[1,1]

*# And an upper bound t value*

t_high <- (mi_avg_est_hyp_3_null**$**q.mi[1,1] **+** high_eqbound)**/**mi_avg_est_hyp_3_null**$**se.mi[1,1]

*# Then compute p values for both*

p1 <- **pt**(t_low, 498, lower.tail = TRUE) *# P value for the low test, i.e. is the effect reliably greater than*
*## d = -0.63 (favoring the placebo)*
p2 <- **pt**(t_high, 498, lower.tail = FALSE) *# P vlue for the high test, i.e. is the effect reliably less than*
*## d = 0.21 (smallest effect size of interest favoring the intervention)*

*# And take the higher of those two as the final p value for the equivalence test*

**max**(p1, p2)

## [1] 0.009635709

## **Conducting a Unit Test to Validate Our From Scratch Equivalence Testing Procedure**

This is a unit test to validate that our method and the TOSTER package return the same results when applied to the same dataset. We focus on the first imputed dataset for this test, and our version returns the same result as the TOSTER test. Please see the in-chunk comments for more detail.

*## Let's unit test this by confirming with the equivalence test package to make sure my method gets the same answer on the same dataset. We'll use imp1 as we've been using that as our test throughout*

df_equiv <- acq_out**$**imputations**$**imp1 **%>%**
 **mutate**(
 diff_score_res = **lm**(pi_acq_mean **~** b_acq_mean)**$**residuals *#creating a dataframe with residuals for ACQ residual scores*
 ) **%>%**
 dplyr**::group_by**(cond) **%>%** *#grouping by SSI condition*
 **summarise**(
 mean_diff_score_res = **mean**(diff_score_res), *#we'll be looking at means and sds in each group*
 sd_diff_score_res = **sd**(diff_score_res)
 )

**TOSTtwo**(m1 = df_equiv[[1,2]], m2 = df_equiv[[2,2]], sd1 = df_equiv[[1,3]], sd2 = df_equiv[[2,3]],
 n1 = 250, n2 = 250, low_eqbound_d = -0.63, high_eqbound_d = 0.21, alpha = 0.0167,
 var.equal = TRUE, plot = FALSE, verbose = TRUE)

## TOST results:
## t-value lower bound: 6.88 p-value lower bound: 0.000000000009
## t-value upper bound: -2.51 p-value upper bound: 0.006
## degrees of freedom : 498
##
## Equivalence bounds (Cohen's d):
## low eqbound: -0.63
## high eqbound: 0.21
##
## Equivalence bounds (raw scores):
## low eqbound: -0.5452
## high eqbound: 0.1817
##
## TOST confidence interval:
## lower bound 96.66% CI: -0.178
## upper bound 96.66% CI: 0.152
##
## NHST confidence interval:
## lower bound 98.33% CI: -0.199
## upper bound 98.33% CI: 0.173
##
## Equivalence Test Result:
## The equivalence test was significant, t(498) = -2.515, p = 0.00611, given equivalence bounds of -0.545 and 0.182 (on a raw scale) and an alpha of 0.0167.
## Null Hypothesis Test Result:
## The null hypothesis test was non-significant, t(498) = -0.167, p = 0.868, given an alpha of 0.0167.
## Based on the equivalence test and the null-hypothesis test combined, we can conclude that the observed effect is statistically not different from zero and statistically equivalent to zero.

*## Now back to our method*

acq_out**$**imputations**$**imp1**$**diff_score_res = **lm**(pi_acq_mean **~** b_acq_mean, data = acq_out**$**imputations**$**imp1)**$**residuals

t_test_example <- **t.test**(acq_out**$**imputations**$**imp1**$**diff_score_res **~** acq_out**$**imputations**$**imp1**$**cond, var.equal = T)

*## We're getting the same t-value and p-value as the t-test conducted above (Not the equivalence test yet)*

tidy_t_test_example <- **tidy**(t_test_example)

*## How do the estimates match up? They match exactly*

tidy_t_test_example_w_estimate <- tidy_t_test_example **%>%**
 **mutate**(estimate = estimate2 **-** estimate1,
 std.error = **abs**(estimate **/** statistic))

df_equiv[[1,2]]

## [1] -0.006434335

tidy_t_test_example_w_estimate**$**estimate1

## [1] -0.006434335

df_equiv[[2,2]]

## [1] 0.006486017

tidy_t_test_example_w_estimate**$**estimate2

## [1] 0.006486017

pooled_sd_hyp_1_null_t_test_example <- (tidy_t_test_example_w_estimate**$**std.error)**/sqrt**(((1**/**250) **+** (1**/**250)))

*# Now create equivlanece bounds in scale units rather than d effect size units*
*## These are exactly the same as above at the thousandths decimal place.*

low_eqbound_t_test_example <- -0.63 ***** pooled_sd_hyp_1_null_t_test_example
high_eqbound_t_test_example <- 0.21 ***** pooled_sd_hyp_1_null_t_test_example

*# Let's create a lower bound t value*

*# To get matching results with the TOSTER package we need to add the equivalence bounds to the estimate values . Once we do this we get the exact same t values as the TOSTER package.*

t_low_t_test_example <- (tidy_t_test_example_w_estimate**$**estimate **+** low_eqbound_t_test_example)**/**tidy_t_test_example_w_estimate**$**std.error

*# And an upper bound t value*

t_high_t_test_example <- (tidy_t_test_example_w_estimate**$**estimate **+** high_eqbound_t_test_example)**/**tidy_t_test_example_w_estimate**$**std.error

*# Then compute p values for both*

*# In order to match the p value from our output to the TOSTER package output, we need to have lower.tail = TRUE for the lower bound example and lower.tail = FALSE for the high bound example.*

p1 <- **pt**(t_low_t_test_example, 498, lower.tail = TRUE) *# P value for the low test, i.e. is the effect reliably greater than*
*## d = -0.63 (favoring the placebo)*
p2 <- **pt**(t_high_t_test_example, 498, lower.tail = FALSE)

**max**(p1, p2)

## [1] 0.006112411

## **Sensitivity Tests for Hypotheses: What If Everyone Who Dropped Out Was in the 75th Percentile of Change for Confirmatory Outcomes? Or the 25th Percentile of Change?**

*## Need to calculate the 25% of change and 75% of change and then puts those values into two new varables and runs linear regressions/equivalence tests with those as the final variables of interest instead*

*# Hypothesis 1*

res_change_gad_7 <- **lm**(f_gad_7_mean **~** b_gad_7_mean, data = acq_imp)**$**residuals

diff_perc_change_gad_7 <- **quantile**(res_change_gad_7,**c**(0.25,0.75))

diff_perc_change_gad_7[[1]] *# 25%*

## [1] -0.3270019

diff_perc_change_gad_7[[2]] *# 75%*

## [1] 0.32821

data_test_sens <- acq_imp **%>%**
 **mutate**(f_gad_7_mean_25_per_change = **case_when**(
 **is.na**(f_gad_7_mean) **~** diff_perc_change_gad_7[[1]],
 TRUE **~** f_gad_7_mean),
 f_gad_7_mean_75_per_change = **case_when**(
 **is.na**(f_gad_7_mean) **~** diff_perc_change_gad_7[[2]],
 TRUE **~** f_gad_7_mean
 )
 )

data_test_sens **%>%**
 **group_by**(b_gad_7_mean) **%>%**
 **tally**()

## # A tibble: 17 x 2
## b_gad_7_mean n
## <dbl> <int>
## 1 0.429 2
## 2 0.571 2
## 3 0.714 10
## 4 0.857 25
## 5 1 15
## 6 1.14 38
## 7 1.29 44
## 8 1.43 58
## 9 1.57 50
## 10 1.71 58
## 11 1.86 28
## 12 2 28
## 13 2.14 25
## 14 2.29 11
## 15 2.43 4
## 16 2.57 3
## 17 NA 99

lm_summary_25_per_change <- **summary**(**lm**(f_gad_7_mean_25_per_change **~** cond, data = data_test_sens))
lm_summary_75_per_change <- **summary**(**lm**(f_gad_7_mean_75_per_change **~** cond, data = data_test_sens))

data_test_sens_equiv_25_perc <- data_test_sens **%>%**
 **mutate**(
 diff_score_res = f_gad_7_mean_25_per_change *#creating a dataframe with residuals for ACQ residual scores*
 ) **%>%**
 dplyr**::group_by**(cond) **%>%** *#grouping by SSI condition*
 **summarise**(
 mean_diff_score_res = **mean**(diff_score_res), *#we'll be looking at means and sds in each group*
 sd_diff_score_res = **sd**(diff_score_res)
 )

equiv_summary_25_per_change <- **TOSTtwo**(m1 = data_test_sens_equiv_25_perc[[1,2]], m2 = data_test_sens_equiv_25_perc[[2,2]], sd1 = data_test_sens_equiv_25_perc[[1,3]], sd2 = data_test_sens_equiv_25_perc[[2,3]],
 n1 = 250, n2 = 250, low_eqbound_d = -0.33, high_eqbound_d = 0.33, alpha = 0.0167,
 var.equal = TRUE, plot = FALSE, verbose = TRUE)

## TOST results:
## t-value lower bound: 3.67 p-value lower bound: 0.0001
## t-value upper bound: -3.71 p-value upper bound: 0.0001
## degrees of freedom : 498
##
## Equivalence bounds (Cohen's d):
## low eqbound: -0.33
## high eqbound: 0.33
##
## Equivalence bounds (raw scores):
## low eqbound: -0.2839
## high eqbound: 0.2839
##
## TOST confidence interval:
## lower bound 96.66% CI: -0.166
## upper bound 96.66% CI: 0.162
##
## NHST confidence interval:
## lower bound 98.33% CI: -0.186
## upper bound 98.33% CI: 0.183
##
## Equivalence Test Result:
## The equivalence test was significant, t(498) = 3.668, p = 0.000136, given equivalence bounds of -0.284 and 0.284 (on a raw scale) and an alpha of 0.0167.
## Null Hypothesis Test Result:
## The null hypothesis test was non-significant, t(498) = -0.0219, p = 0.983, given an alpha of 0.0167.
## Based on the equivalence test and the null-hypothesis test combined, we can conclude that the observed effect is statistically not different from zero and statistically equivalent to zero.

data_test_sens_equiv_75_perc <- data_test_sens **%>%**
 **mutate**(
 diff_score_res = f_gad_7_mean_75_per_change *#creating a dataframe with residuals for ACQ residual scores*
 ) **%>%**
 dplyr**::group_by**(cond) **%>%** *#grouping by SSI condition*
 **summarise**(
 mean_diff_score_res = **mean**(diff_score_res), *#we'll be looking at means and sds in each group*
 sd_diff_score_res = **sd**(diff_score_res)
 )

equiv_summary_75_per_change <- **TOSTtwo**(m1 = data_test_sens_equiv_75_perc[[1,2]], m2 = data_test_sens_equiv_75_perc[[2,2]], sd1 = data_test_sens_equiv_75_perc[[1,3]], sd2 = data_test_sens_equiv_75_perc[[2,3]],
 n1 = 250, n2 = 250, low_eqbound_d = -0.33, high_eqbound_d = 0.33, alpha = 0.0167,
 var.equal = TRUE, plot = FALSE, verbose = TRUE)

## TOST results:
## t-value lower bound: 3.81 p-value lower bound: 0.00008
## t-value upper bound: -3.57 p-value upper bound: 0.0002
## degrees of freedom : 498
##
## Equivalence bounds (Cohen's d):
## low eqbound: -0.33
## high eqbound: 0.33
##
## Equivalence bounds (raw scores):
## low eqbound: -0.205
## high eqbound: 0.205
##
## TOST confidence interval:
## lower bound 96.66% CI: -0.112
## upper bound 96.66% CI: 0.125
##
## NHST confidence interval:
## lower bound 98.33% CI: -0.127
## upper bound 98.33% CI: 0.14
##
## Equivalence Test Result:
## The equivalence test was significant, t(498) = -3.574, p = 0.000193, given equivalence bounds of -0.205 and 0.205 (on a raw scale) and an alpha of 0.0167.
## Null Hypothesis Test Result:
## The null hypothesis test was non-significant, t(498) = 0.116, p = 0.908, given an alpha of 0.0167.
## Based on the equivalence test and the null-hypothesis test combined, we can conclude that the observed effect is statistically not different from zero and statistically equivalent to zero.

sens_summary_list_hyp_1 <- **list**(lm_summary_25_per_change, lm_summary_75_per_change, equiv_summary_25_per_change, equiv_summary_75_per_change)

sens_summary_list_hyp_1

## [[1]]
##
## Call:
## lm(formula = f_gad_7_mean_25_per_change ~ cond, data = data_test_sens)
##
## Residuals:
## Min 1Q Median 3Q Max
## -1.4156 -0.3373 0.1988 0.6274 1.6274
##
## Coefficients:
## Estimate Std. Error t value Pr(>|t|)
## (Intercept) 1.086880 0.061130 17.78 <2e-16 ***
## cond1 0.001687 0.086235 0.02 0.984
## ---
## Signif. codes: 0 '***' 0.001 '**' 0.01 '*' 0.05 '.' 0.1 ' ' 1
##
## Residual standard error: 0.8602 on 396 degrees of freedom
## (102 observations deleted due to missingness)
## Multiple R-squared: 9.663e-07, Adjusted R-squared: -0.002524
## F-statistic: 0.0003827 on 1 and 396 DF, p-value: 0.9844
##
##
## [[2]]
##
## Call:
## lm(formula = f_gad_7_mean_75_per_change ~ cond, data = data_test_sens)
##
## Residuals:
## Min 1Q Median 3Q Max
## -0.91420 -0.48760 0.04972 0.47830 1.47188
##
## Coefficients:
## Estimate Std. Error t value Pr(>|t|)
## (Intercept) 1.24241 0.04415 28.143 <2e-16 ***
## cond1 -0.00642 0.06228 -0.103 0.918
## ---
## Signif. codes: 0 '***' 0.001 '**' 0.01 '*' 0.05 '.' 0.1 ' ' 1
##
## Residual standard error: 0.6212 on 396 degrees of freedom
## (102 observations deleted due to missingness)
## Multiple R-squared: 2.684e-05, Adjusted R-squared: -0.002498
## F-statistic: 0.01063 on 1 and 396 DF, p-value: 0.9179
##
##
## [[3]]
## [[3]]$diff
## [1] -0.001686929
##
## [[3]]$TOST_t1
## [1] 3.667588
##
## [[3]]$TOST_p1
## [1] 0.000135604
##
## [[3]]$TOST_t2
## [1] -3.711436
##
## [[3]]$TOST_p2
## [1] 0.0001146646
##
## [[3]]$TOST_df
## [1] 498
##
## [[3]]$alpha
## [1] 0.0167
##
## [[3]]$low_eqbound
## [1] -0.283888
##
## [[3]]$high_eqbound
## [1] 0.283888
##
## [[3]]$low_eqbound_d
## [1] -0.33
##
## [[3]]$high_eqbound_d
## [1] 0.33
##
## [[3]]$LL_CI_TOST
## [1] -0.1658219
##
## [[3]]$UL_CI_TOST
## [1] 0.1624481
##
## [[3]]$LL_CI_TTEST
## [1] -0.1864583
##
## [[3]]$UL_CI_TTEST
## [1] 0.1830844
##
##
## [[4]]
## [[4]]$diff
## [1] 0.006420492
##
## [[4]]$TOST_t1
## [1] 3.805055
##
## [[4]]$TOST_p1
## [1] 7.970457e-05
##
## [[4]]$TOST_t2
## [1] -3.57397
##
## [[4]]$TOST_p2
## [1] 0.0001929181
##
## [[4]]$TOST_df
## [1] 498
##
## [[4]]$alpha
## [1] 0.0167
##
## [[4]]$low_eqbound
## [1] -0.2050197
##
## [[4]]$high_eqbound
## [1] 0.2050197
##
## [[4]]$low_eqbound_d
## [1] -0.33
##
## [[4]]$high_eqbound_d
## [1] 0.33
##
## [[4]]$LL_CI_TOST
## [1] -0.1121153
##
## [[4]]$UL_CI_TOST
## [1] 0.1249563
##
## [[4]]$LL_CI_TTEST
## [1] -0.1270186
##
## [[4]]$UL_CI_TTEST
## [1] 0.1398596

*## Need to calculate the 25% of change and 75% of change and then puts those values into two new varables and runs lequivalence tests (with built-in t-tests) with those as the final variables of interest instead*

*# Hypothesis 2*

perc_b_soc_dist <- **quantile**(soc_dist_imp**$**b_soc_dist_mean,**c**(0.25,0.75), na.rm = T)
perc_pi_soc_dist <- **quantile**(soc_dist_imp**$**pi_soc_dist_mean,**c**(0.25,0.75), na.rm = T)

perc_b_soc_dist[[1]] *# 25%*

## [1] 2

perc_b_soc_dist[[2]] *# 75%*

## [1] 3

data_test_sens <- soc_dist_imp **%>%**
 **mutate**(pi_soc_dist_mean_25_per = **case_when**(
 **is.na**(pi_soc_dist_mean) **~** perc_pi_soc_dist[[1]],
 TRUE **~** pi_soc_dist_mean),
 pi_soc_dist_mean_75_per = **case_when**(
 **is.na**(pi_soc_dist_mean) **~** perc_pi_soc_dist[[2]],
 TRUE **~** pi_soc_dist_mean
 ),
 b_soc_dist_mean_25_per = **case_when**(
 **is.na**(b_soc_dist_mean) **~** perc_b_soc_dist[[1]],
 TRUE **~** b_soc_dist_mean),
 b_soc_dist_mean_75_per = **case_when**(
 **is.na**(b_soc_dist_mean) **~** perc_b_soc_dist[[2]],
 TRUE **~** b_soc_dist_mean
 )
 )

data_test_sens_equiv_25_perc <- data_test_sens **%>%**
 **filter**(cond **==** 1) **%>%** *#grouping by SSI condition*
 **summarise**(
 b_mean = **mean**(b_soc_dist_mean_25_per), *#we'll be looking at means and sds in each group*
 pi_mean = **mean**(pi_soc_dist_mean_25_per),
 b_sd = **sd**(b_soc_dist_mean_25_per),
 pi_sd = **sd**(pi_soc_dist_mean_25_per)
 )

cor_test_sens_equiv_25_perc <- **cor**(data_test_sens**$**b_soc_dist_mean_25_per,data_test_sens**$**pi_soc_dist_mean_25_per)

equiv_summary_25_per_change <- **TOSTpaired**(m1 = data_test_sens_equiv_25_perc[[1,1]], m2 = data_test_sens_equiv_25_perc[[1,2]], sd1 = data_test_sens_equiv_25_perc[[1,3]], sd2 = data_test_sens_equiv_25_perc[[1,4]],
 n = 250, low_eqbound_dz = -0.33, high_eqbound_dz = 0.33, r12 = cor_test_sens_equiv_25_perc, alpha = 0.0167, plot = FALSE, verbose = TRUE)

## TOST results:
## t-value lower bound: 5.09 p-value lower bound: 0.0000003
## t-value upper bound: -5.34 p-value upper bound: 0.0000001
## degrees of freedom : 249
##
## Equivalence bounds (Cohen's dz):
## low eqbound: -0.33
## high eqbound: 0.33
##
## Equivalence bounds (raw scores):
## low eqbound: -0.2855
## high eqbound: 0.2855
##
## TOST confidence interval:
## lower bound 96.66% CI: -0.124
## upper bound 96.66% CI: 0.11
##
## NHST confidence interval:
## lower bound 98.33% CI: -0.139
## upper bound 98.33% CI: 0.125
##
## Equivalence Test Result:
## The equivalence test was significant, t(249) = 5.092, p = 0.00000035, given equivalence bounds of -0.285 and 0.285 (on a raw scale) and an alpha of 0.0167.
## Null Hypothesis Test Result:
## The null hypothesis test was non-significant, t(249) = -0.126, p = 0.900, given an alpha of 0.0167.
## Based on the equivalence test and the null-hypothesis test combined, we can conclude that the observed effect is statistically not different from zero and statistically equivalent to zero.

data_test_sens_equiv_75_perc <- data_test_sens **%>%**
 **filter**(cond **==** 1) **%>%** *#grouping by SSI condition*
 **summarise**(
 b_mean = **mean**(b_soc_dist_mean_75_per), *#we'll be looking at means and sds in each group*
 pi_mean = **mean**(pi_soc_dist_mean_75_per),
 b_sd = **sd**(b_soc_dist_mean_75_per),
 pi_sd = **sd**(pi_soc_dist_mean_75_per)
 )

cor_test_sens_equiv_75_perc <- **cor**(data_test_sens**$**b_soc_dist_mean_75_per,data_test_sens**$**pi_soc_dist_mean_75_per)

equiv_summary_75_per_change <- **TOSTpaired**(m1 = data_test_sens_equiv_75_perc[[1,1]], m2 = data_test_sens_equiv_75_perc[[1,2]], sd1 = data_test_sens_equiv_75_perc[[1,3]], sd2 = data_test_sens_equiv_75_perc[[1,4]],
 n = 250, low_eqbound_dz = -0.33, high_eqbound_dz = 0.33, r12 = cor_test_sens_equiv_75_perc, alpha = 0.0167, plot = FALSE, verbose = TRUE)

## TOST results:
## t-value lower bound: 4.62 p-value lower bound: 0.000003
## t-value upper bound: -5.82 p-value upper bound: 0.000000009
## degrees of freedom : 249
##
## Equivalence bounds (Cohen's dz):
## low eqbound: -0.33
## high eqbound: 0.33
##
## Equivalence bounds (raw scores):
## low eqbound: -0.2836
## high eqbound: 0.2836
##
## TOST confidence interval:
## lower bound 96.66% CI: -0.149
## upper bound 96.66% CI: 0.084
##
## NHST confidence interval:
## lower bound 98.33% CI: -0.164
## upper bound 98.33% CI: 0.098
##
## Equivalence Test Result:
## The equivalence test was significant, t(249) = 4.617, p = 0.00000312, given equivalence bounds of -0.284 and 0.284 (on a raw scale) and an alpha of 0.0167.
## Null Hypothesis Test Result:
## The null hypothesis test was non-significant, t(249) = -0.601, p = 0.549, given an alpha of 0.0167.
## Based on the equivalence test and the null-hypothesis test combined, we can conclude that the observed effect is statistically not different from zero and statistically equivalent to zero.

sens_summary_list_hyp_2 <- **list**(equiv_summary_25_per_change, equiv_summary_75_per_change)

sens_summary_list_hyp_2

## [[1]]
## [[1]]$diff
## [1] -0.006872852
##
## [[1]]$TOST_t1
## [1] 5.092141
##
## [[1]]$TOST_p1
## [1] 3.495372e-07
##
## [[1]]$TOST_t2
## [1] -5.343375
##
## [[1]]$TOST_p2
## [1] 1.029849e-07
##
## [[1]]$TOST_df
## [1] 249
##
## [[1]]$alpha
## [1] 0.0167
##
## [[1]]$low_eqbound
## [1] -0.2854777
##
## [[1]]$high_eqbound
## [1] 0.2854777
##
## [[1]]$low_eqbound_dz
## [1] -0.33
##
## [[1]]$high_eqbound_dz
## [1] 0.33
##
## [[1]]$LL_CI_TOST
## [1] -0.1239092
##
## [[1]]$UL_CI_TOST
## [1] 0.1101634
##
## [[1]]$LL_CI_TTEST
## [1] -0.1387041
##
## [[1]]$UL_CI_TTEST
## [1] 0.1249584
##
##
## [[2]]
## [[2]]$diff
## [1] -0.03264605
##
## [[2]]$TOST_t1
## [1] 4.61722
##
## [[2]]$TOST_p1
## [1] 3.118104e-06
##
## [[2]]$TOST_t2
## [1] -5.818297
##
## [[2]]$TOST_p2
## [1] 9.099701e-09
##
## [[2]]$TOST_df
## [1] 249
##
## [[2]]$alpha
## [1] 0.0167
##
## [[2]]$low_eqbound
## [1] -0.2836441
##
## [[2]]$high_eqbound
## [1] 0.2836441
##
## [[2]]$low_eqbound_dz
## [1] -0.33
##
## [[2]]$high_eqbound_dz
## [1] 0.33
##
## [[2]]$LL_CI_TOST
## [1] -0.1489306
##
## [[2]]$UL_CI_TOST
## [1] 0.08363852
##
## [[2]]$LL_CI_TTEST
## [1] -0.1636305
##
## [[2]]$UL_CI_TTEST
## [1] 0.0983384

*## Need to calculate the 25% of change and 75% of change and then puts those values into two new varables and runs linear regressions/equivalence tests with those as the final variables of interest instead*

*# Hypothesis 3*

res_change_acq <- **lm**(pi_acq_mean **~** b_acq_mean, data = acq_imp)**$**residuals

diff_perc_change_acq <- **quantile**(res_change_acq,**c**(0.25,0.75))

diff_perc_change_acq[[1]] *# 25%*

## [1] -0.6037839

diff_perc_change_acq[[2]] *# 75%*

## [1] 0.5737906

data_test_sens <- acq_imp **%>%**
 **mutate**(pi_acq_mean_25_per_change = **case_when**(
 **is.na**(pi_acq_mean) **~** diff_perc_change_acq[[1]],
 TRUE **~** pi_acq_mean),
 pi_acq_mean_75_per_change = **case_when**(
 **is.na**(pi_acq_mean) **~** diff_perc_change_acq[[2]],
 TRUE **~** pi_acq_mean
 )
 )

lm_summary_25_per_change <- **summary**(**lm**(pi_acq_mean_25_per_change **~** cond, data = data_test_sens))
lm_summary_75_per_change <- **summary**(**lm**(pi_acq_mean_75_per_change **~** cond, data = data_test_sens))

data_test_sens_equiv_25_perc <- data_test_sens **%>%**
 **mutate**(
 diff_score_res = pi_acq_mean_25_per_change *#creating a dataframe with residuals for ACQ residual scores*
 ) **%>%**
 dplyr**::group_by**(cond) **%>%** *#grouping by SSI condition*
 **summarise**(
 mean_diff_score_res = **mean**(diff_score_res), *#we'll be looking at means and sds in each group*
 sd_diff_score_res = **sd**(diff_score_res)
 )

equiv_summary_25_per_change <- **TOSTtwo**(m1 = data_test_sens_equiv_25_perc[[1,2]], m2 = data_test_sens_equiv_25_perc[[2,2]], sd1 = data_test_sens_equiv_25_perc[[1,3]], sd2 = data_test_sens_equiv_25_perc[[2,3]],
 n1 = 250, n2 = 250, low_eqbound_d = -0.33, high_eqbound_d = 0.33, alpha = 0.0167,
 var.equal = TRUE, plot = FALSE, verbose = TRUE)

## TOST results:
## t-value lower bound: 3.60 p-value lower bound: 0.0002
## t-value upper bound: -3.78 p-value upper bound: 0.00009
## degrees of freedom : 498
##
## Equivalence bounds (Cohen's d):
## low eqbound: -0.33
## high eqbound: 0.33
##
## Equivalence bounds (raw scores):
## low eqbound: -0.4644
## high eqbound: 0.4644
##
## TOST confidence interval:
## lower bound 96.66% CI: -0.28
## upper bound 96.66% CI: 0.257
##
## NHST confidence interval:
## lower bound 98.33% CI: -0.313
## upper bound 98.33% CI: 0.291
##
## Equivalence Test Result:
## The equivalence test was significant, t(498) = 3.600, p = 0.000175, given equivalence bounds of -0.464 and 0.464 (on a raw scale) and an alpha of 0.0167.
## Null Hypothesis Test Result:
## The null hypothesis test was non-significant, t(498) = -0.0893, p = 0.929, given an alpha of 0.0167.
## Based on the equivalence test and the null-hypothesis test combined, we can conclude that the observed effect is statistically not different from zero and statistically equivalent to zero.

data_test_sens_equiv_75_perc <- data_test_sens **%>%**
 **mutate**(
 diff_score_res = pi_acq_mean_75_per_change *#creating a dataframe with residuals for ACQ residual scores*
 ) **%>%**
 dplyr**::group_by**(cond) **%>%** *#grouping by SSI condition*
 **summarise**(
 mean_diff_score_res = **mean**(diff_score_res), *#we'll be looking at means and sds in each group*
 sd_diff_score_res = **sd**(diff_score_res)
 )

equiv_summary_75_per_change <- **TOSTtwo**(m1 = data_test_sens_equiv_75_perc[[1,2]], m2 = data_test_sens_equiv_75_perc[[2,2]], sd1 = data_test_sens_equiv_75_perc[[1,3]], sd2 = data_test_sens_equiv_75_perc[[2,3]],
 n1 = 250, n2 = 250, low_eqbound_d = -0.33, high_eqbound_d = 0.33, alpha = 0.0167,
 var.equal = TRUE, plot = FALSE, verbose = TRUE)

## TOST results:
## t-value lower bound: 3.78 p-value lower bound: 0.00009
## t-value upper bound: -3.59 p-value upper bound: 0.0002
## degrees of freedom : 498
##
## Equivalence bounds (Cohen's d):
## low eqbound: -0.33
## high eqbound: 0.33
##
## Equivalence bounds (raw scores):
## low eqbound: -0.3427
## high eqbound: 0.3427
##
## TOST confidence interval:
## lower bound 96.66% CI: -0.189
## upper bound 96.66% CI: 0.207
##
## NHST confidence interval:
## lower bound 98.33% CI: -0.214
## upper bound 98.33% CI: 0.232
##
## Equivalence Test Result:
## The equivalence test was significant, t(498) = -3.594, p = 0.000179, given equivalence bounds of -0.343 and 0.343 (on a raw scale) and an alpha of 0.0167.
## Null Hypothesis Test Result:
## The null hypothesis test was non-significant, t(498) = 0.0954, p = 0.924, given an alpha of 0.0167.
## Based on the equivalence test and the null-hypothesis test combined, we can conclude that the observed effect is statistically not different from zero and statistically equivalent to zero.

sens_summary_list_hyp_3 <- **list**(lm_summary_25_per_change, lm_summary_75_per_change, equiv_summary_25_per_change, equiv_summary_75_per_change)

sens_summary_list_hyp_3

## [[1]]
##
## Call:
## lm(formula = pi_acq_mean_25_per_change ~ cond, data = data_test_sens)
##
## Residuals:
## Min 1Q Median 3Q Max
## -2.3966 -0.7815 0.4572 0.9685 2.4685
##
## Coefficients:
## Estimate Std. Error t value Pr(>|t|)
## (Intercept) 1.78154 0.10000 17.82 <2e-16 ***
## cond1 0.01124 0.14107 0.08 0.937
## ---
## Signif. codes: 0 '***' 0.001 '**' 0.01 '*' 0.05 '.' 0.1 ' ' 1
##
## Residual standard error: 1.407 on 396 degrees of freedom
## (102 observations deleted due to missingness)
## Multiple R-squared: 1.604e-05, Adjusted R-squared: -0.002509
## F-statistic: 0.00635 on 1 and 396 DF, p-value: 0.9365
##
##
## [[2]]
##
## Call:
## lm(formula = pi_acq_mean_75_per_change ~ cond, data = data_test_sens)
##
## Residuals:
## Min 1Q Median 3Q Max
## -2.0165 -1.0165 0.2246 0.7335 2.2335
##
## Coefficients:
## Estimate Std. Error t value Pr(>|t|)
## (Intercept) 2.025381 0.073797 27.445 <2e-16 ***
## cond1 -0.008861 0.104103 -0.085 0.932
## ---
## Signif. codes: 0 '***' 0.001 '**' 0.01 '*' 0.05 '.' 0.1 ' ' 1
##
## Residual standard error: 1.038 on 396 degrees of freedom
## (102 observations deleted due to missingness)
## Multiple R-squared: 1.829e-05, Adjusted R-squared: -0.002507
## F-statistic: 0.007244 on 1 and 396 DF, p-value: 0.9322
##
##
## [[3]]
## [[3]]$diff
## [1] -0.01124136
##
## [[3]]$TOST_t1
## [1] 3.600198
##
## [[3]]$TOST_p1
## [1] 0.0001749098
##
## [[3]]$TOST_t2
## [1] -3.778827
##
## [[3]]$TOST_p2
## [1] 8.832124e-05
##
## [[3]]$TOST_df
## [1] 498
##
## [[3]]$alpha
## [1] 0.0167
##
## [[3]]$low_eqbound
## [1] -0.4643715
##
## [[3]]$high_eqbound
## [1] 0.4643715
##
## [[3]]$low_eqbound_d
## [1] -0.33
##
## [[3]]$high_eqbound_d
## [1] 0.33
##
## [[3]]$LL_CI_TOST
## [1] -0.2797261
##
## [[3]]$UL_CI_TOST
## [1] 0.2572434
##
## [[3]]$LL_CI_TTEST
## [1] -0.3134822
##
## [[3]]$UL_CI_TTEST
## [1] 0.2909995
##
##
## [[4]]
## [[4]]$diff
## [1] 0.008860668
##
## [[4]]$TOST_t1
## [1] 3.784911
##
## [[4]]$TOST_p1
## [1] 8.624742e-05
##
## [[4]]$TOST_t2
## [1] -3.594113
##
## [[4]]$TOST_p2
## [1] 0.0001789411
##
## [[4]]$TOST_df
## [1] 498
##
## [[4]]$alpha
## [1] 0.0167
##
## [[4]]$low_eqbound
## [1] -0.3426826
##
## [[4]]$high_eqbound
## [1] 0.3426826
##
## [[4]]$low_eqbound_d
## [1] -0.33
##
## [[4]]$high_eqbound_d
## [1] 0.33
##
## [[4]]$LL_CI_TOST
## [1] -0.1892675
##
## [[4]]$UL_CI_TOST
## [1] 0.2069888
##
## [[4]]$LL_CI_TTEST
## [1] -0.2141777
##
## [[4]]$UL_CI_TTEST
## [1] 0.2318991
